# Supplementary material for: Dual stimuli-responsive rotaxane-branched dendrimers with reversible dimension modulation
Source: Nat Commun. 2018 Aug 9;9:3190. doi: 10.1038/s41467-018-05670-y (PMC6085385; doi:10.1038/s41467-018-05670-y)
Supplement: Supplementary file 1 — Supplementary Information [file 41467_2018_5670_MOESM1_ESM.pdf]

Dual Stimuli-responsive Rotaxane-branched Dendrimers with Reversible Dimension Modulation

Xu-Qing Wang,[a†] Wei Wang,[a†] Wei-Jian Li,[a] Li-Jun Chen,[a] Rui Yao,[a] Guang-Qiang Yin,[a,b] Yu-Xuan Wang,[a] Ying Zhang,[c] Junlin Huang,[d] Hongwei Tan,[c] Yihua Yu,[d] Xiaopeng Li,[b] Lin Xu,[a,*] Hai-Bo Yang[a,*]

[a] Shanghai Key Laboratory of Green Chemistry and Chemical Processes, School of Chemistry and Molecular Engineering, Chang-Kung Chuang Institute, East China Normal University, Shanghai 200062, P. R. China.

[b] Department of Chemistry, University of South Florida, Tampa, Florida 33620, United States.

[c] Department of Chemistry, Beijing Normal University, Beijing 100050, P. R. China.

[d] Shanghai Key Laboratory of Magnetic Resonance, Department of Physics, East China Normal University, Shanghai 200062, P. R. China

[†] These authors contributed equally to this work

To whom correspondence should be addressed; e-mail: lxu@chem.ecnu.edu.cn, hbyang@chem.ecnu.edu.cn

Table of Contents (77 Pages)

Section A. Supplementary Materials and methods S3

Section B. Synthesis and characterizaiton of [2]rotaxane 2 S4

Section C. Synthesis and characterizaiton of the rotaxane-branched dendrimers G_1-G_3 S19

1. *Synthesis of the rotaxane-branched dendrimers* ***G_1–G_3*** S19

2 *NMR, MS and GPC spectra of the rotaxane-branched dendrimers* ***G_1–G_3*** S24

3. *2-D DOSY spectra of the rotaxane-branched dendrimers* ***G_1–G_3*** S36

4. *AFM and TEM images for the rotaxane-branched dendrimers* ***G_1-G_3*** S39

6 *UV-vis spectra of the rotaxane-branched dendrimers* ***G_1–G_3*** S41

Section D. Synthesis and characterizaiton of model dendrimers S42

Section E. Solvent and anion-induced switching of [2]rotaxane building block 2 S51

Section F. Solvent and anion-induced switching of the rotaxane-branched dendrimers S59

Section G. Supplementary References S77

Section A. Supplementary Materials and Methods

All reagents were commercially available and used as supplied without further purification, compounds **S_1**, **S_2** and 4-diethoxypillar[5]arene (DEpillar[5]arene) were prepared according to the published procedures.[S1-S3] Deuterated solvents were purchased from Cambridge Isotope Laboratory (Andover, MA).

All solvents were dried according to standard procedures and all of them were degassed under N_2 for 30 minutes before use. All air-sensitive reactions were carried out under inert N_2 atmosphere. ^{1}H NMR, ^{13}C NMR and ^{31}P NMR spectra were recorded on Bruker 300 MHz Spectrometer (^{1}H: 300 MHz; 31P: 121.4 MHz) and Bruker 400 MHz Spectrometer (^{1}H: 400 MHz; ^{13}C: 100 MHz, ^{31}P: 161.9 MHz) at 298 K. The ^{1}H and ^{13}C NMR chemical shifts are reported relative to residual solvent signals, and ^{31}P {^{1}H} NMR chemical shifts are referenced to an external unlocked sample of 85% H_3PO_4 (δ 0.0). 2D NMR spectra (^{1}H-^{1}H COSY, ROESY and DOSY) were recorded on Bruker 500 MHz Spectrometer (^{1}H: 500 MHz) at 298 K. The MALDI MS experiments were carried out on a Bruker UltrafleXtreme MALDI TOF/TOF Mass Spectrometer (Bruker Daltonics, Billerica, MA), equipped with smartbeam-II laser. All spectra were measured in positive reflectron or linear mode. All the TEM measurements were performed under a Tecnai G2 20 TWIN device, the TEM samples were deposited on copper grids, followed by a slow evaporation in air at room temperature. The AFM samples were prepared by drop casting method using mica sheet as substrate. All the AFM images were obtained on a Dimension FastScan (Bruker), using ScanAsyst mode under ambient condition. UV−vis spectra were recorded in a quartz cell (light path 10 mm) on a Cary 50Bio UV-Visible spectrophotometer.

Section B. Synthesis and Characterizaiton of [2]rotaxane 2.

Supplementary Figure 1: The synthesis route of [2]rotaxane **2**.

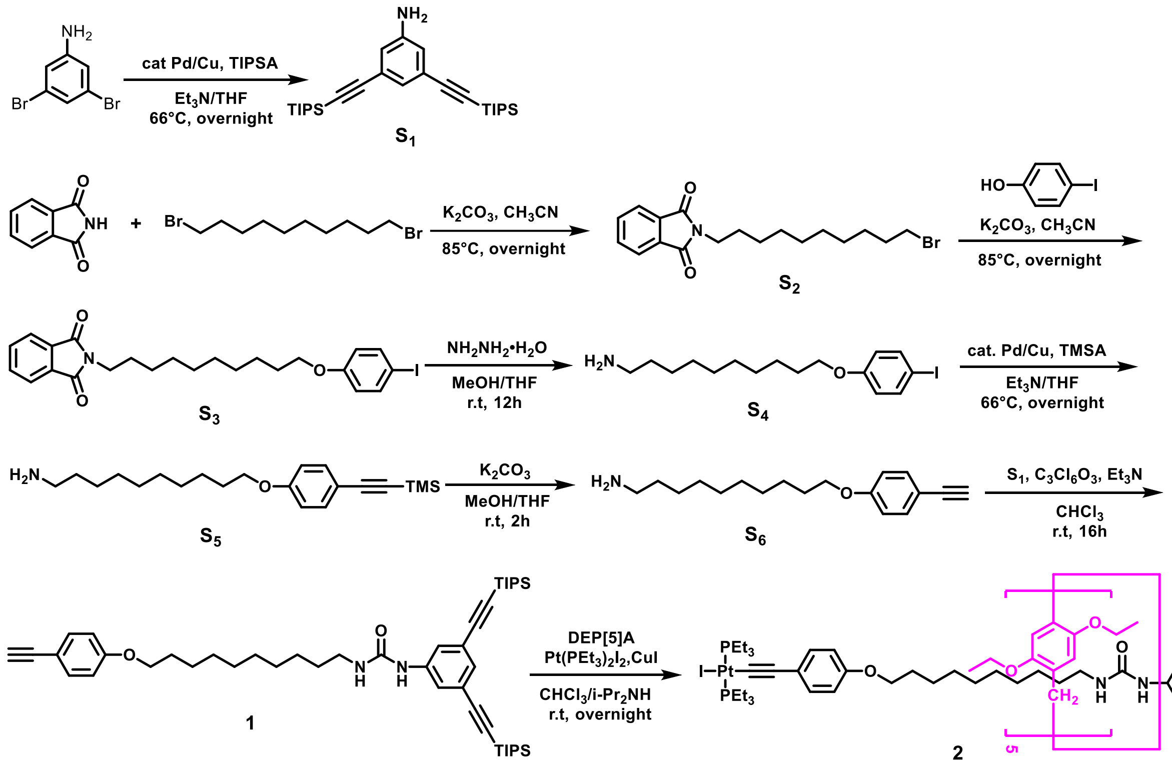

Synthesis of S3: Mixing compound **S2** (9.45 g, 25.88 mmol) and commercially available 4-iodophenol (5.70 g, 25.88 mmol) in acetonitrile (pre-dried by Na_2SO_4, 100 mL), then K_2CO_3 (14.28g, 103.52 mmol) was added. The resultant suspension was refluxed at 85 ℃ overnight. After cooling to room temperature, the reaction mixture was filtered and the filtrate was concentrated in vacuum to give a colorless oily residue, which was purified through column chromatography (SiO_2; PE/DCM) to yield **S3** (11.7 g, 90%) as a white solid. Mp: 65 ℃. ^{1}H NMR (300 MHz, $CDCl_3$): δ 7.82 -7.85 (m, 2H), δ 7.69 -7.71 (m, 2H), δ 7.51-7.54 (d, J = 9.0 Hz, 2H), δ 6.65-6.68 (d, J = 9.0 Hz, 2H), δ 3.87-3.92 (t, J = 7.5 Hz, 2H), δ 3.65-3.70 (t, J = 7.5 Hz, 2H), δ 1.65-1.77 (m, 12H), δ 1.29-1.42 (m, 4H). ^{13}C NMR (126 MHz, $CDCl_3$): δ 168.44, 158.96, 138.08, 133.80, 132.13, 123.11, 116.89, 82.33, 68.06, 38.02, 29.38, 29.32, 29.24, 29.08, 29.07, 28.54, 26.79, 25.90. HRMS (EI-TOF): Calculated for [**S3**]$^+$: 505.1114; Found: 505.1110.

Synthesis of S4: Compound **S3** (2 g, 3.96 mmol) was added in a Schlenk flask, after the Schlenk flask was evacuated and back-filled with N_2 three times, the mixture solvent of THF (50 mL) and MeOH (5 mL) were added via syringe, then hydrazine hydrate (2.4 mL) were added under an inert atmosphere. The reaction was heated to 65 ℃ and stirred for 12 h. After cooling to room temperature,

the reaction mixture was filtered and the filtrate was concentrated in vacuum. The obtained residue was washed by sodium hydroxide aqueous solution, then dried with Na_2SO_4 and concentrated. A white solid **S4** (1.37 g, 92%) was obtained. Mp: 70 ℃. ^{1}H NMR (400 MHz, $CDCl_3$): δ 7.52-7.54 (d, J = 8.0 Hz, 2H), 6.65-6.68 (d, J = 12.0 Hz, 2H), 3.89-3.92 (t, J = 6.0 Hz, 2H), 2.66-2.70 (t, J = 8.0 Hz, 2H), 1.74-1.77 (m, 2H), 1.29-1.43 (m, 14H). ^{13}C NMR (126 MHz, $CDCl_3$): δ 158.95, 138.08, 116.88, 82.34, 68.05, 42.14, 33.64, 29.48, 29.44, 29.41, 29.29, 29.08, 26.83, 25.92. HRMS (EI-TOF): Calculated for [**S4**]$^+$: 375.1059; Found: 375.1057.

Synthesis of S5: A Schlenk flask was charged with 1 g (2.70 mmol) of **S4**, CuI (50 mg, 0.27 mmol) and $Pd(PPh_3)_2Cl_2$ (189 mg, 0.27 mmol). The Schlenk flask was then evacuated and back-filled with N_2 three times. Anhydrous and degassed THF (20 mL) and Et_3N (10 mL) were added via syringe, then 2.5 equiv of trimethylsilylacetylene (0.9 mL) was added into the reaction flask. The reaction was heated to 66 ℃ and allowed to stir for overnight. After cooling to room temperature, the reaction mixture was filtered and the filtrate was concentrated in vacuum Then the residue was purified by column chromatography (SiO_2; PE/DCM) to yield a pale-yellow solid **S5** (0.57g, 60%). Mp: 91 ℃. ^{1}H NMR (300 MHz, $CDCl_3$): δ 7.37-7.40 (d, J = 9.0 Hz, 2H), δ 6.78-6.81 (d, J = 9.0 Hz, 2H), δ 3.91-3.95 (t, J = 6.0 Hz, 2H), δ 2.85-2.89 (t, J = 6.0 Hz, 2H), δ 1.73-1.78 (m, 2H), δ 1.30-1.64 (m, 14H), δ 0.23 (s, 9H). ^{13}C NMR (101 MHz, $CDCl_3$): δ 159.28, 133.40, 114.91, 114.27, 105.26, 92.25, 67.95, 40.96, 30.37, 29.43, 29.38, 29.31, 29.14, 26.66, 25.97, 0.05. HRMS (EI-TOF): Calculated for [**S5**]$^+$: 345.2488; Found: 345.2489.

Synthesis of S6: A 10 mL THF/MeOH solution (v/v, 1:1) of **S5** was added into the round-bottom flask, then K_2CO_3 (1.5 g) was added into the resultant solution. The mixture was allowed to stir for 2 h at room temperature. After filtering the insoluble inorganic salt, the solvent was evaporated in vacuum and the residue was purified by column chromatography (SiO_2; DCM/MeOH) to yield **S6** (150 mg, 76%) as a pale-yellow solid. Mp: 61 ℃. ^{1}H NMR (300 MHz, $CDCl_3$): δ 7.40-7.42 (d, J = 6.0 Hz, 2H), δ 6.81-6.84 (d, J = 9.0 Hz, 2H), δ 3.92-3.97 (t, J = 7.5 Hz, 2H), δ 2.99 (s, 1H), δ 2.71-2.75 (t, J = 6.0 Hz, 2H), δ 2.49 (s, 2H), δ 1.74-1.79 (m, 2H), δ 1.30-1.51 (m, 14H). ^{13}C NMR (101 MHz, $CDCl_3$): δ 159.48, 133.52, 114.39, 113.80, 83.72, 75.63, 67.99, 41.98, 33.27, 29.49, 29.45, 29.40, 29.31, 29.11, 26.83, 25.95. HRMS (EI-TOF): Calculated for [**S6**]$^+$: 273.2093; Found: 273.2090.

Synthesis of 1: A $CHCl_3$ (15 mL) solution of triphosgene (178 mg, 0.61 mmol) was added into a Schlenk flask, the Schlenk flask was evacuated and back-filled with N_2 three times. Then a $CHCl_3$ (15 mL) solution of **S_1** (824 mg, 1.82 mmol) and triethylamine (1 mL) were added into the reaction flask and stirred at 0 ℃. After 5 h, adding the $CHCl_3$ (15 mL) solution of **S_6** (500 mg, 1.83 mmol) into reaction flask, then the reaction mixture was allowed to warm to room temperature and stirred overnight. The solution was concentrated and the residue was purified through chromatography (SiO_2; PE/DCM) to afford compound **1** as a pale-yellow solid (650 mg, 47%). Mp: 108 ℃. ^{1}H NMR (400 MHz, acetone-d_6): δ 8.03 (s, 1H), δ 7.68 (d, 2H), δ 7.38-7.42 (m, 2H), δ 7.12-7.13 (t, J = 2.0 Hz, 1H), δ 6.90-6.92 (m, 2H), δ 5.84-5.87 (t, J = 6.0 Hz, 1H), δ 3.99-4.03 (t, J = 8.0 Hz, 2H), δ 3.47 (s, 1H), δ 3.18-3.23 (m, 2H), δ 1.73-1.80 (m, 2H), δ 1.29-1.53 (m, 16H), δ 1.14 (s, 42H). ^{13}C NMR (126 MHz, acetone-d_6): δ 160.55, 156.00, 142.21, 134.23, 128.48, 124.77, 122.21, 115.45, 115.00, 107.45, 91.02, 84.34, 77.50, 68.72, 40.58, 31.03, 30.36, 30.33, 30.15, 29.96, 27.67, 26.77, 19.09, 12.04. HRMS (ESI-TOF): Calculated for [**1** + H]$^+$: m/z = 753.5132; Found: m/z = 753.5305.

Synthesis of 2: A Schlenk flask was charged with 150 mg (0.20 mmol) of **1**, DEPillar[5]arene (1.07 g, 1.20 mmol) and $Pt(PEt_3)_2I_2$ (548 mg, 0.80 mmol). The Schlenk flask was then evacuated and back-filled with N_2 three times. Next, 5 mL of the mixture solvent of anhydrous and degassed $CHCl_3$ and i-Pr_2NH (v/v, 2:1) was added via syringe. The resultant solution was stirred for two hours under -10 ℃. Then CuI (4 mg) was added to the mixture under an inert atmosphere and the mixture was allowed to warm to room temperature and stirred overnight. The solution was concentrated and the residue was purified by column chromatography (SiO_2; PE/DCM). A white solid **2** (308 mg, 70%) was obtained. Mp: 111 ℃. ^{1}H NMR (300 MHz, THF-d_8): δ 7.64-7.65 (d, J = 3.0 Hz, 2H), δ 7.60 (s, 1H), δ 7.20-7.23 (d, J = 9.0 Hz, 2H), δ 7.10 (s, 1H), δ 6.92-6.93 (d, J = 3.0 Hz, 10H), δ 6.74-6.77 (d, J = 9.0 Hz, 2H), δ 4.80 (t, 1H), δ 3.82-4.07 (m, 22H), δ 3.72 (s, 10H), δ 2.96-3.02 (m, 2H), δ 2.22-2.31 (m, 12H), δ 1.45-1.50 (t , J = 7.5 Hz, 15H), δ 1.36-1.41 (t , J = 7.5 Hz, 15H), δ 1.14-1.22 (m, 60H), δ 0.79 (s, 2H), δ 0.16 (s, 2H), δ 0.03 (s, 2H), δ -0.71 (s, 2H), δ -1.18 (s, 2H). ^{31}P NMR (122 MHz, THF-d_8): δ 9.55. ^{13}C NMR (126 MHz, THF-d_8): δ 158.73, 155.32, 150.78, 150.73, 142.46, 132.35, 129.22, 128.58, 124.77, 122.30, 115.01, 114.97, 114.83, 107.98, 90.73, 69.52, 64.40, 64.38, 40.67, 31.89, 31.81, 31.65, 31.18, 30.18, 30.02, 29.73, 28.32, 19.24, 17.83, 17.69, 17.55, 16.04, 16.00, 12.41, 8.77. HRMS (MALDI-TOF-MS): Calculated for [**2** + H]$^+$: m/z = 2201.7300; Found: m/z = 2201.0498.

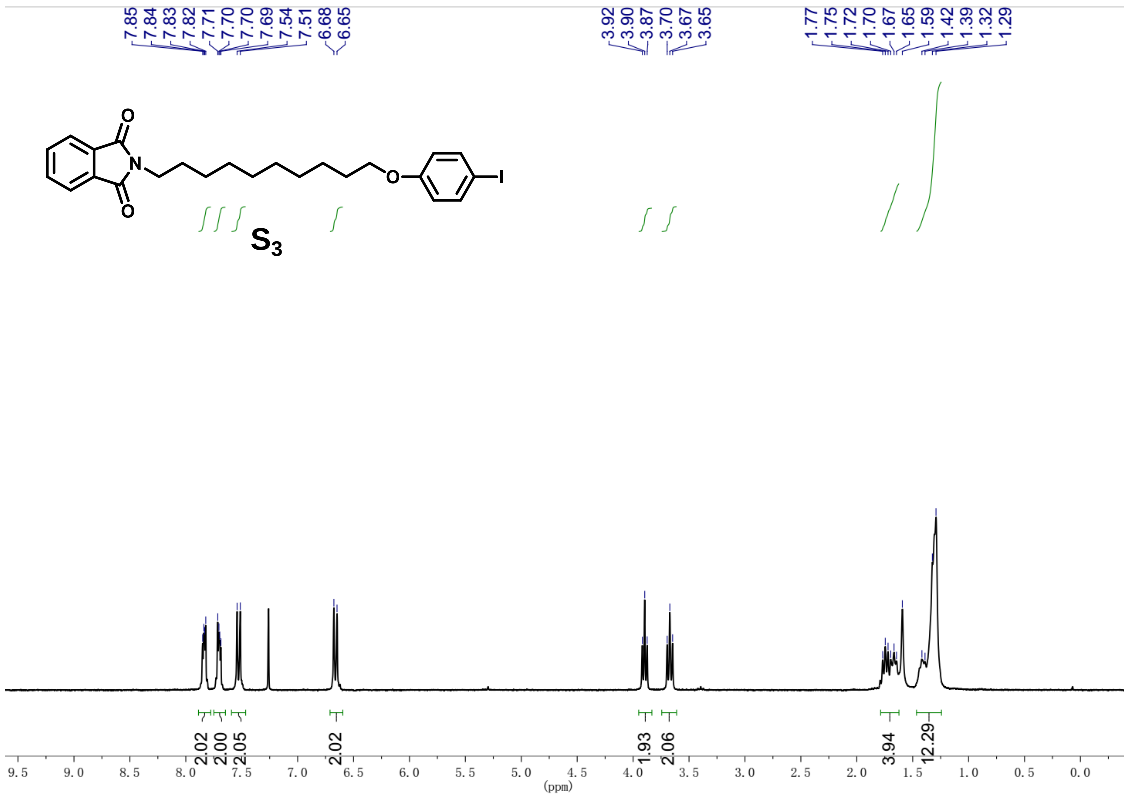

Supplementary Figure 2: ^{1}H NMR spectrum ($CDCl_3$, 298 K, 300 MHz) of compound **S_3**.

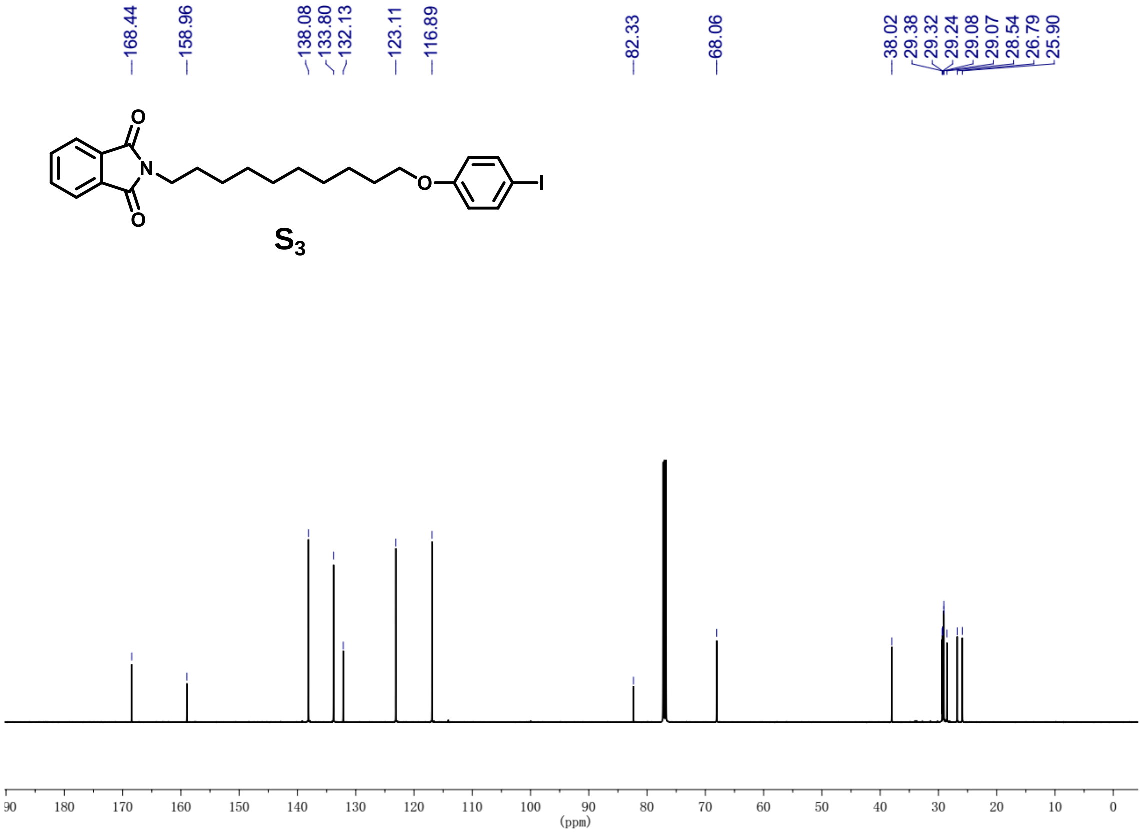

Supplementary Figure 3: ^{13}C NMR spectrum ($CDCl_3$, 298 K, 126 MHz) of compound **S_3**.

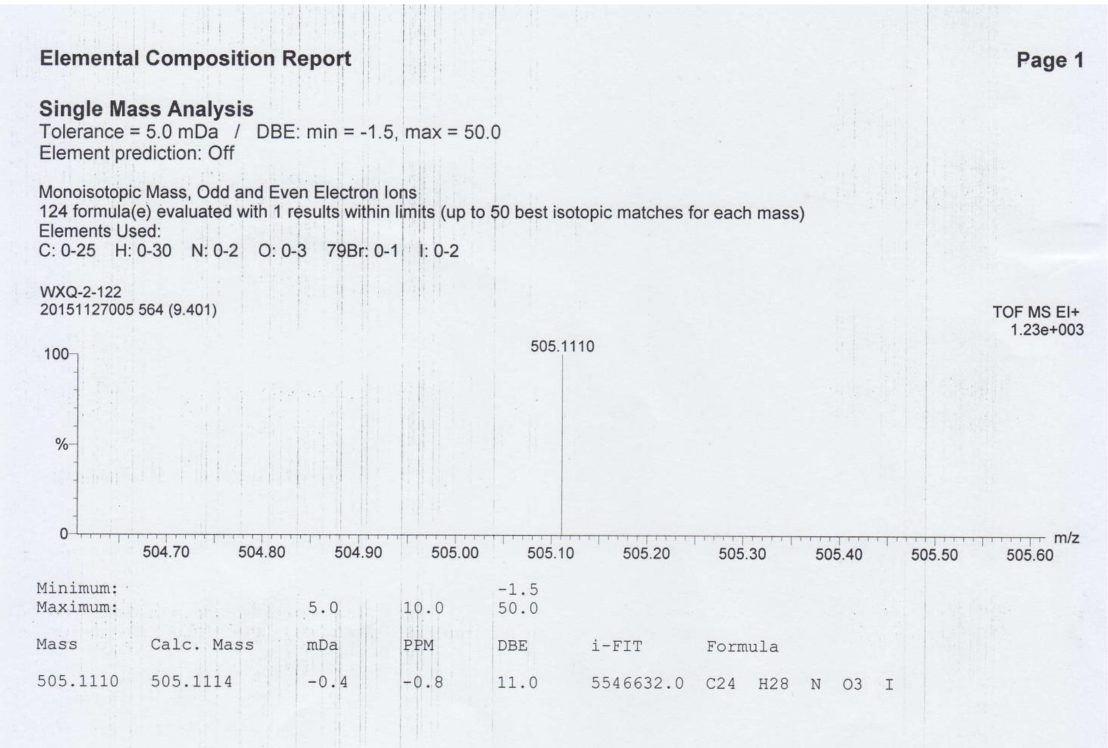

Supplementary Figure 4: HRMS (EI-TOF) spectrum of compound **S**$_{3}$.

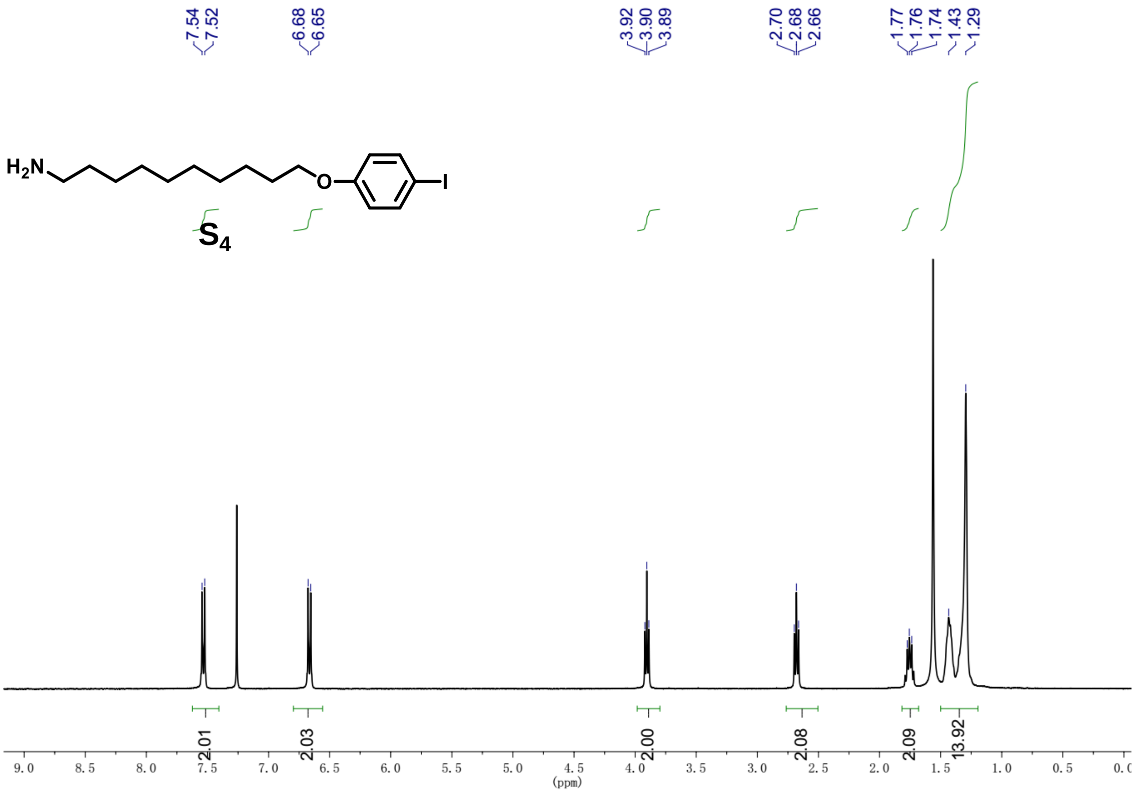

Supplementary Figure 5: ^{1}H NMR spectrum ($CDCl_3$, 298 K, 400 MHz) of compound **S**$_{4}$.

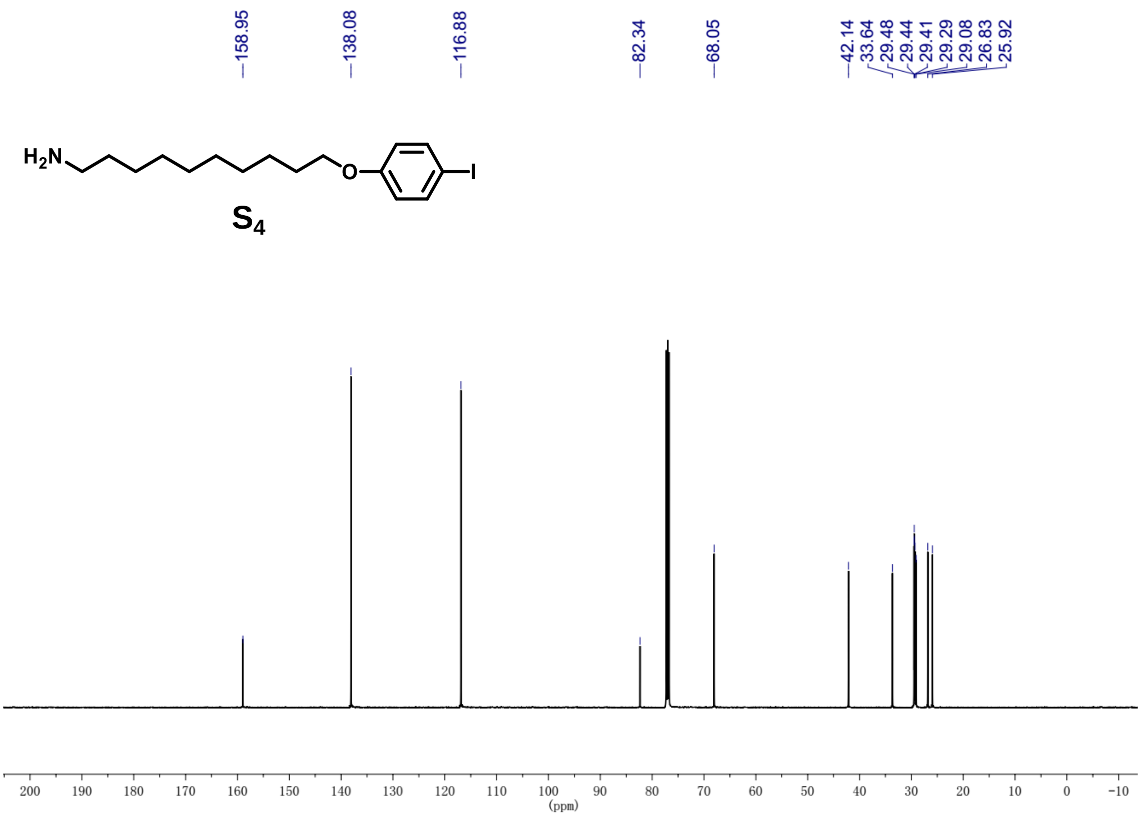

Supplementary Figure 6: ^{13}C NMR spectrum ($CDCl_3$, 298 K, 126 MHz) of compound **S_4**.

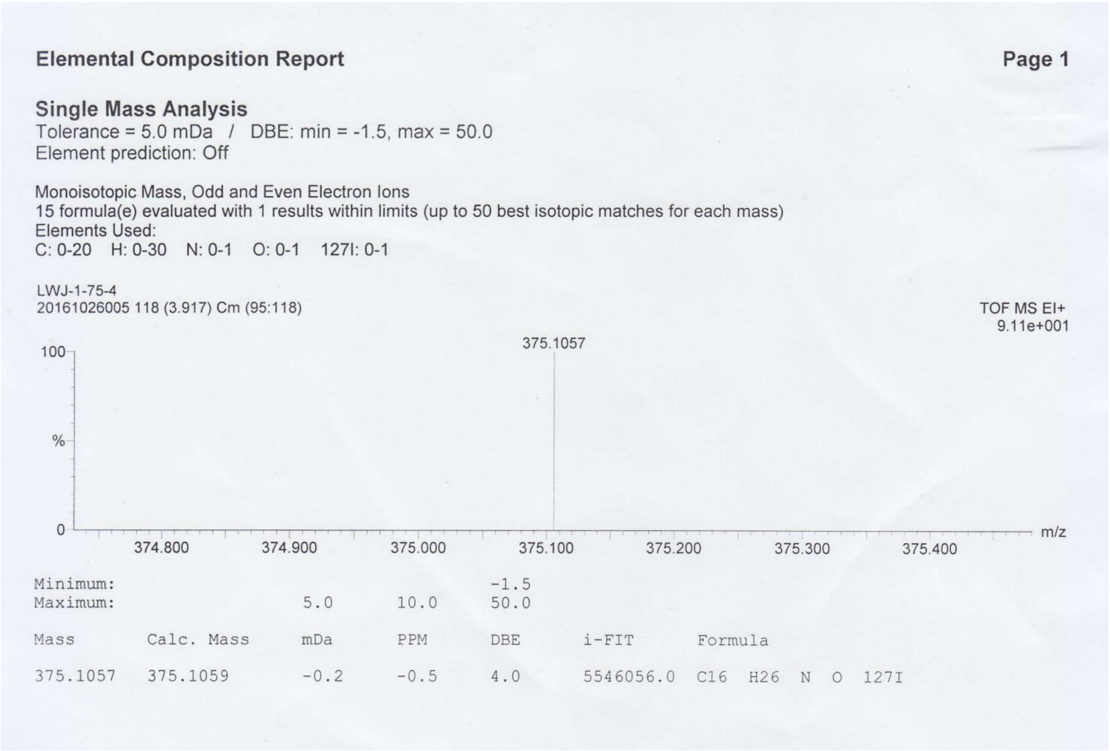

Supplementary Figure 7: HRMS (EI-TOF) spectrum of compound **S_4**.

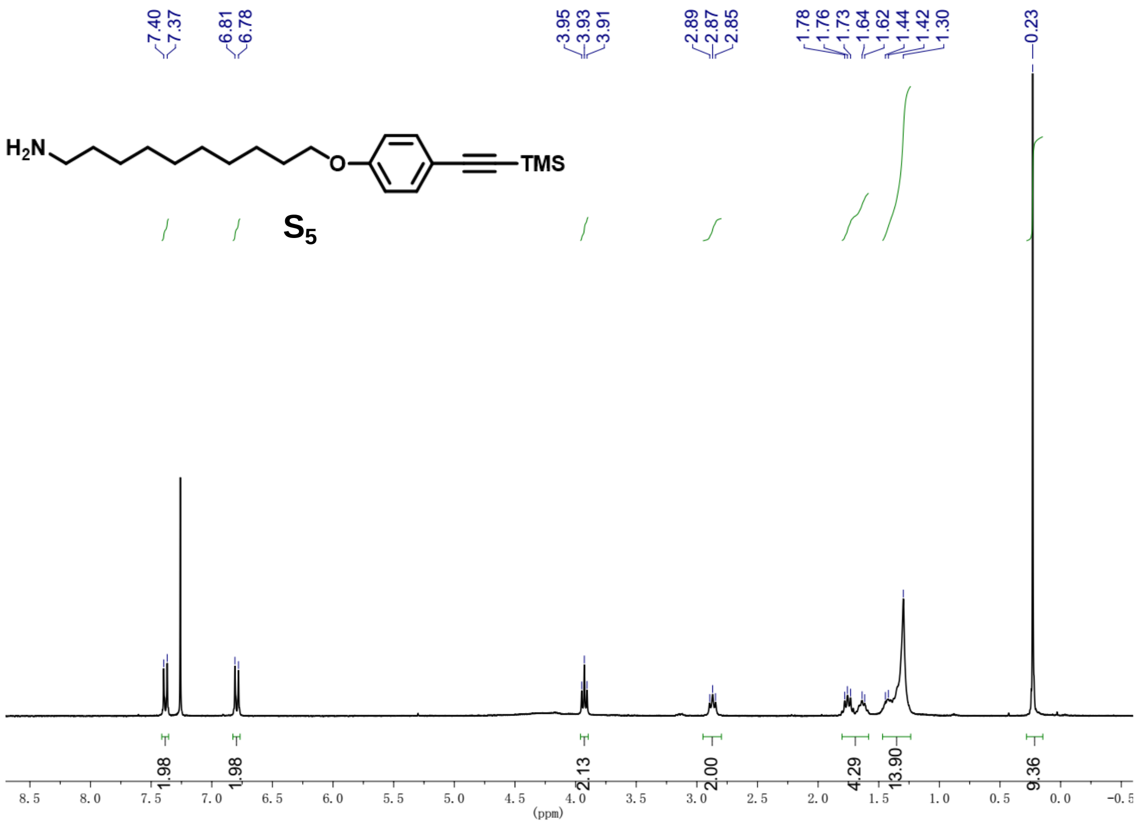

Supplementary Figure 8: ^{1}H NMR spectrum ($CDCl_3$, 298 K, 300 MHz) of compound **S_5**.

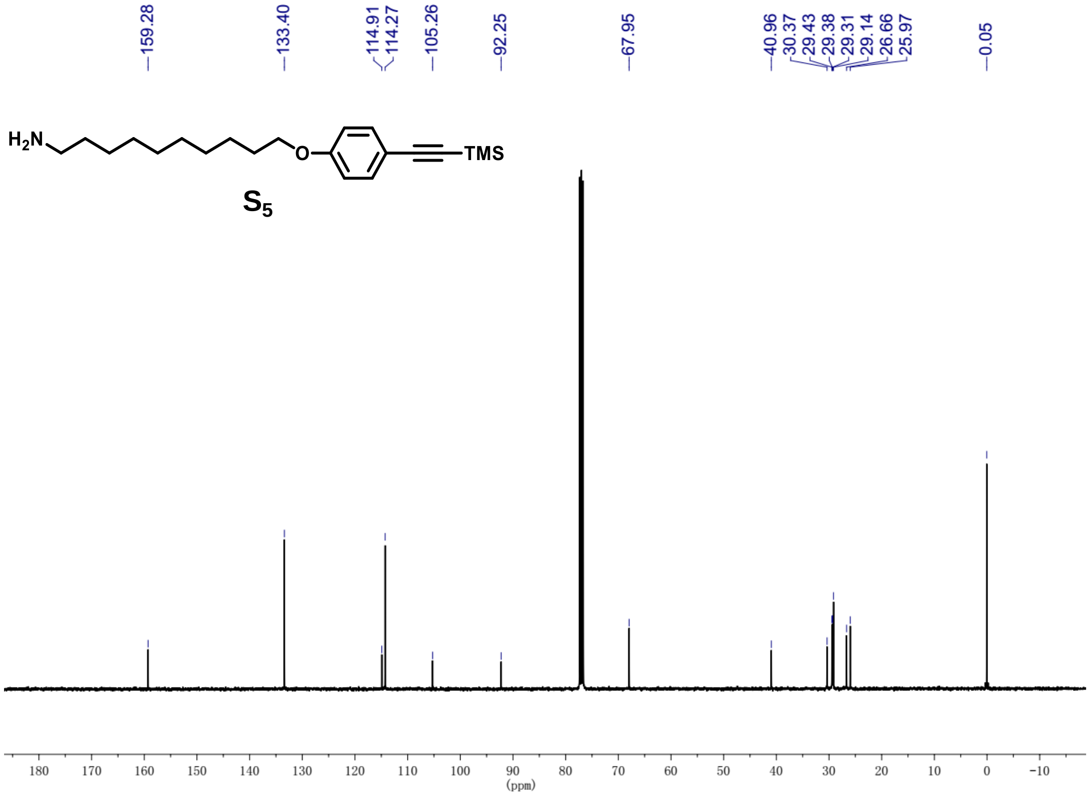

Supplementary Figure 9: ^{13}C NMR spectrum ($CDCl_3$, 298 K, 101 MHz) of compound **S_5**.

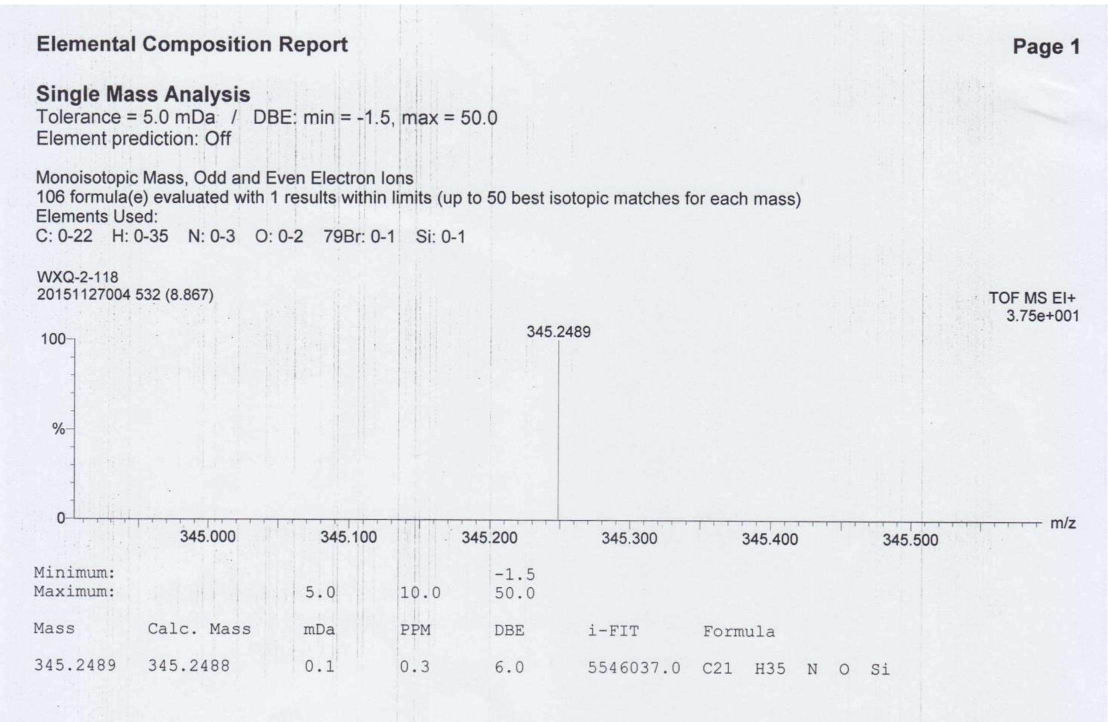

Supplementary Figure 10: HRMS (EI-TOF) spectrum of compound **S5**.

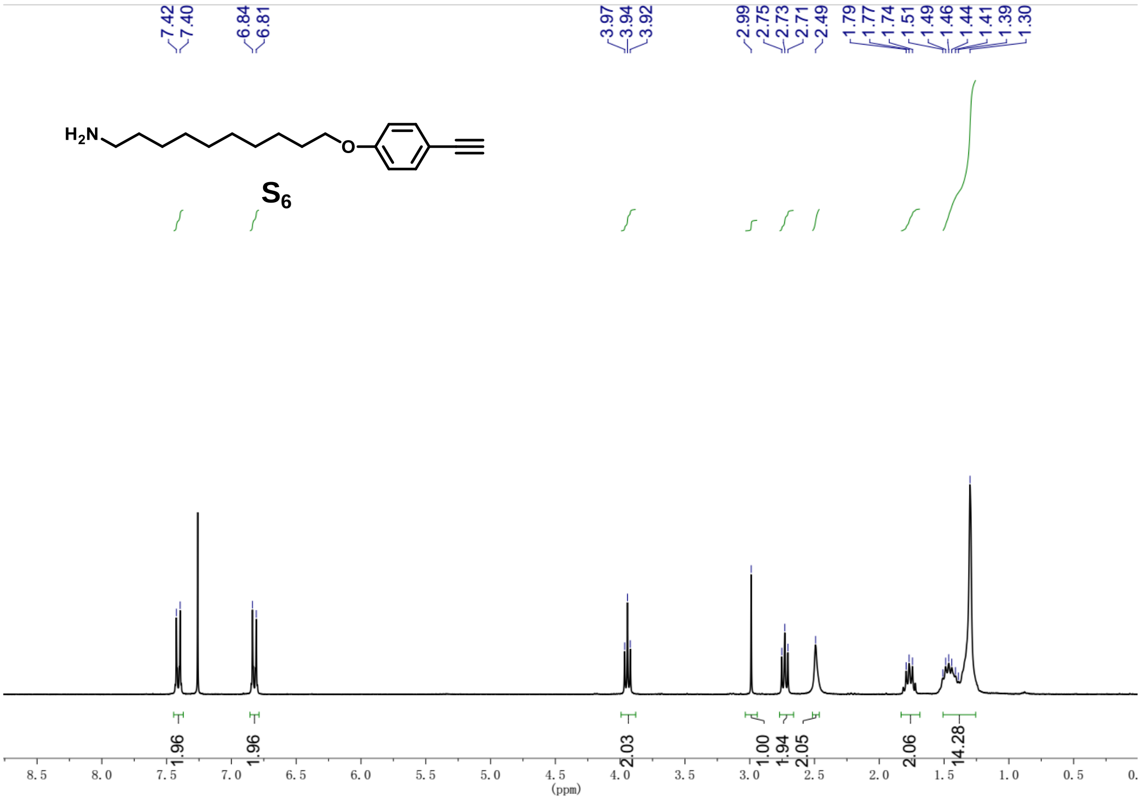

Supplementary Figure 11: ^{1}H NMR spectrum ($CDCl_3$, 298 K, 300 MHz) of compound **S6**.

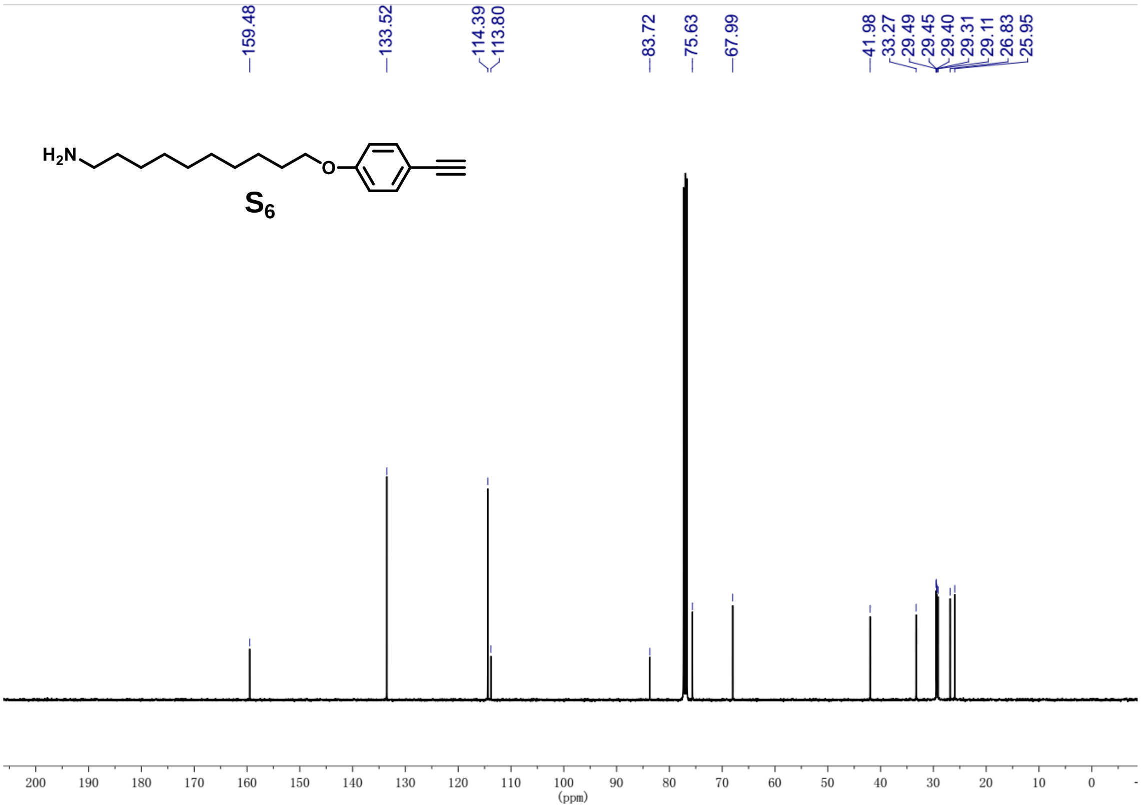

Supplementary Figure 12: ^{13}C NMR spectrum ($CDCl_3$, 298 K, 101 MHz) of compound **S$_6$**.

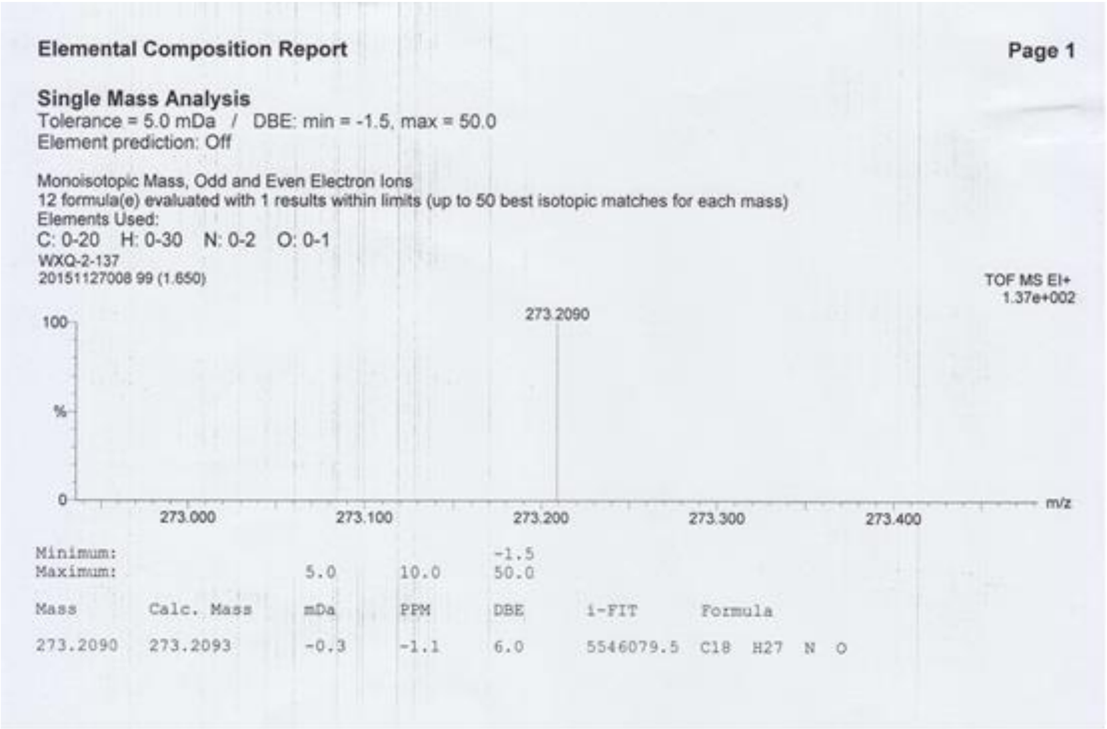

Supplementary Figure 13: HRMS (EI-TOF) spectrum of compound **S$_6$**.

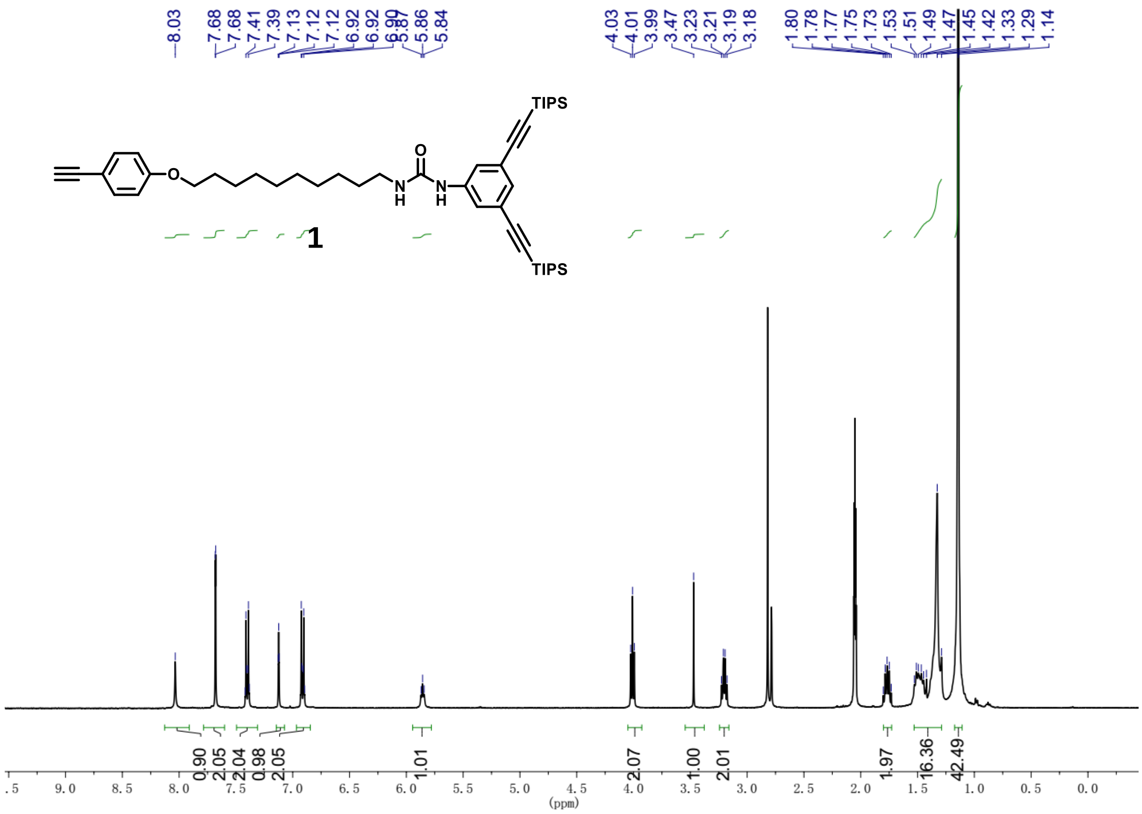

Supplementary Figure 14: ^{1}H NMR spectrum (acetone-d_6, 298 K, 400 MHz) of compound **1**.

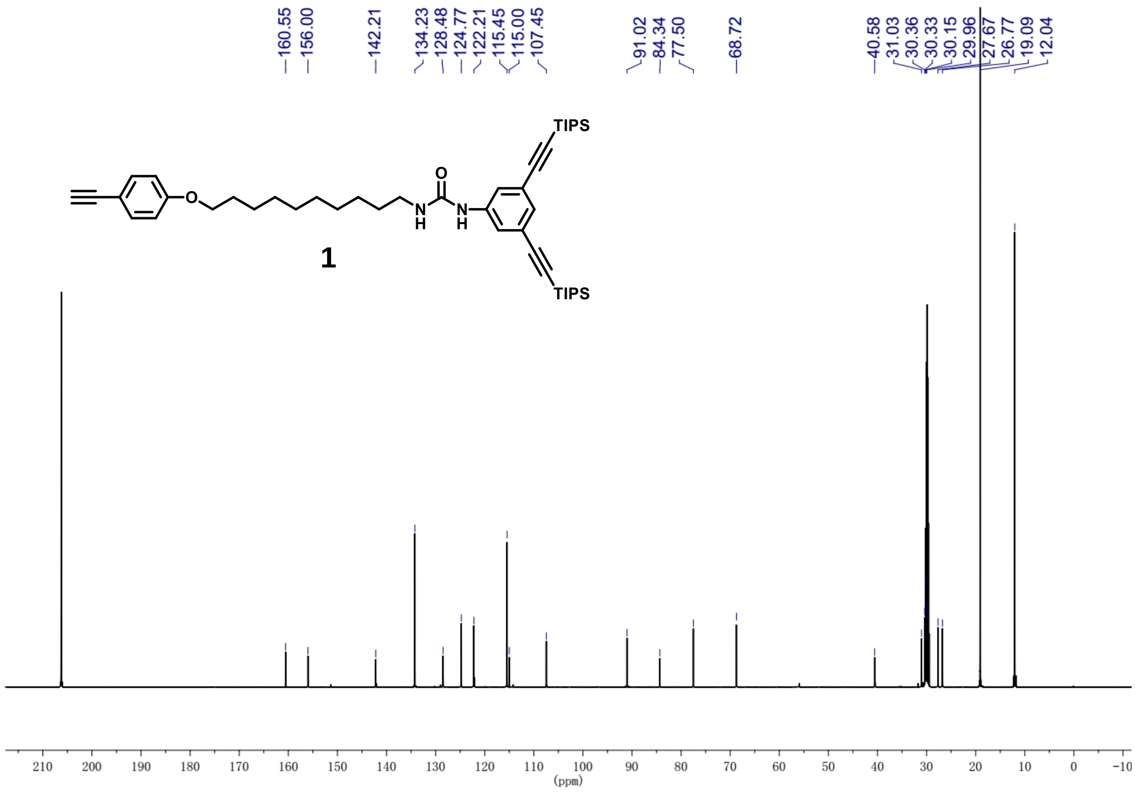

Supplementary Figure 15: ^{13}C NMR spectrum (acetone-d_6, 298 K, 126 MHz) of compound **1**.

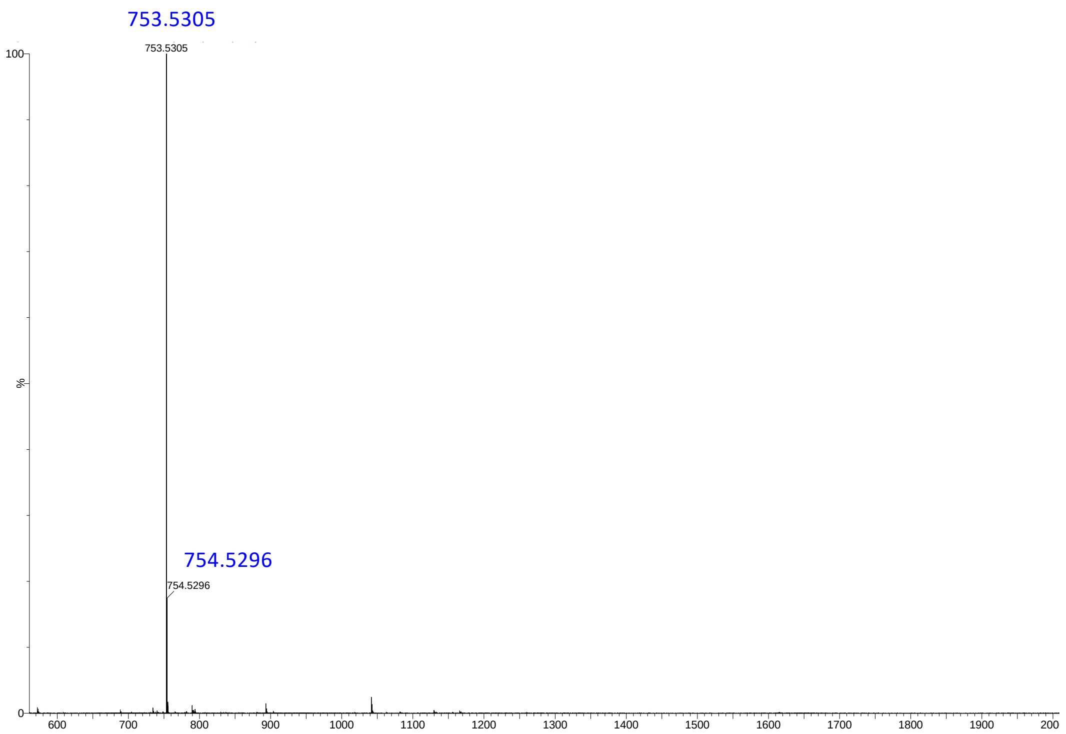

Supplementary Figure 16: HRMS (ESI-TOF) spectrum of compound **1**.

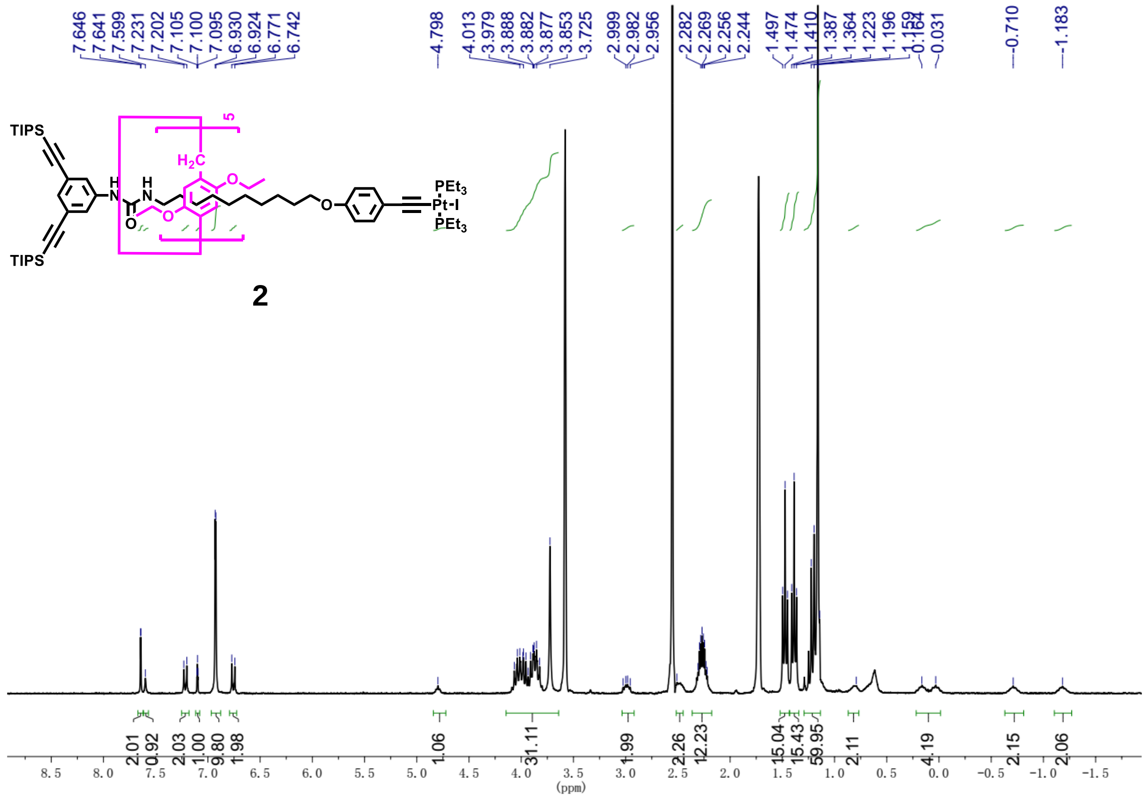

Supplementary Figure 17: ^{1}H NMR spectrum (THF-d_8, 298 K, 300 MHz) of [2]rotaxane **2**.

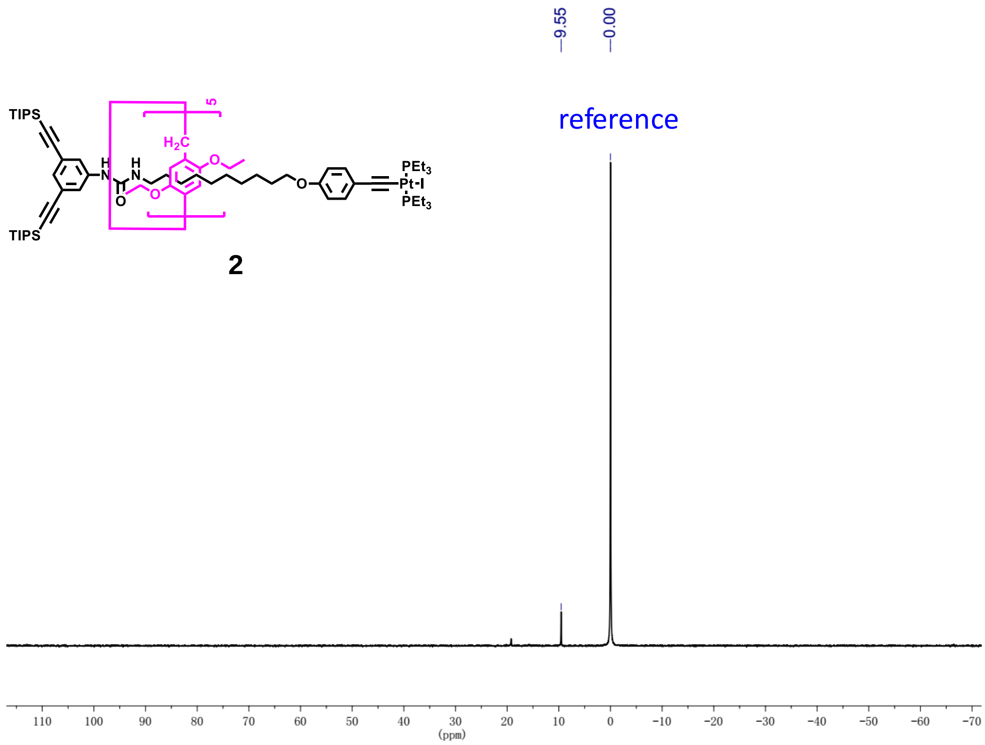

Supplementary Figure 18: ^{31}P NMR spectrum (THF-d_8, 298 K, 122 MHz) of [2]rotaxane **2**.

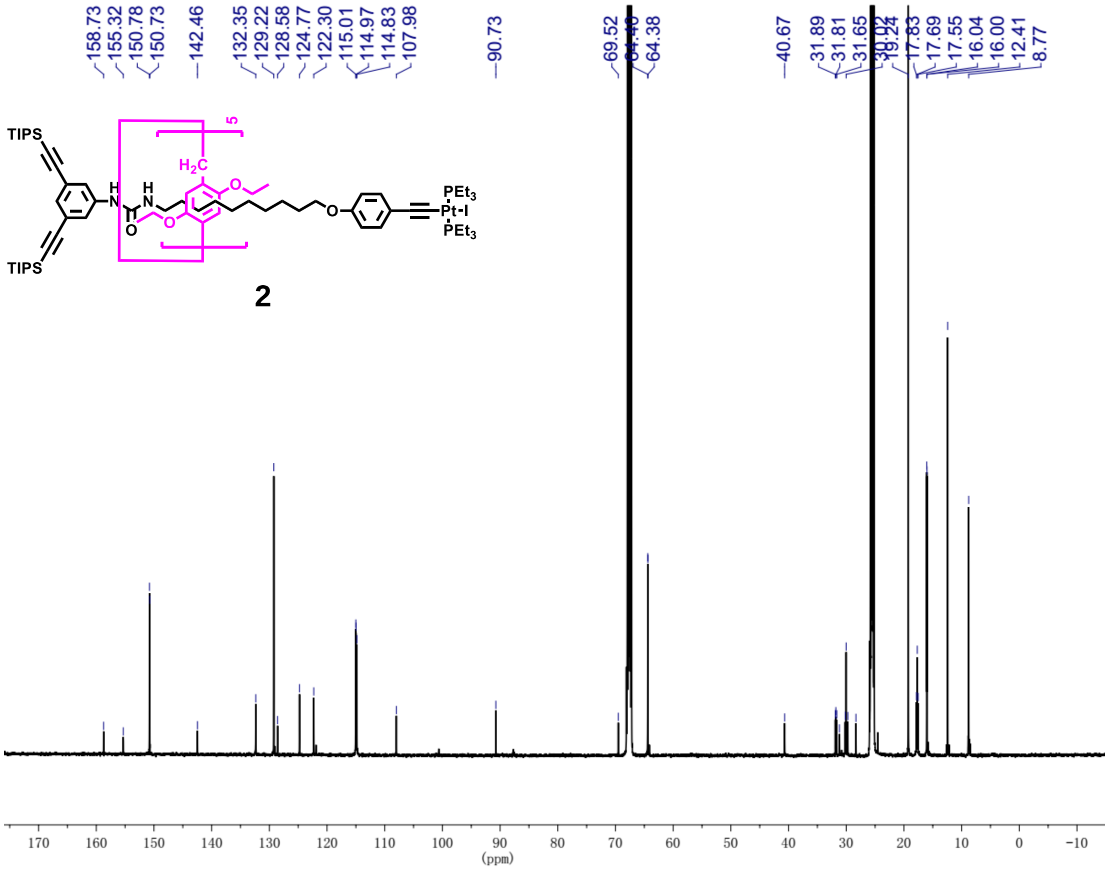

Supplementary Figure 19: ^{13}C NMR spectrum (THF-d_8, 298 K, 126 MHz) of [2]rotaxane **2**.

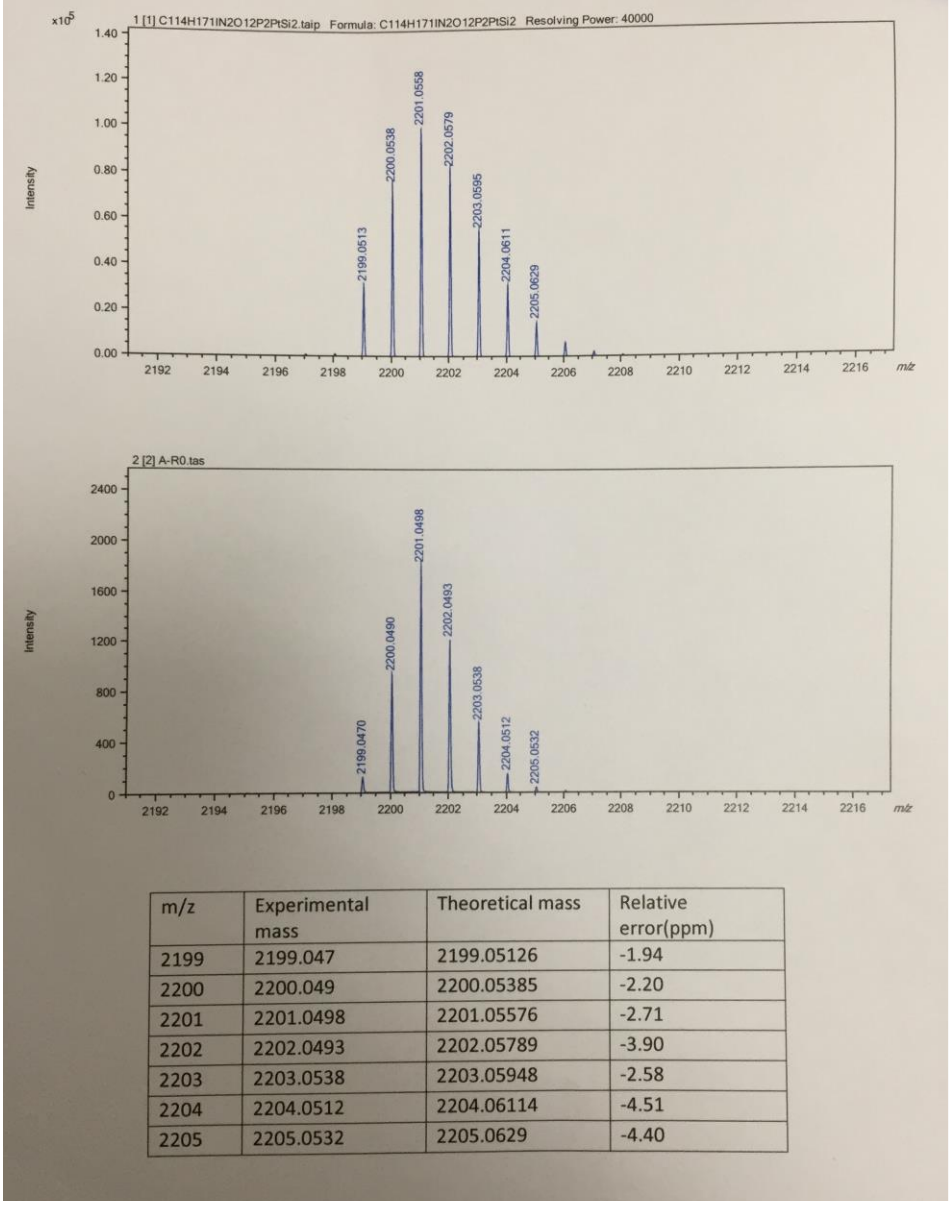

m/z	Experimental mass	Theoretical mass	Relative error(ppm)
2199	2199.047	2199.05126	-1.94
2200	2200.049	2200.05385	-2.20
2201	2201.0498	2201.05576	-2.71
2202	2202.0493	2202.05789	-3.90
2203	2203.0538	2203.05948	-2.58
2204	2204.0512	2204.06114	-4.51
2205	2205.0532	2205.0629	-4.40

Supplementary Figure 20: MALDI-TOF-MS spectrum of [2]rotaxane **2**.

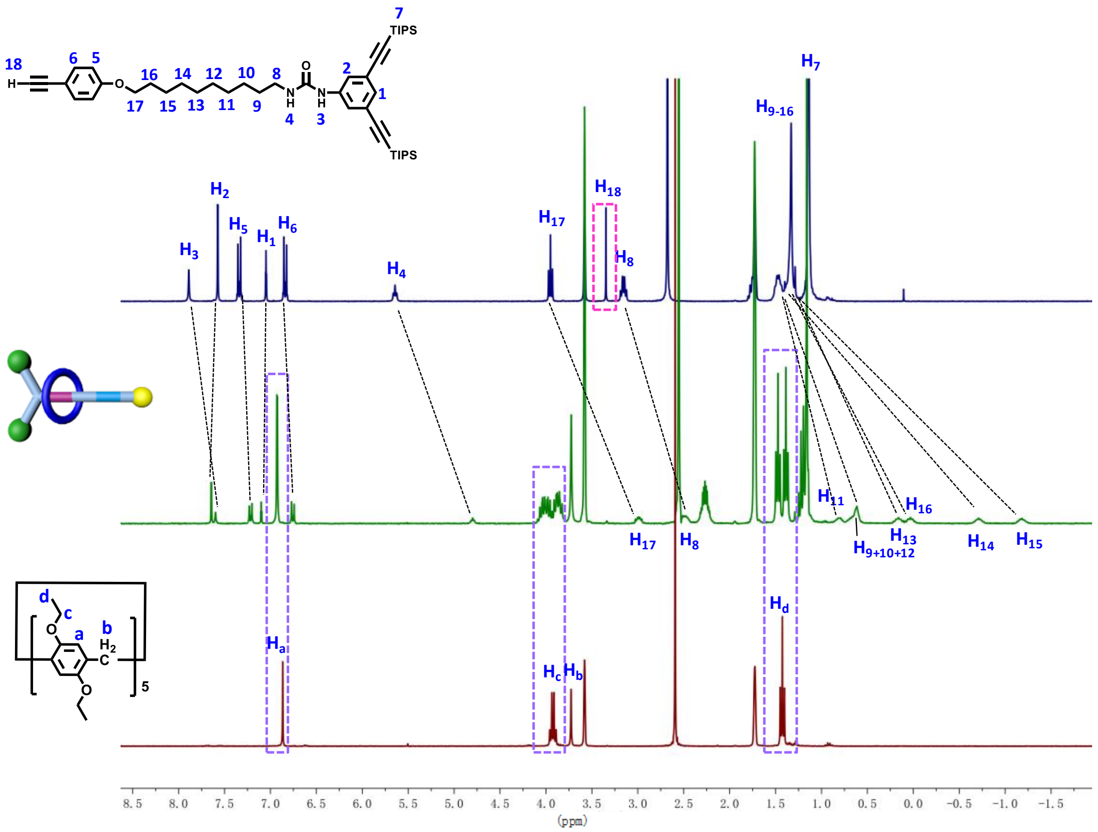

Supplementary Figure 21: ^{1}H NMR spectra (THF-d_8, 298 K, 300 MHz) of DEP5 macrocycle (*bottom*); [2]rotaxane **2** (*middle*); semi-blocked rod-like component **1** (*top*). (The signal of acetylenic hydrogen (H_{18}) on the component **1** disappeared, which indicated the complete conversion of the terminal acetylene in the coupling reaction. The signal of the protons H_a, H_c, and H_d ascribed to DEP5 macrocycle were split into two peaks, respectively, suggesting that the DEP5 ring was located at the axle component with different stoppers.)

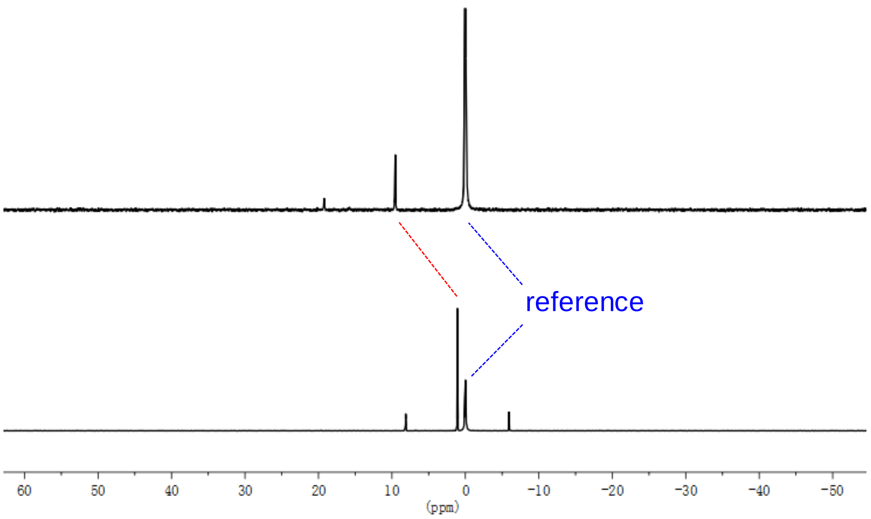

Supplementary Figure 22: ^{31}P NMR spectra (THF-d_8, 298 K, 122 MHz) of [2]rotaxane **2** (*top*) and $Pt(PEt_3)_2I_2$ (*bottom*).

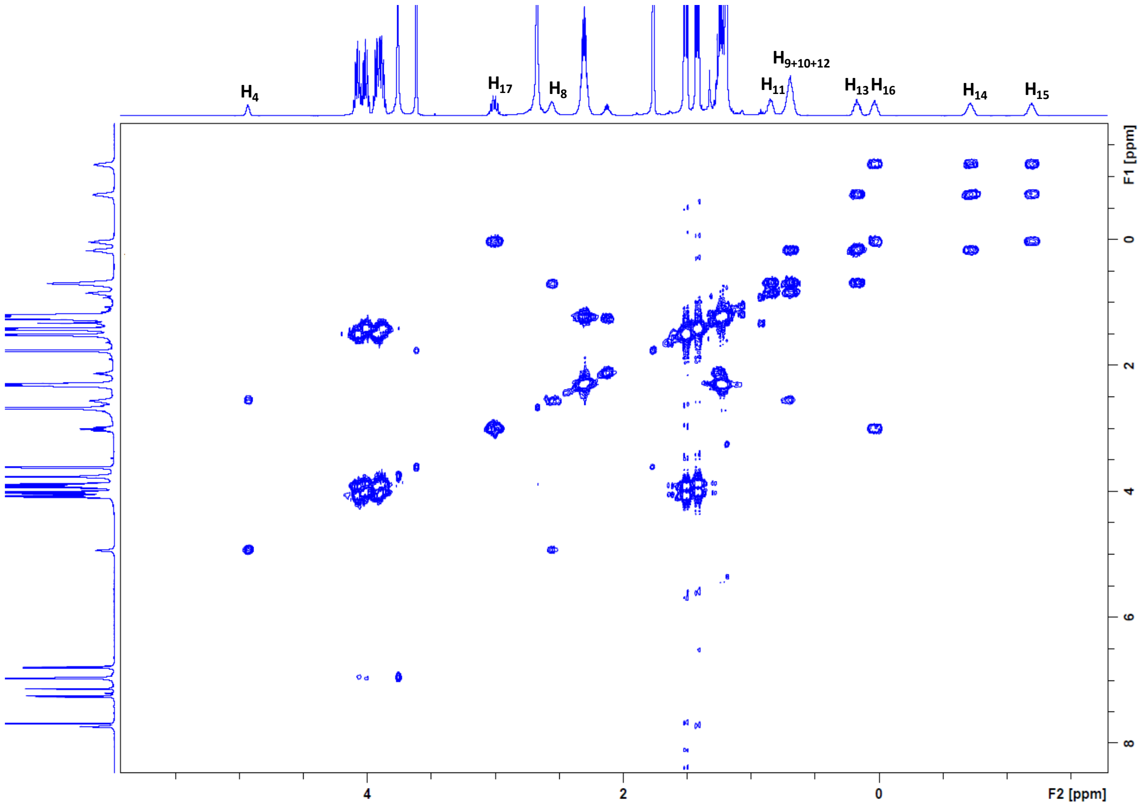

Supplementary Figure 23: 2D ^{1}H-^{1}H COSY spectrum (THF-d_8, 298 K, 500 MHz) of [2]rotaxane **2**.

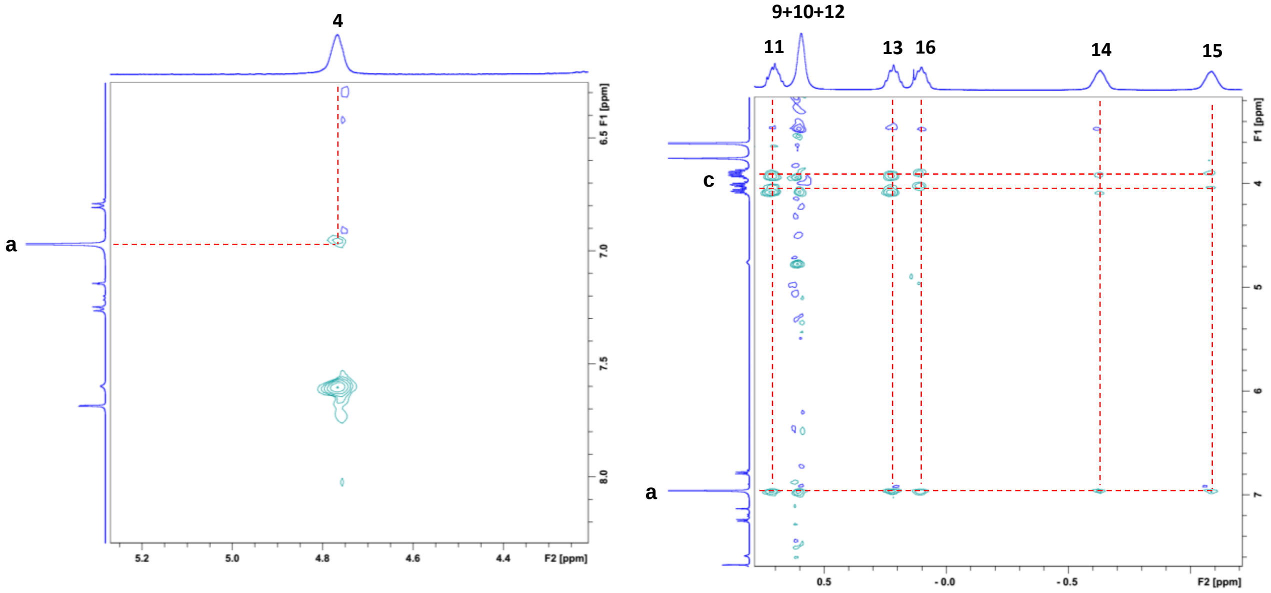

Supplementary Figure 24: 2D ^{1}H-^{1}H ROESY spectrum (THF-d_8, 298 K, 500 MHz) of [2]rotaxane **2**.

Section C. Synthesis and characterizaiton of the rotaxane-branched dendrimers.

General procedure for the synthesis of rotaxane-branched dendrimer $\mathbf{G_n}$: A mixture of multiyne complexes (for $\mathbf{G_1}$, 1, 3, 5-triethynylbenzene; for $\mathbf{G_2}$, $\mathbf{G_1}$**-YNE**; for $\mathbf{G_3}$, $\mathbf{G_2}$**-YNE**) and **2** (for each terminal acetylene moiety, 1.1 equiv [2]rotaxane **2** was added) in degassed diethylamine was stirred overnight at room temperature in the presence of a catalytic amount of CuI. The solvent was evaporated and the residue was purified by column chromatography and gel permeation chromatography (GPC) to afford $\mathbf{G_n}$ as pale-yellow solid.

General procedure for the synthesis of the deprotected rotaxane-branched dendrimer $\mathbf{G_n}$-YNE: To a solution of $\mathbf{G_n}$ in THF, a solution of Bu_4NF in THF (1.0 M) was added dropwise. The reaction mixture was stirred at room temperature for 4h. The obtained residue was washed by water, then dried over Na_2SO_4 and concentrated. The residue was further purified by column chromatography and gel permeation chromatography (GPC) to afford $\mathbf{G_n}$**-YNE** as pale-yellow solid.

Supplementary Figure 25: Synthesis of the first-generation rotaxane-branched dendrimer $\mathbf{G_1}$.

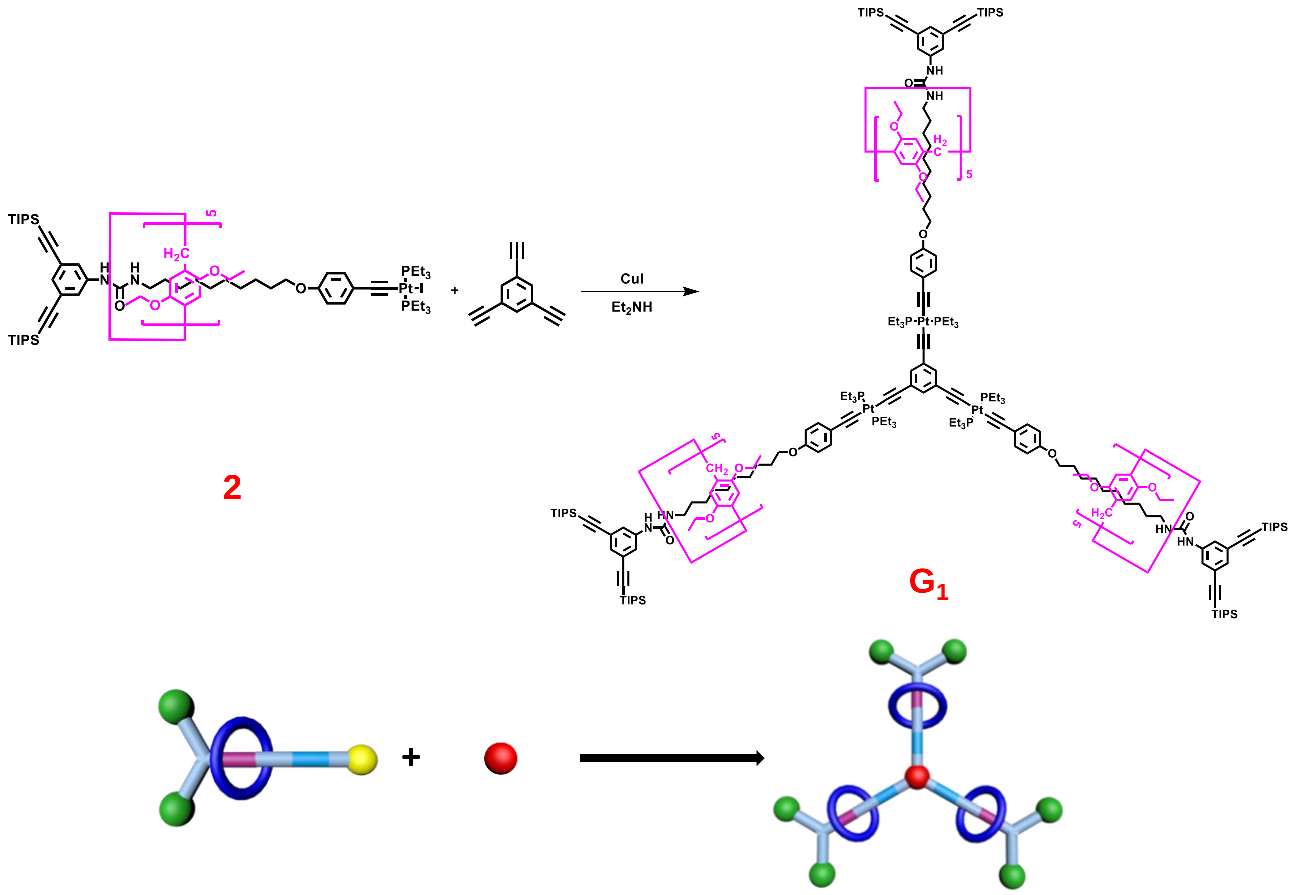

Synthesis of the first-generation rotaxane-branched dendrimer G₁: A mixture of 1, 3, 5-triethynylbenzene (3.2 mg, 0.021 mmol) and [2]rotaxane monomer **2** (150 mg, 0.068 mmol) were added in a Schlenk flask, the Schlenk flask was then evacuated and back-filled with N_2 three times. Next, degassed diethylamine (8.0 mL) and a catalytic amount of CuI were added under an inert atmosphere. The reaction was stirred overnight at room temperature. The solvent was evaporated and the residue was purified by column chromatography (SiO_2; PE/DCM) and gel permeation chromatography (GPC) to yield a pale-yellow solid **G_1** (99 mg, 74%). ^{1}H NMR (400 MHz, THF-d_8): δ 7.64 (s, 6H), δ 7.55 (s, 3H), δ 7.18-7.20 (d, J = 6.0 Hz, 6H), δ 7.10 (s, 3H), δ 6.93-6.94 (d, J = 3.0 Hz, 30H), δ 6.89 (s, 3H), δ 6.73-6.75 (d, J = 6.0 Hz, 6H), δ 4.75 (s, 3H), δ 3.86-4.06 (m, 66H), δ 3.73 (s, 30H), δ 3.00-3.02 (m, 6H), 2.45 (s, 6H), 2.22-2.24 (m, 36H), 1.38-1.49 (m, 90H), 1.26-1.32 (m, 54H), 1.16 (s, 126H), 0.81 (s, 6H), 0.59-0.67 (m, 12H), 0.17 (s, 6H), -0.06 (s, 6H), -0.70 (s, 6H), -1.16 (s, 6H), ^{31}P NMR (122 MHz, THF-d_8): δ 12.44. ^{13}C NMR (101 MHz, THF-d_8): δ 157.94, 154.87, 150.44, 150.35, 146.46, 142.07, 136.55, 132.11, 128.87, 128.84, 128.22, 124.42, 121.97, 120.39, 114.63, 114.29, 113.43, 107.61, 94.93, 90.39, 88.77, 69.08, 67.94, 64.06, 64.00, 40.31, 31.54, 31.47, 31.30, 30.73, 29.85, 29.67, 27.93, 25.66, 25.53, 25.46, 25.33, 25.26, 25.13, 18.89, 17.32, 17.15, 16.97, 15.69, 15.66, 12.06, 8.59. HRMS (ESI-MS): Calculated for [**G_1** + 3H]$^{3+}$: m/z = 2124.8766; Found: m/z = 2124.7927. Calculated for [**G_1** + 4H]$^{4+}$: m/z = 1593.9075; Found: m/z = 1593.9254.

Supplementary Figure 26: Synthesis of the second-generation rotaxane-branched dendrimer **G_2**.

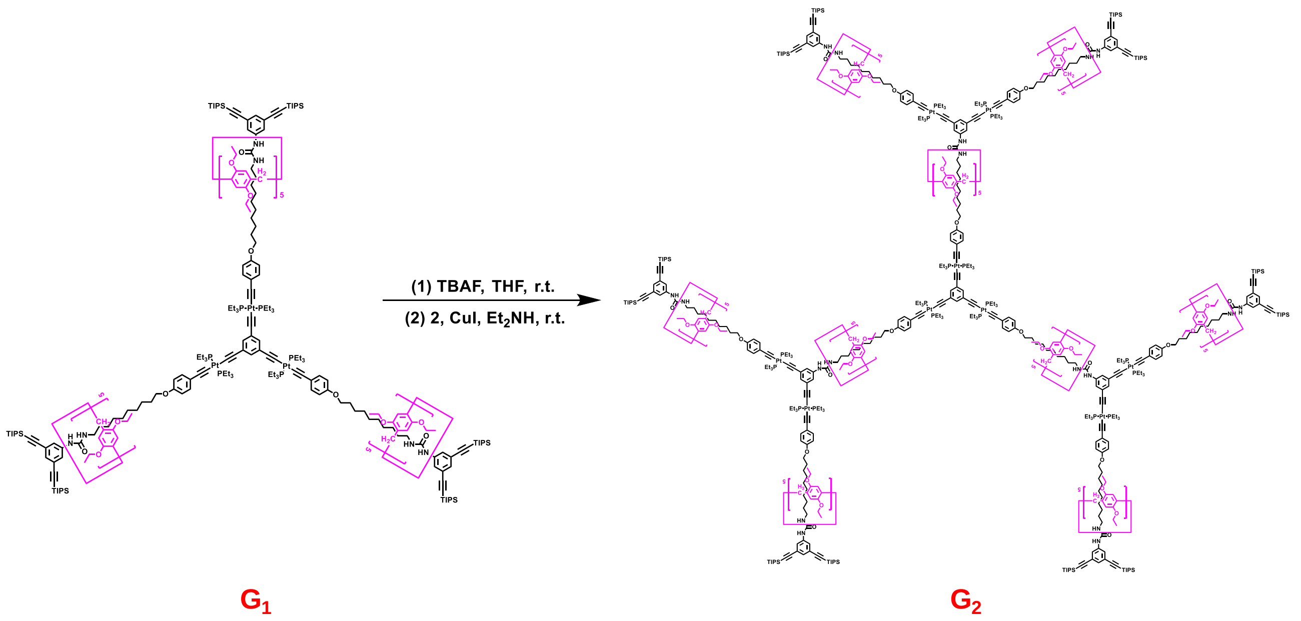

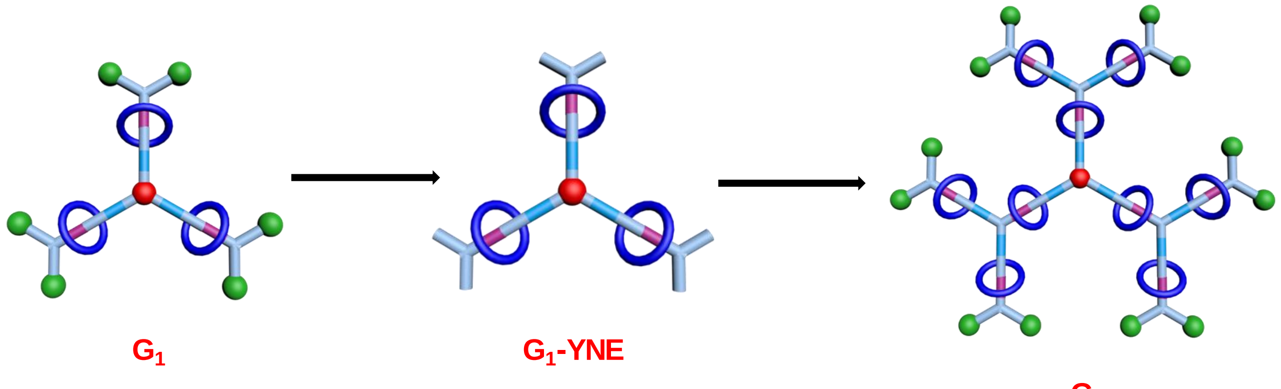

Synthesis of the deprotected first-generation rotaxane-branched dendrimer G_1-YNE: A solution of **G_1** (100 mg, 0.016 mmol) in THF (10.0 mL) and then a solution of Bu_4NF (1.0 M, 0.19 mL) in THF was added dropwise into the reaction flask. The reaction mixture was stirred at room temperature for 4h. The obtained residue was washed by water, then dried with Na_2SO_4 and concentrated. The residue was further purified by column chromatography (SiO_2; DCM) to afford a pale-yellow solid (78 mg, 92%). ^1H NMR (300 MHz, THF-d_8): δ 7.65 (s, 9H), 7.22-7.25 (d, J = 9.0 Hz, 6H), 7.11 (s, 3H), 6.92-6.94 (d, J = 9.0 Hz, 33H), 6.74-6.77 (d, J = 9.0 Hz, 6H), 4.93 (t, 3H), 3.73-4.06 (m, 96H), 3.56 (s, 6H), 2.92-2.93 (m, 6H), 2.58 (m, 6H), 2.22-2.26 (m, 36H), 1.23-1.50 (m, 144H), 0.68-0.80 (m, 18H), 0.11 (m, 6H), -0.09 (m, 6H), -0.84 (m, 6H), -1.35 (m, 6H). ^{31}P NMR (122 MHz, THF-d_8): δ 12.34. ^{13}C NMR (101 MHz, THF-d_8): δ 157.98, 154.97, 150.45, 150.38, 142.18, 132.07, 128.89, 128.51, 123.75, 121.94, 114.69, 114.32, 83.44, 78.63, 69.13, 64.05, 40.26, 32.64, 31.53, 31.44, 31.21, 30.88, 30.48, 30.40, 30.07, 29.76, 29.67, 29.36, 28.05, 24.10, 23.33, 17.37, 17.20, 17.02, 15.63, 15.47, 14.23, 8.58. HRMS (ESI-MS): Calculated for [**G_1-YNE** + 3H]$^{3+}$: m/z = 1812.1895; Found: m/z = 1812.3011. [**G_1-YNE** + 4H]$^{4+}$: m/z = 1359.3922; Found: m/z = 1359.3638.

Synthesis of the second-generation rotaxane-branched dendrimer G_2: A mixture of **G_1-YNE** (41 mg, 0.008 mmol) and [2]rotaxane **2** (110 mg, 0.05 mmol) were added in a Schlenk flask, the Schlenk flask was then evacuated and back-filled with N_2 three times. Next, degassed diethylamine (8.0 mL) and a catalytic amount of CuI were added under an inert atmosphere. The reaction was stirred overnight at room temperature. The solvent was evaporated and the residue was purified by column chromatography (SiO_2; DCM/EA) and gel permeation chromatography (GPC) to yield a pale-yellow solid **G_2** (83 mg, 62%). ^1H NMR (300 MHz, THF-d_8): δ 7.64 (s), 7.53 (s), 7.18 (m), 7.11 (s, 9H), 6.93-6.94 (m), 6.73-6.76 (d, J = 9.0 Hz), 4.72 (t), 3.73-4.07 (m), 3.01 (m), 2.43 (s), 2.23-2.25 (m), 1.23-1.53 (m), 1.16 (s) , 0.58-0.80 (m), 0.18 (m), 0.07 (m), -0.67 (m), -1.13 (m). ^{31}P NMR (122 MHz,

THF-d_8): δ 12.53. ^{13}C NMR (101 MHz, THF-d_8): δ 157.93, 154.90, 150.44, 150.35, 142.08, 132.11, 128.86, 128.84, 128.21, 124.41, 121.96, 114.62, 114.28, 107.61, 90.38, 69.08, 64.00, 40.31, 31.46, 31.29, 30.75, 29.83, 29.67, 29.44, 27.94, 24.21, 18.89, 17.34, 17.17, 17.00, 15.75, 15.69, 15.65, 12.05, 8.61. MALDI-TOF-MS: Calculated for M_r = 17876.5 Da; Found: m/z = 17944.7.

Supplementary Figure 27: Synthesis of the third-generation rotaxane-branched dendrimer **G3**.

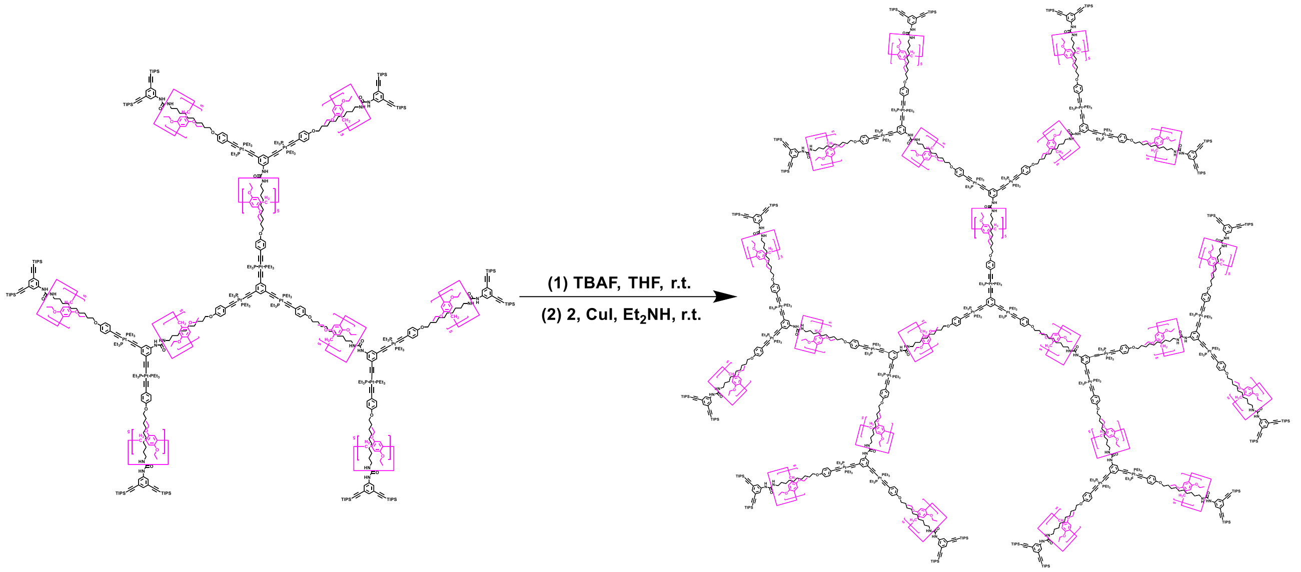

G2 **G3**

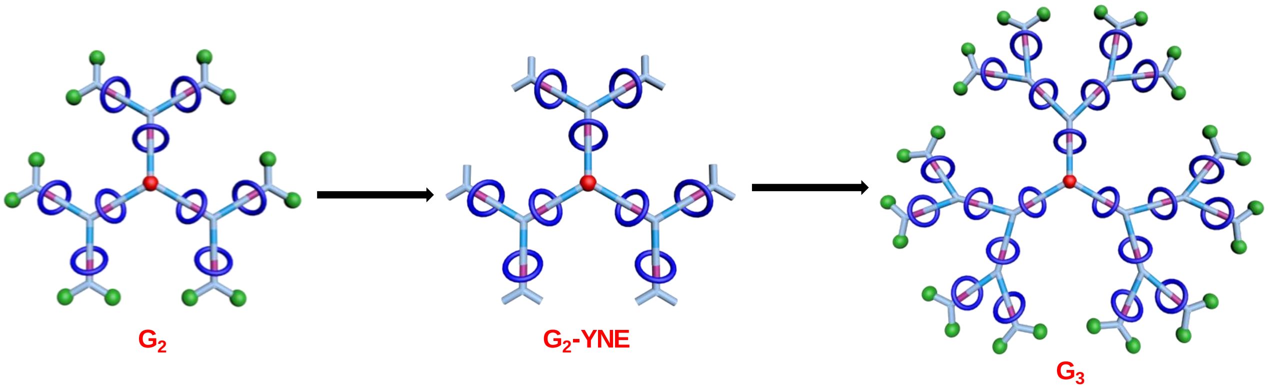

G2 **G2-YNE** **G3**

Synthesis of the deprotected second-generation rotaxane dendrimer G2-YNE: A solution of **G2** (83 mg, 0.0046 mmol) in THF (10.0 mL) and then a solution of Bu_4NF (1.0 M, 0.13 mL) in THF was added dropwise into the reaction flask. The reaction mixture was stirred at room temperature for 4h. The obtained residue was washed by water, then dried with Na_2SO_4 and concentrated. The residue was further purified by column chromatography (SiO_2; DCM/EA) to afford a pale-yellow solid (44 mg, 60%). ^{1}H NMR (300 MHz, THF-d_8): δ 7.65 (s), 7.58 (s), 7.18-7.21 (m), 7.11(t), 6.92-6.94 (m), 6.72-6.75 (d, J = 9.0 Hz), 4.89 (t), 3.83-4.06 (m), 3.73 (s), 3.56 (s), 2.94 (m), 2.21-2.26 (m), 1.23-1.53 (m), 0.89 (m), 0.79 (m), 0.68 (m), -0.08 (m), -0.85 (m), -1.36 (m). ^{31}P NMR (122 MHz,

THF-d_8): δ 12.49. ^{13}C NMR (126 MHz, THF-d_8): δ 157.97, 154.93, 150.44, 150.36, 142.17, 132.10, 128.85, 128.54, 123.78, 121.91, 114.63, 114.58, 114.27, 83.43, 78.69, 69.12, 64.01, 54.70, 40.27, 32.68, 31.55, 31.50, 30.44, 30.41, 30.17, 30.11, 29.66, 23.37, 17.18, 15.75, 15.65, 14.24, 8.61, 8.58. MALDI-TOF-MS: Calculated for M_r = 16000.4 Da; Found: m/z = 16042.6.

Synthesis of the third-generation rotaxane-branched dendrimer G$_3$: A mixture of **G$_2$-YNE** (23 mg, 0.0015 mmol) and [2]rotaxane monomer **2** (42 mg, 0.019 mmol) were added in a Schlenk flask, the Schlenk flask was then evacuated and back-filled with N_2 three times. Next, degassed diethylamine (5 mL) and a catalytic amount of CuI were added under an inert atmosphere. The reaction was stirred overnight at room temperature. The solvent was evaporated and the residue was purified by column chromatography (SiO_2; DCM/EA) and gel permeation chromatography (GPC) to yield a pale-yellow solid **G$_3$** (36 mg, 61%). ^1H NMR (400 MHz, THF-d_8): δ 7.64-7.65 (d), 7.60 (s), 7.18-7.20 (m), 7.10 (s), 6.93 (m), 6.73-6.75 (d), 4.78 (m), 3.84-4.06 (m), 3.73 (s), 3.00-3.02 (m), 2.46 (s), 2.22-2.24 (m), 1.25-1.52 (m), 1.16 (s), 0.79 (m), 0.60 (m), -0.69 (m), -1.14 (m). ^{31}P NMR (122 MHz, THF-d_8): δ 12.52. ^{13}C NMR (101 MHz, THF-d_8): δ 154.90, 150.46, 150.37, 142.09, 132.12, 128.90, 128.86, 124.42, 121.97, 114.66, 114.32, 107.63, 90.39, 64.07, 64.02, 32.67, 30.43, 30.10, 29.82, 29.67, 23.36, 18.89, 17.38, 17.21, 17.03, 15.75, 15.68, 15.65, 14.23, 12.06, 8.61, 1.17.

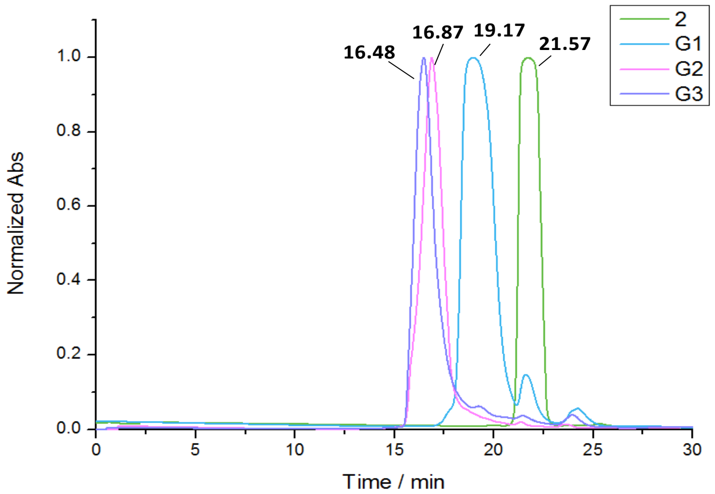

Supplementary Figure 28: Retention time of the [2]rotaxane **2** and rotaxane-branched dendrimers **G$_1$-G$_3$**. (All products displayed a single dominant peak. In addition, compared with the key [2]rotaxane building block **2**, the dendrimers **G$_1$–G$_3$** displayed a decrease of retention time from 21.57 min (**2**) to 19.17 min (**G$_1$**), 16.87 min (**G$_2$**), and 16.48 min (**G$_3$**), thus revealing the size increase of the obtained rotaxane-branched dendrimers with the growth of dendrimer generation.

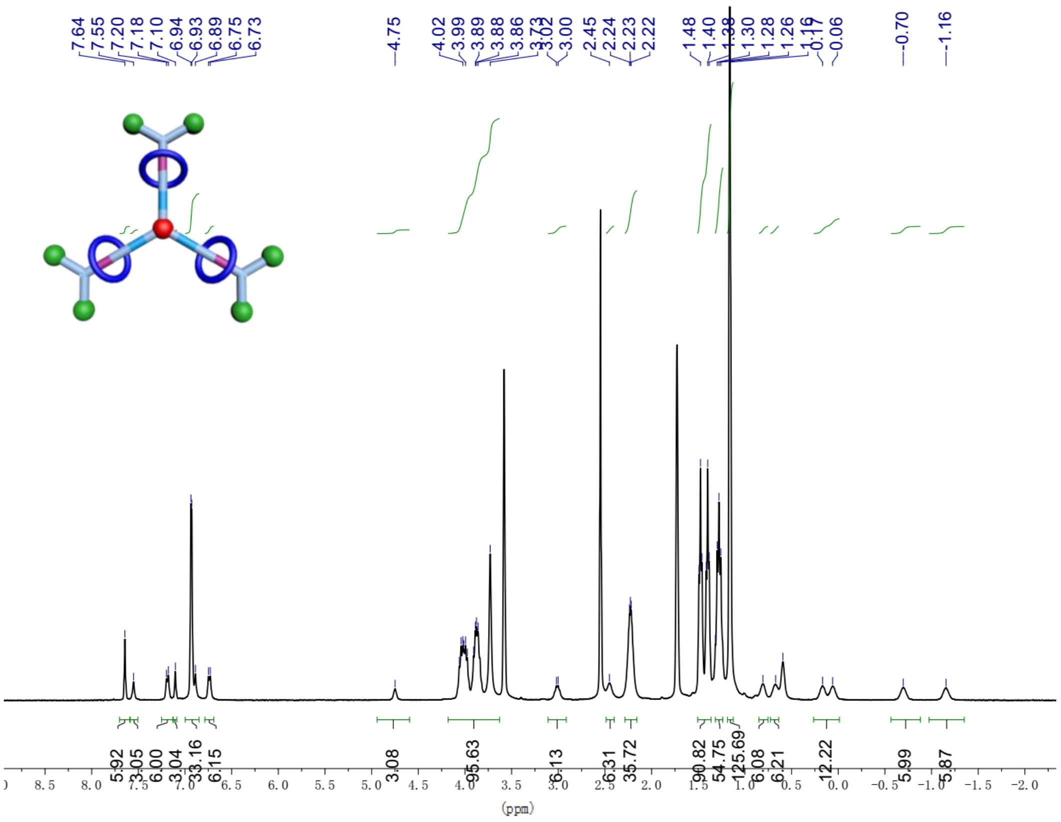

Supplementary Figure 29: ^{1}H NMR spectrum (THF-d_8, 298 K, 400 MHz) of rotaxane-branched dendrimer **G$_1$**.

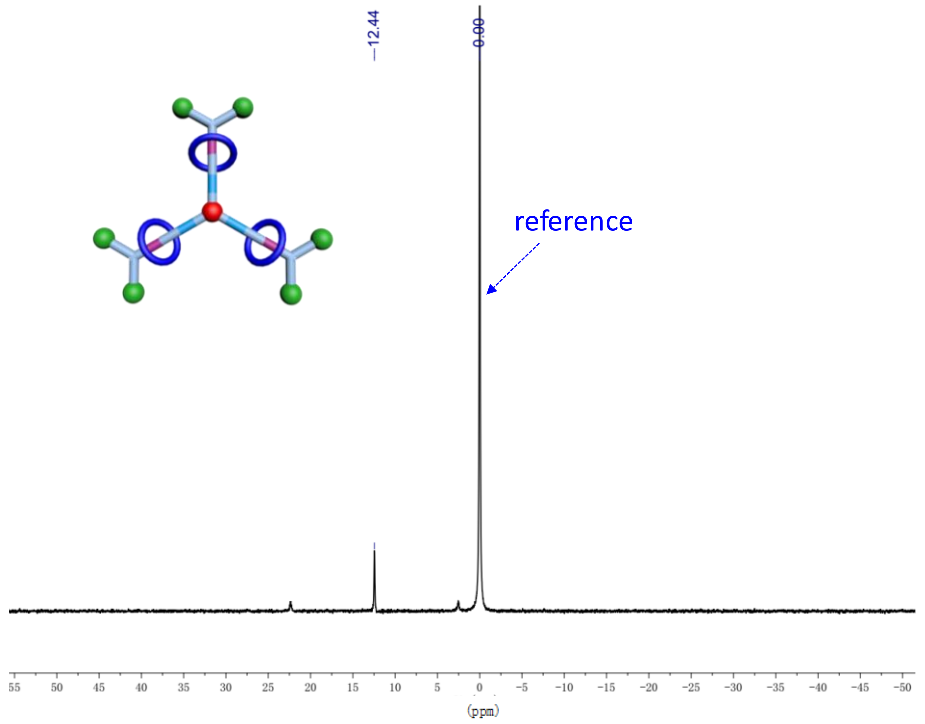

Supplementary Figure 30: ^{31}P NMR spectrum (THF-d_8, 298 K, 122 MHz) of rotaxane-branched dendrimer **G$_1$**.

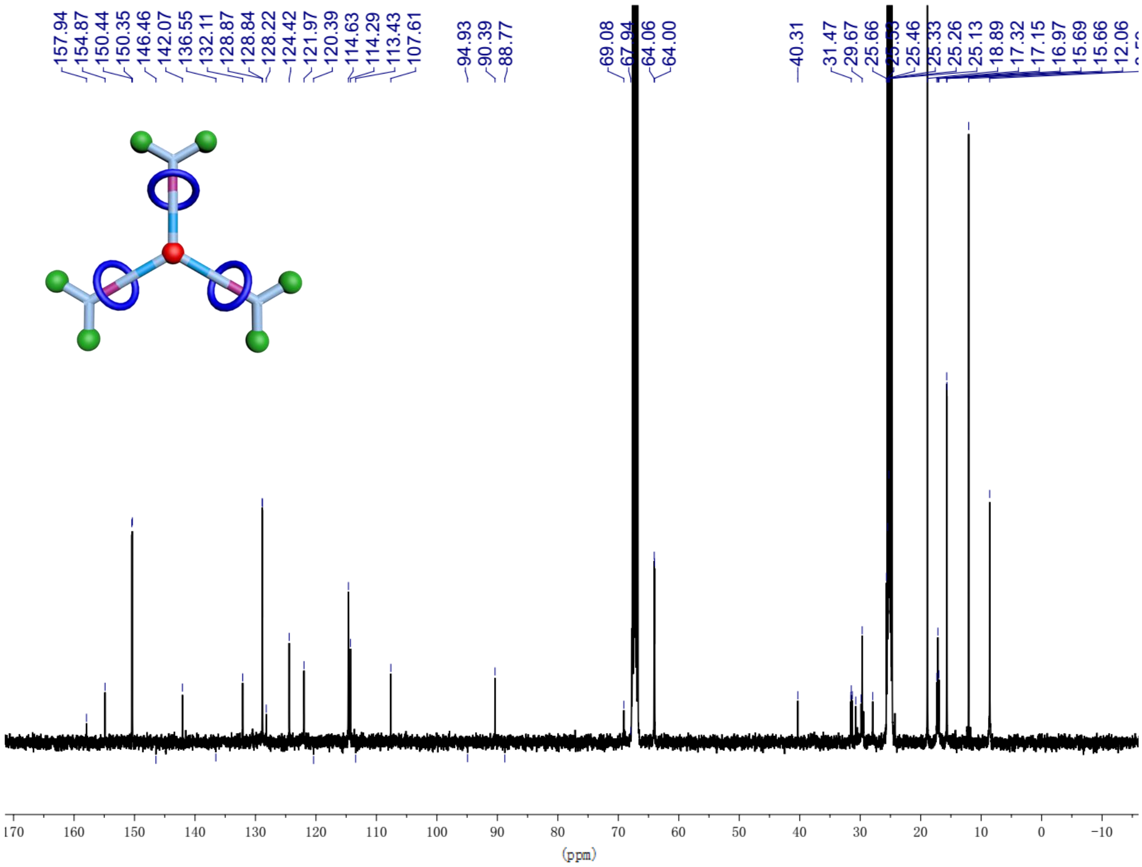

Supplementary Figure 31: ^{13}C NMR spectrum (THF-d_8, 298 K, 101 MHz) of rotaxane-branched dendrimer $\mathbf{G_1}$.

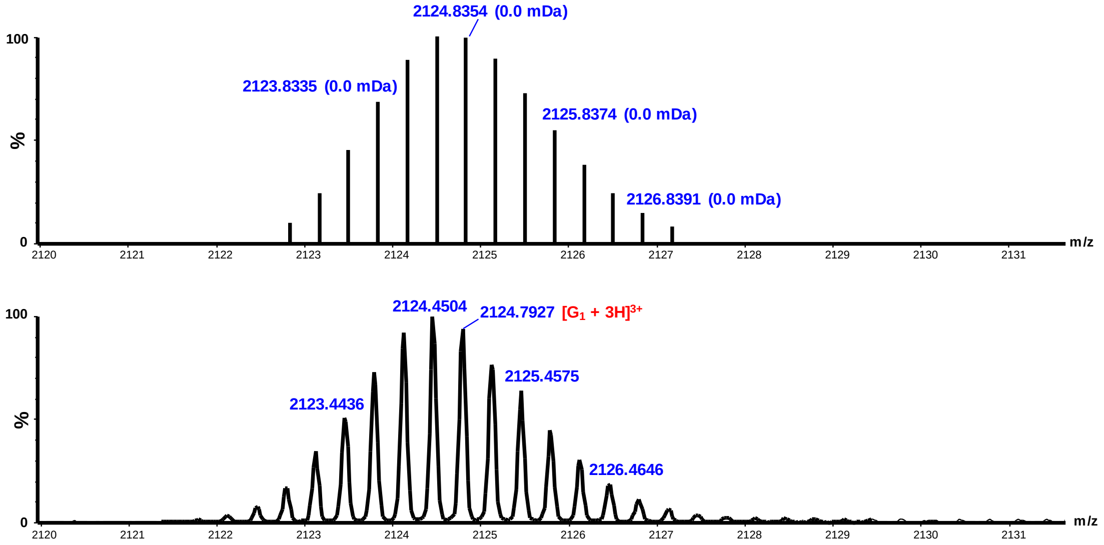

Supplementary Figure 32: Theoretical (*top*) and experimental (*bottom*) ESI-TOF-MS spectrum of rotaxane-branched dendrimer $\mathbf{G_1}$.

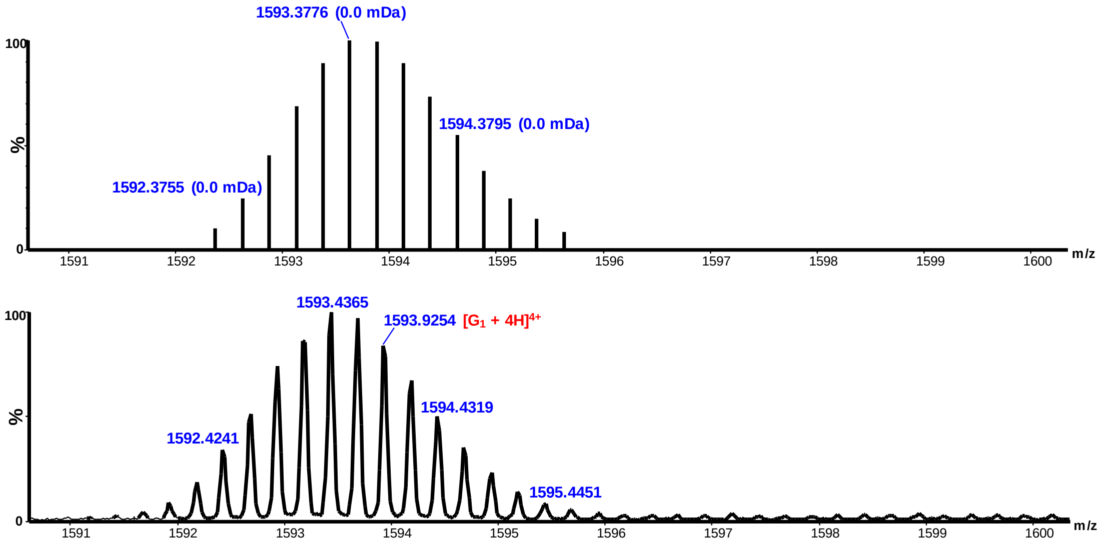

Supplementary Figure 33: Theoretical (*top*) and experimental (*bottom*) ESI-TOF-MS spectrum of rotaxane-branched dendrimer $\mathbf{G_1}$.

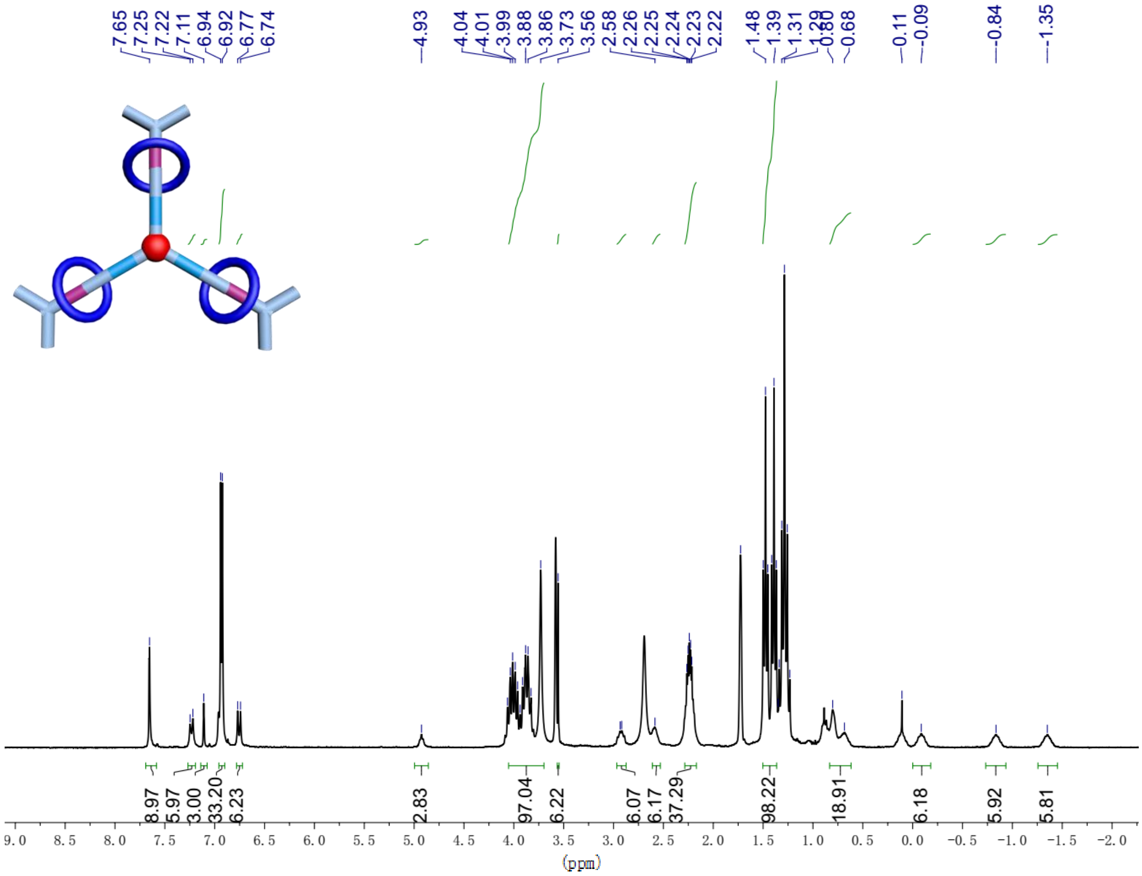

Supplementary Figure 34: ^{1}H NMR spectrum (THF-d_8, 298 K, 300 MHz) of rotaxane-branched dendrimer $\mathbf{G_1}$**-YNE**.

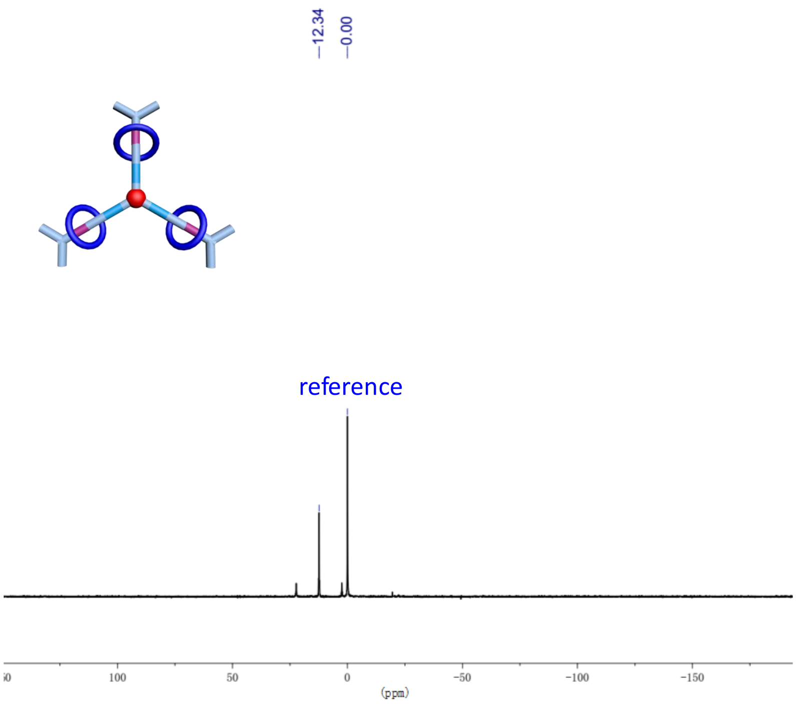

Supplementary Figure 35: ^{31}P NMR spectrum (THF-d_8, 298 K, 122 MHz) of rotaxane-branched dendrimer **G_1-YNE**.

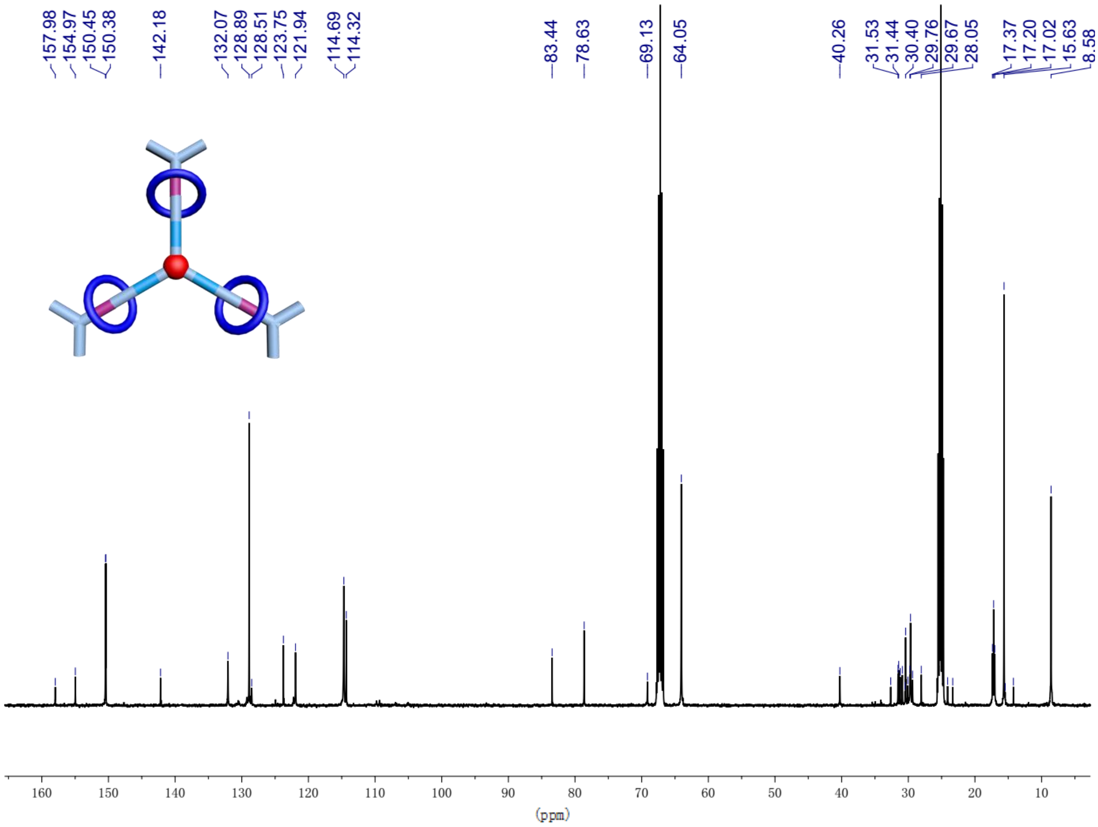

Supplementary Figure 36: ^{13}C NMR spectrum (THF-d_8, 298 K, 101 MHz) of rotaxane-branched dendrimer **G_1-YNE**.

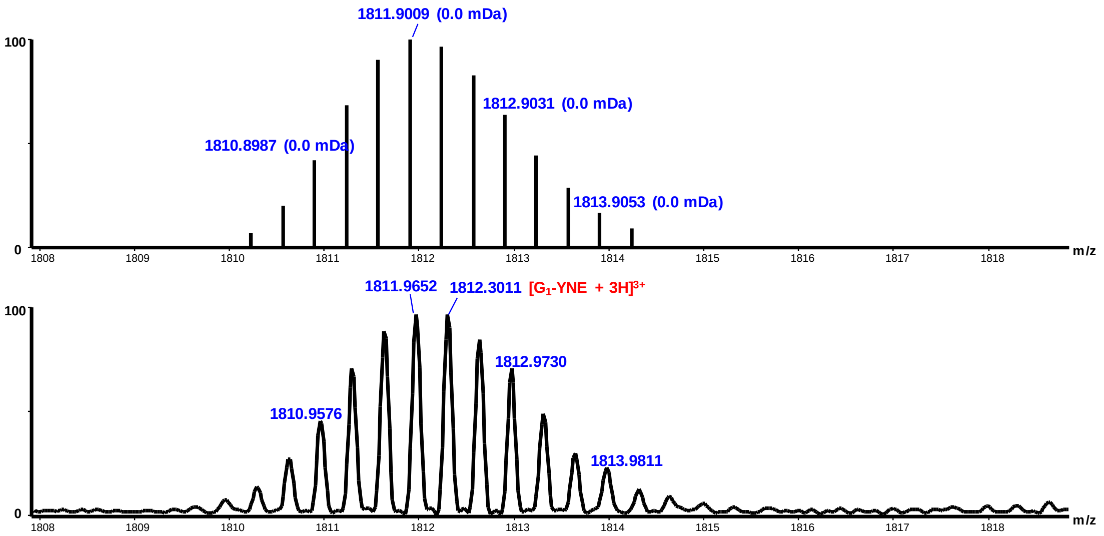

Supplementary Figure 37: Theoretical (*top*) and experimental (*bottom*) ESI-TOF-MS spectrum of rotaxane-branched dendrimer **G$_1$-YNE**.

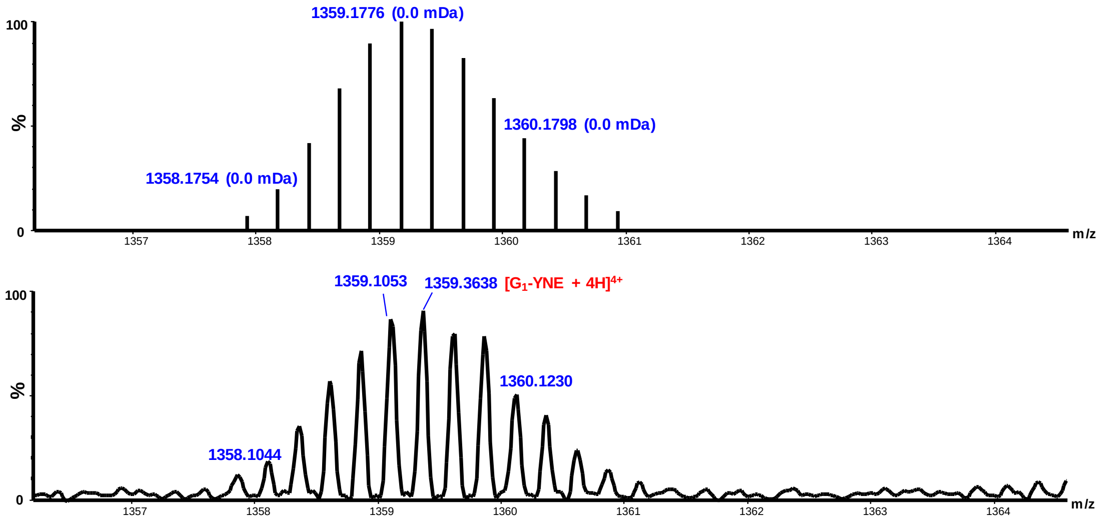

Supplementary Figure 38: Theoretical (*top*) and experimental (*bottom*) ESI-TOF-MS spectrum of rotaxane-branched dendrimer **G$_1$-YNE**.

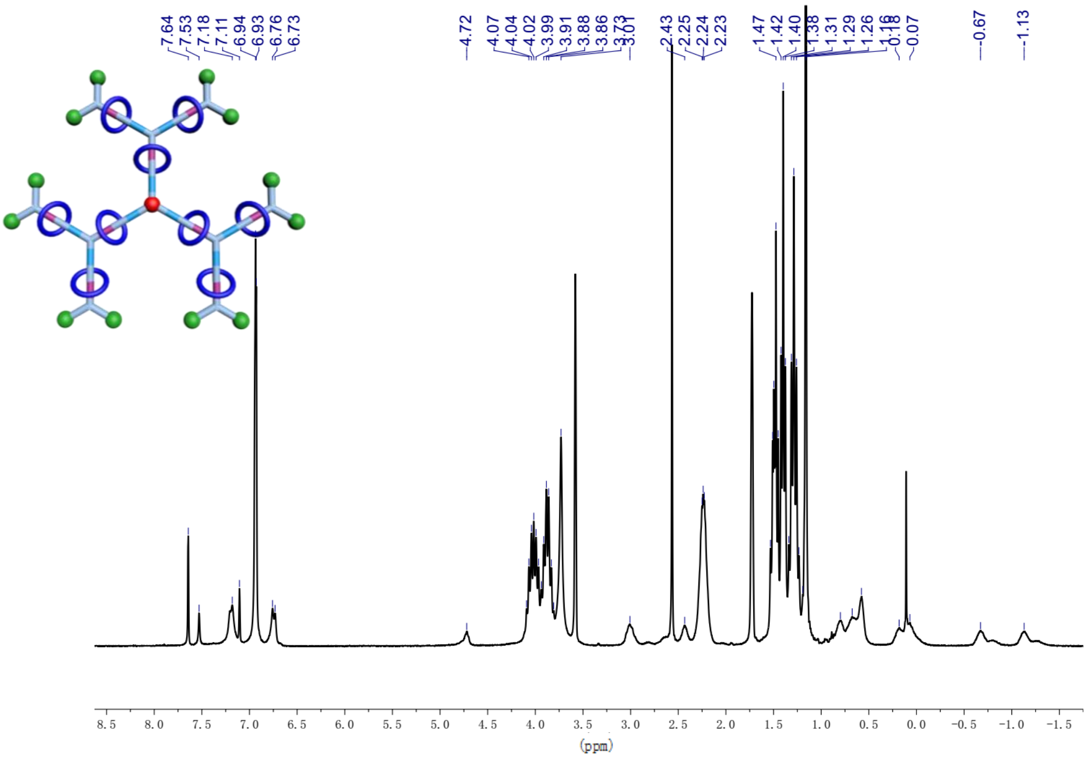

Supplementary Figure 39: ^{1}H NMR spectrum (THF-d_8, 298 K, 300 MHz) of rotaxane-branched dendrimer **G$_2$**.

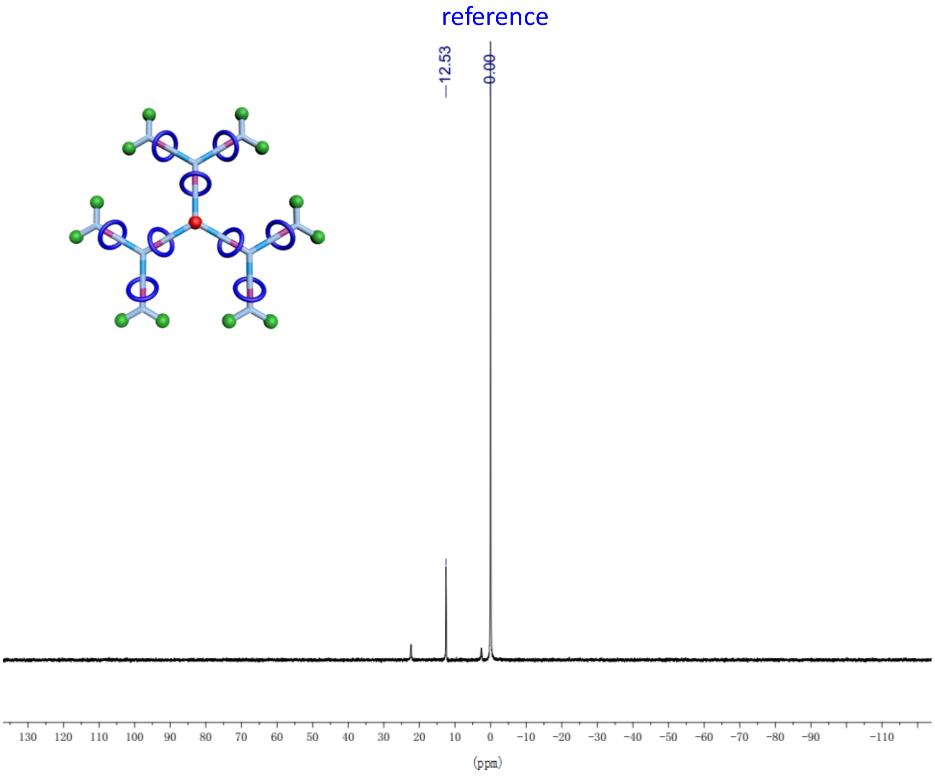

Supplementary Figure 40: ^{31}P NMR spectrum (THF-d_8, 298 K, 122 MHz) of rotaxane-branched dendrimer **G$_2$**.

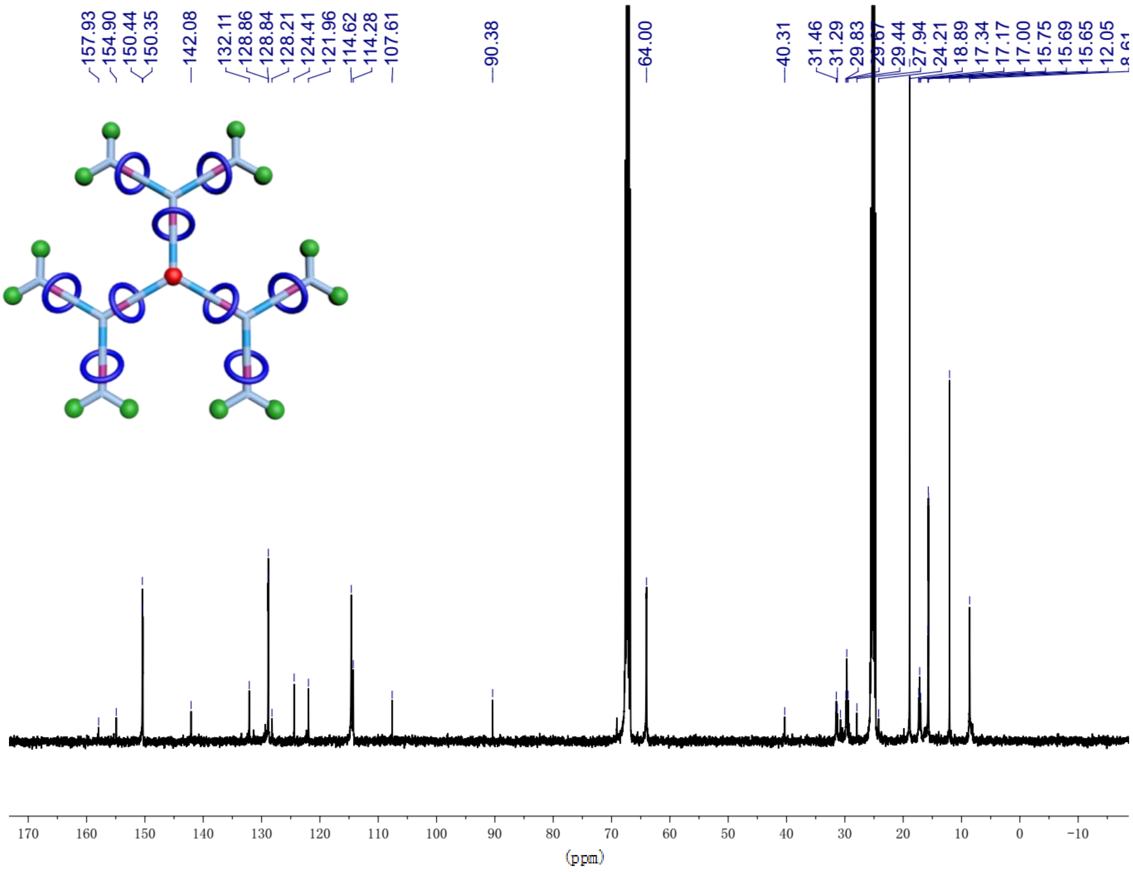

Supplementary Figure 41: ^{13}C NMR spectrum (THF-d_8, 298 K, 101 MHz) of rotaxane-branched dendrimer **G$_2$**.

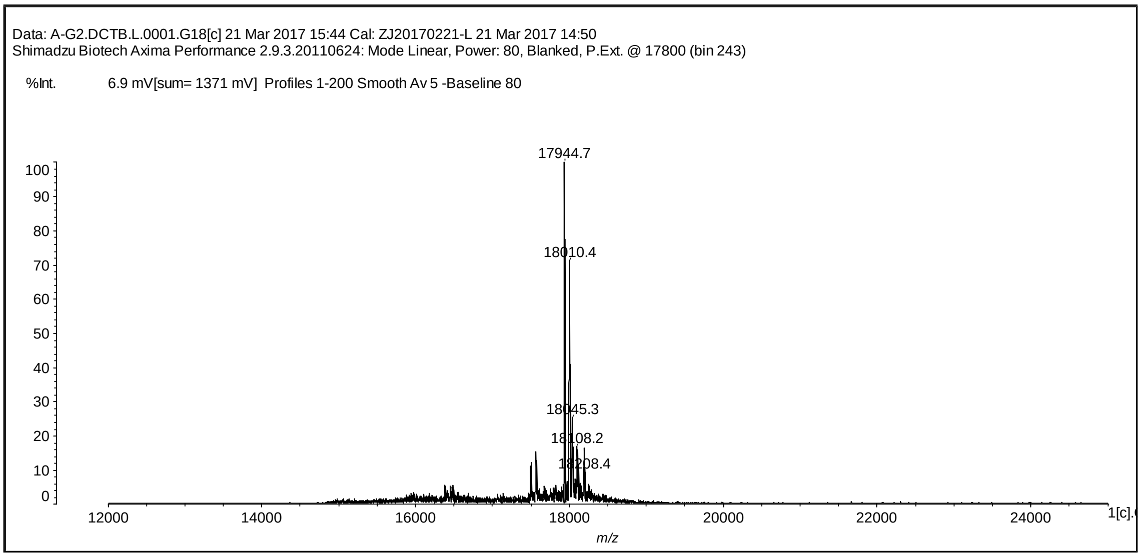

Supplementary Figure 42: MALDI-TOF-MS spectrum of rotaxane-branched dendrimer **G$_2$**.

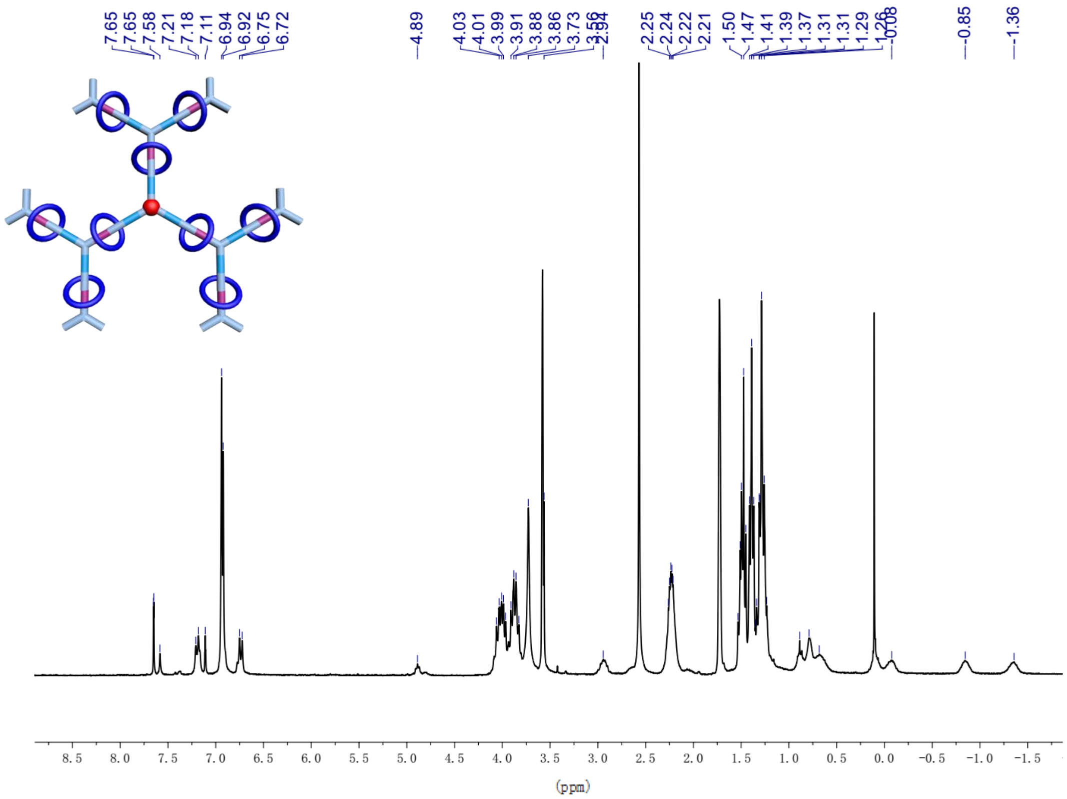

Supplementary Figure 43: ^{1}H NMR spectrum (THF-d_8, 298 K, 300 MHz) of rotaxane-branched dendrimer **G2-YNE**.

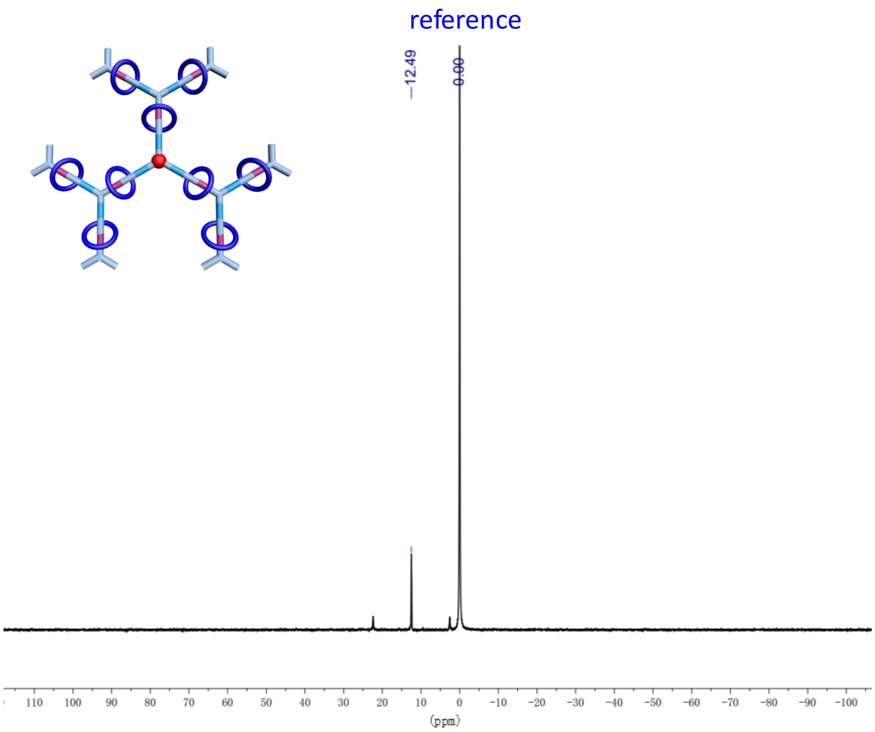

Supplementary Figure 44: ^{31}P NMR spectrum (THF-d_8, 298 K, 122 MHz) of rotaxane-branched dendrimer **G2-YNE**.

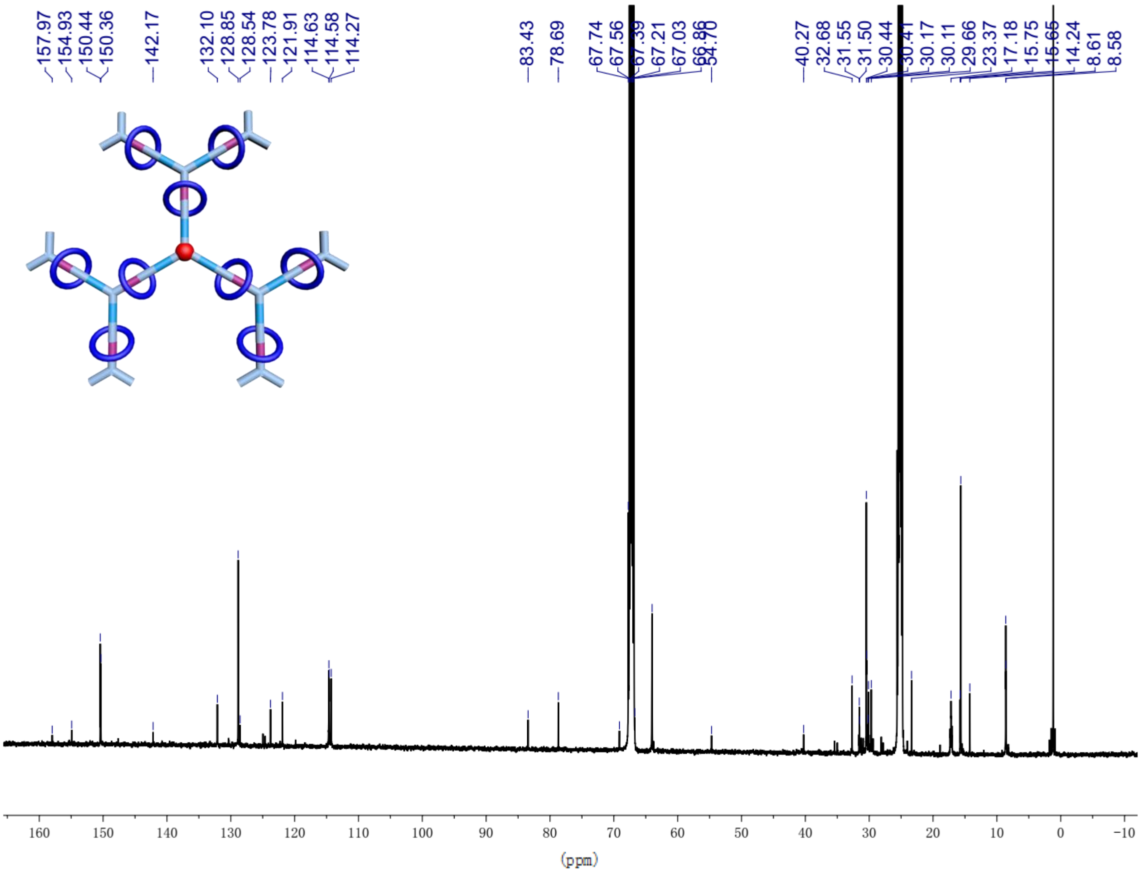

Supplementary Figure 45: ^{13}C NMR spectrum (THF-d_8, 298 K, 101 MHz) of rotaxane-branched dendrimer **G$_2$-YNE**.

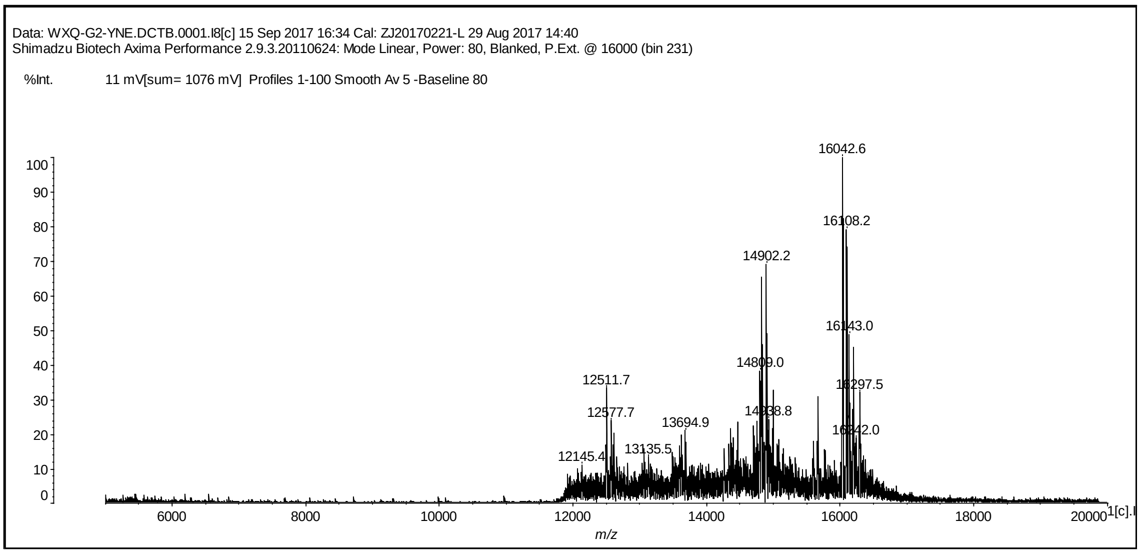

Supplementary Figure 46: MALDI-TOF-MS spectrum of rotaxane-branched dendrimer **G$_2$-YNE**.

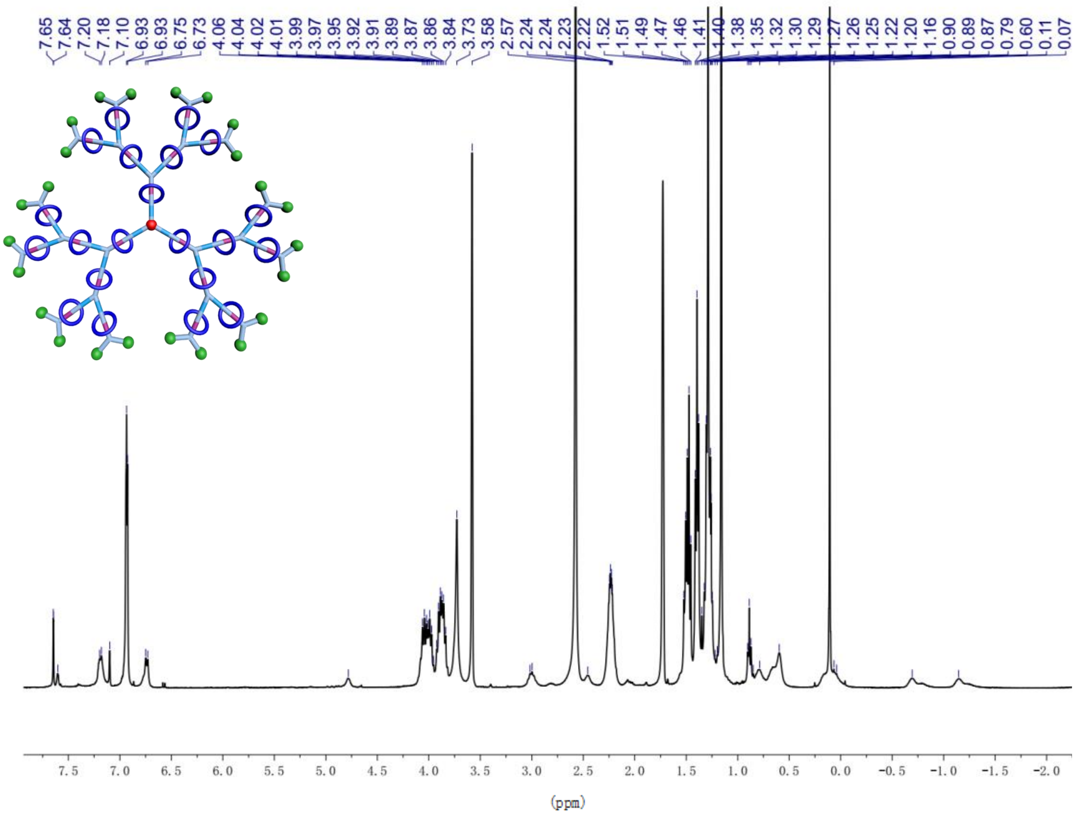

Supplementary Figure 47: ^{1}H NMR spectrum (THF-d_8, 298 K, 300 MHz) of rotaxane-branched dendrimer **G3**.

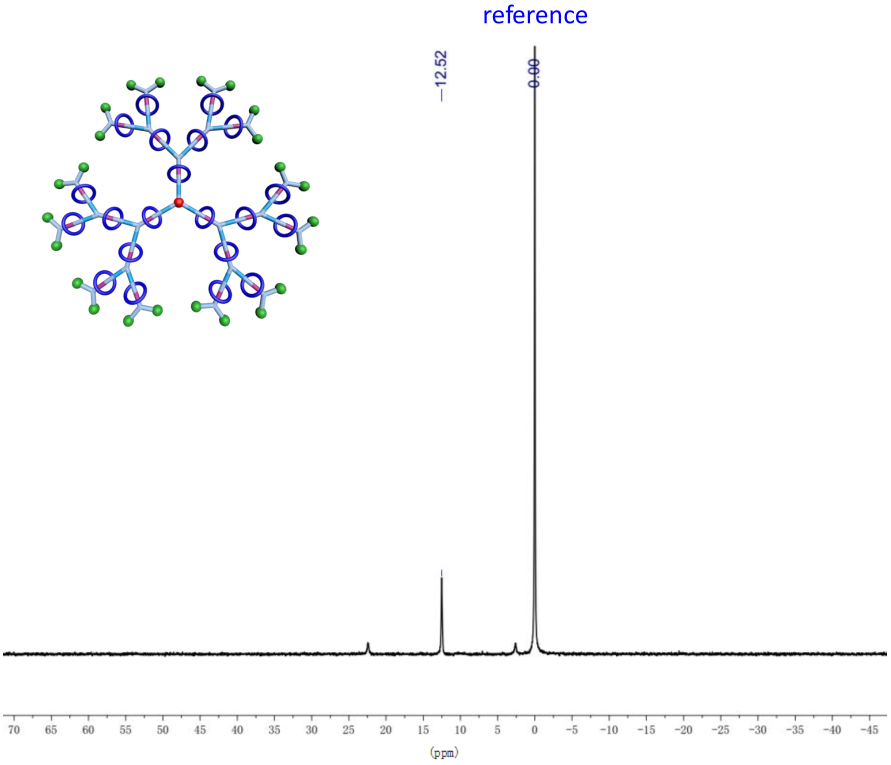

Supplementary Figure 48: ^{31}P NMR spectrum (THF-d_8, 298 K, 122 MHz) of rotaxane-branched dendrimer **G3**.

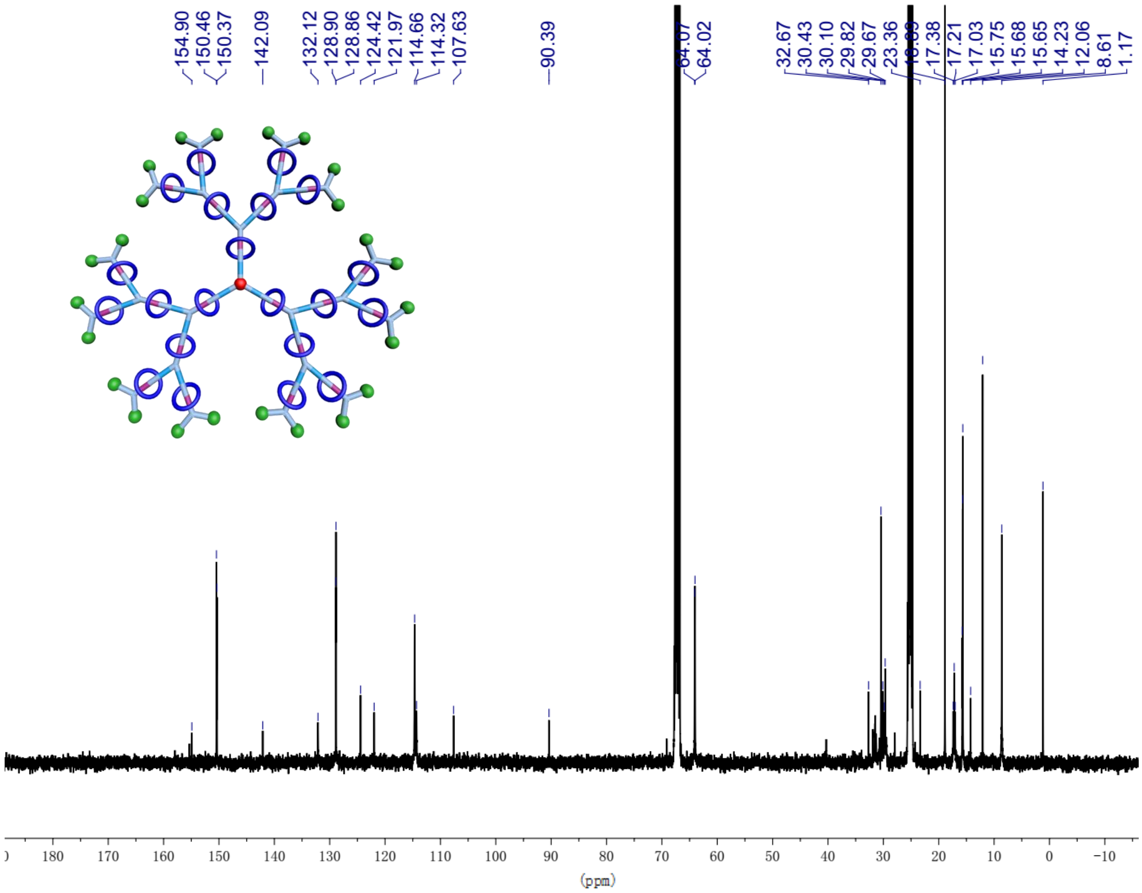

Supplementary Figure 49: ^{13}C NMR spectrum (THF-d_8, 298 K, 101 MHz) of rotaxane-branched dendrimer **G$_3$**.

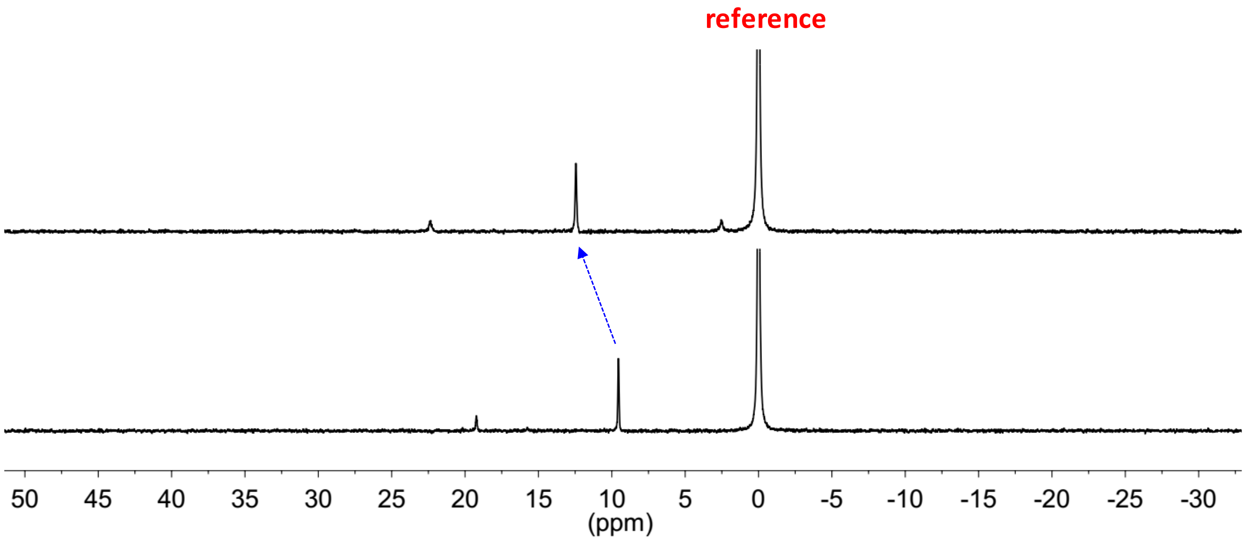

Supplementary Figure 50: ^{31}P NMR spectra (THF-d_8, 298 K, 122 MHz) of rotaxane-branched dendrimer **G$_1$** (*top*) and [2]rotaxane building block **2** (*bottom*).

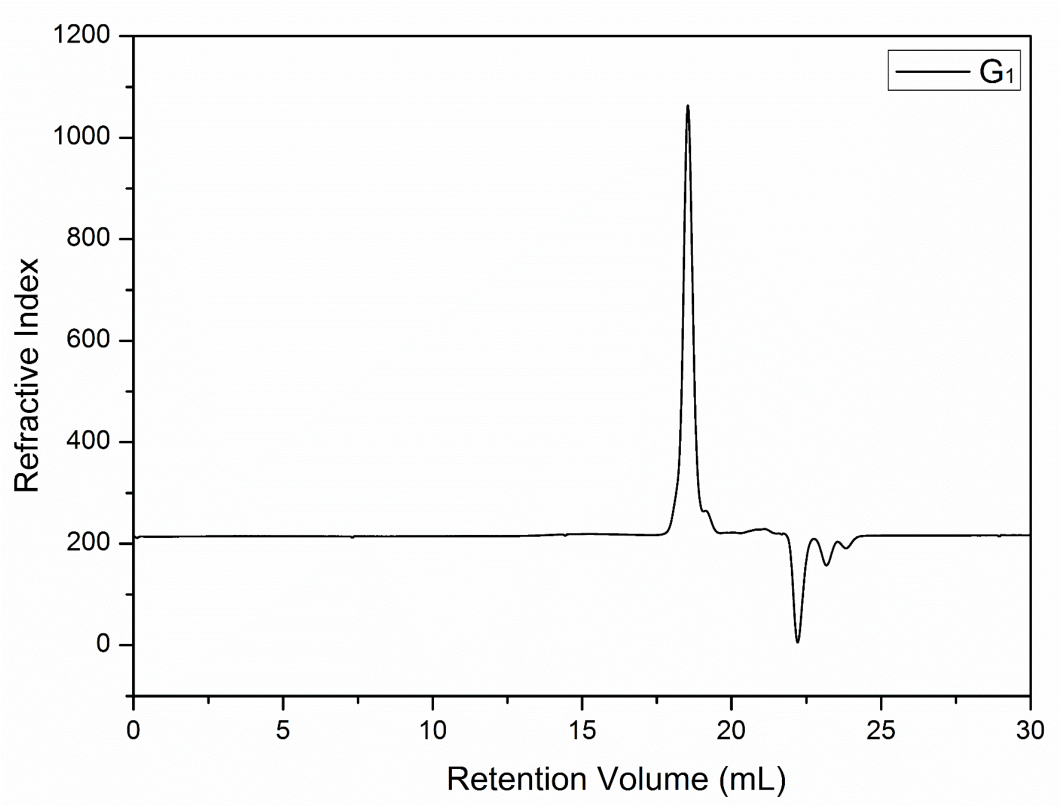

Supplementary Figure 51: GPC spectrum of the rotaxane-branched dendrimer $\mathbf{G_1}$.

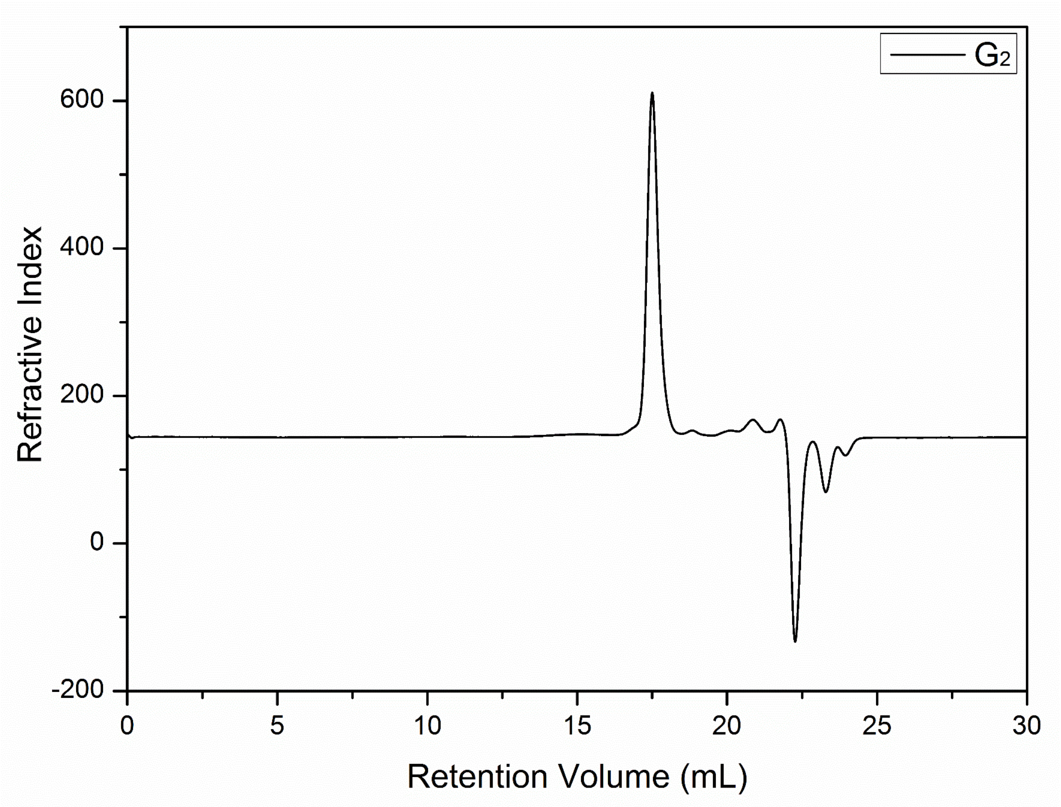

Supplementary Figure 52: GPC spectrum of the rotaxane-branched dendrimer $\mathbf{G_2}$.

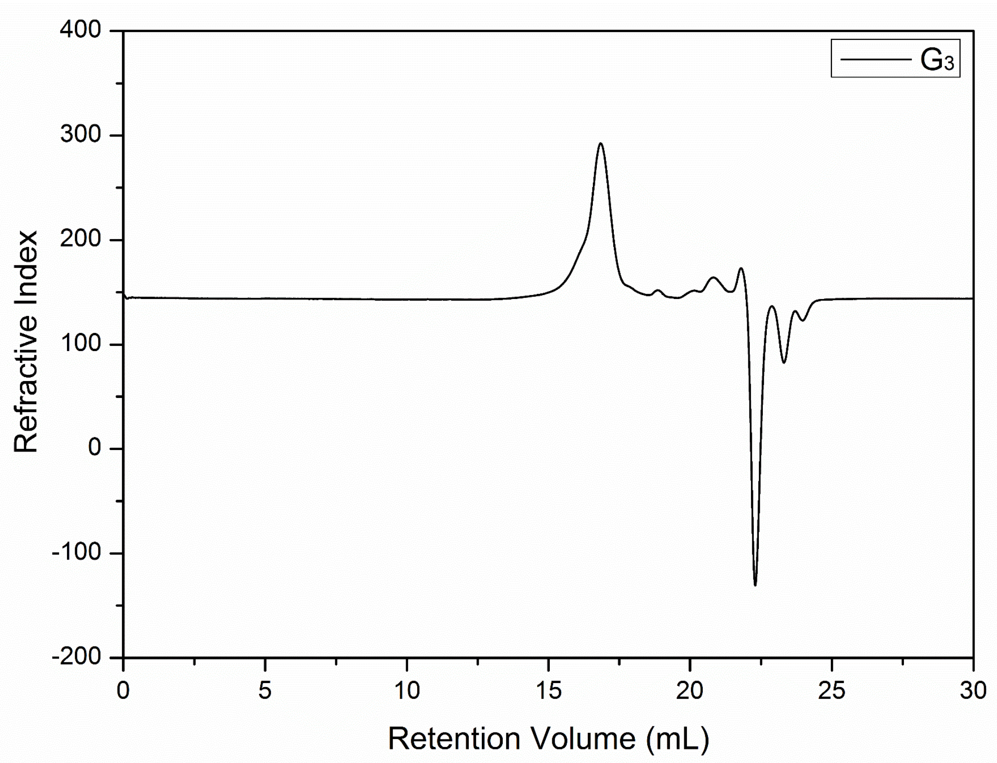

Supplementary Figure 53: GPC spectrum of the rotaxane-branched dendrimer **G**$_3$.

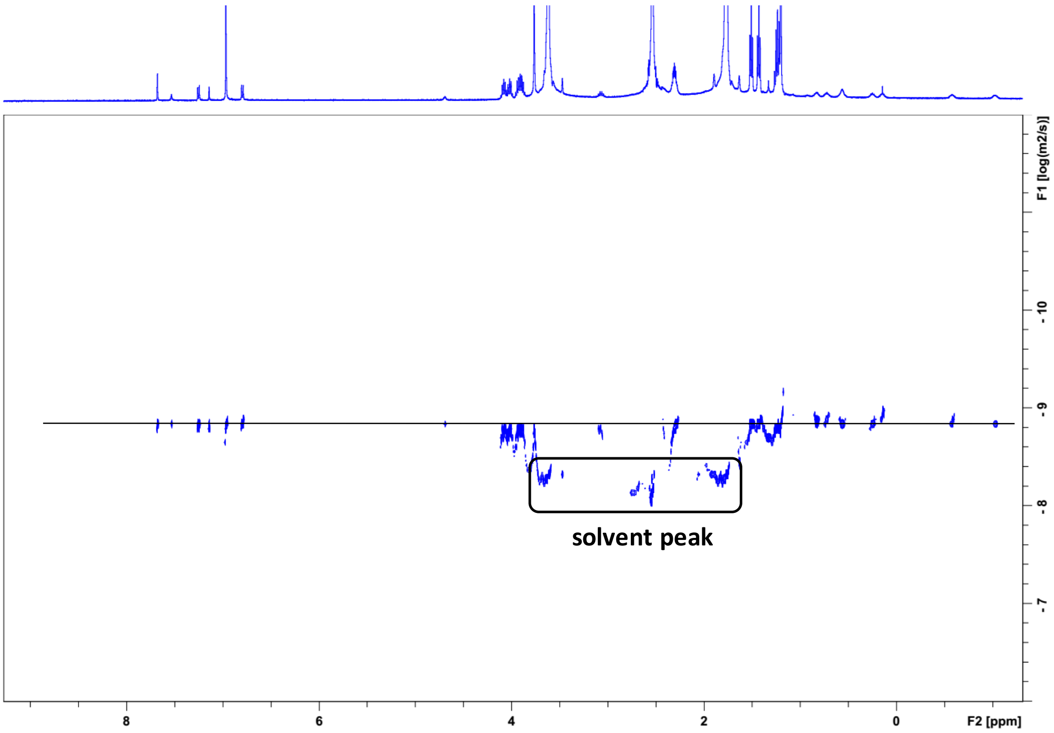

Supplementary Figure 54: 2-D DOSY spectrum (THF-d_8, 298 K, 500 MHz) of [2]rotaxane building block **2**.

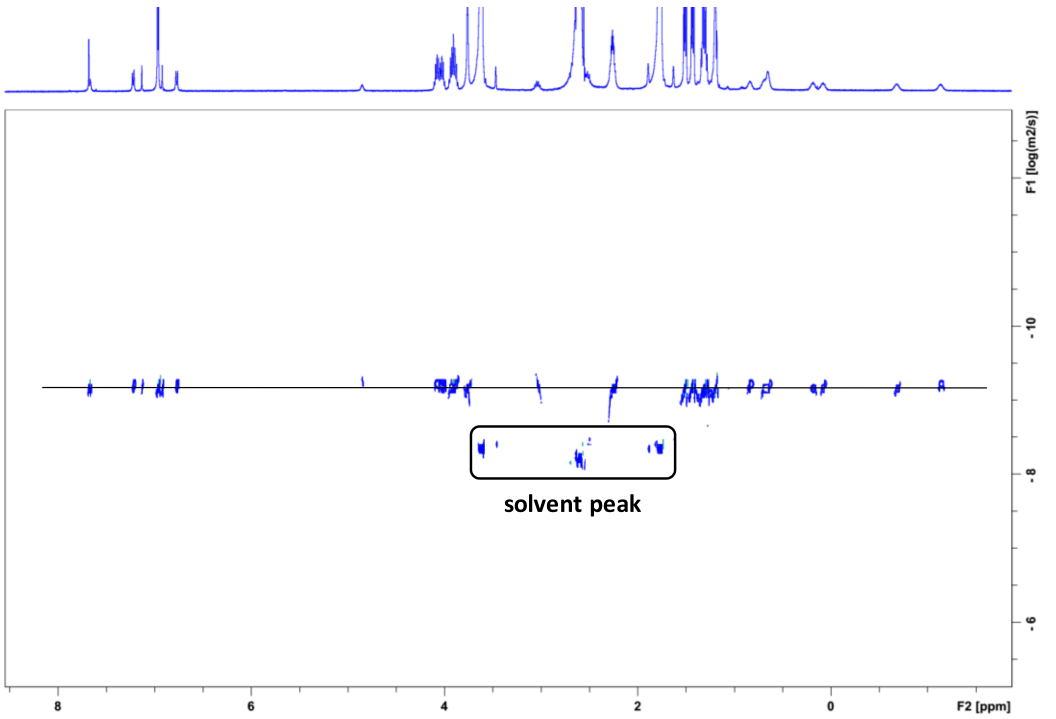

Supplementary Figure 55: 2-D DOSY spectrum (THF-d_8, 298 K, 500 MHz) of rotaxane-branched dendrimer $\mathbf{G_1}$.

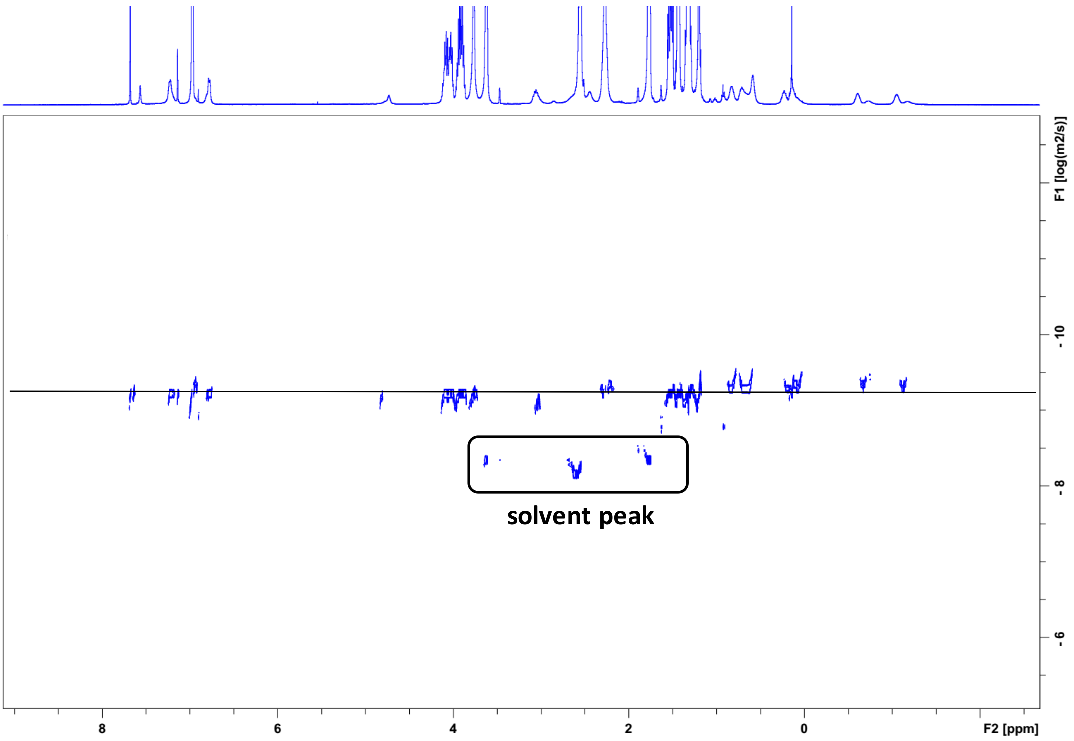

Supplementary Figure 56: 2D-DOSY spectrum (THF-d_8, 298 K, 500 MHz) of rotaxane-branched dendrimer $\mathbf{G_2}$.

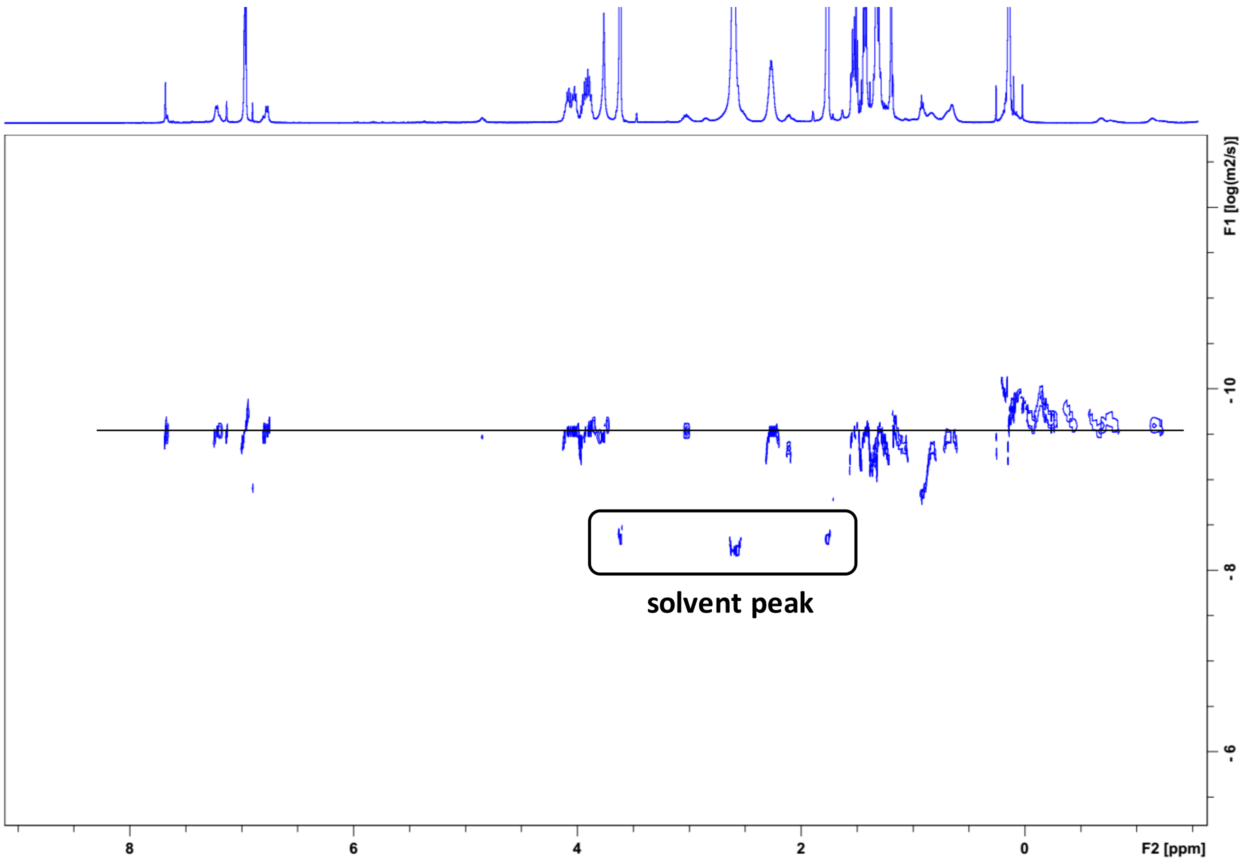

Supplementary Figure 57: 2D-DOSY spectrum (THF-d_8, 298 K, 500 MHz) of rotaxane-branched dendrimer **G3**.

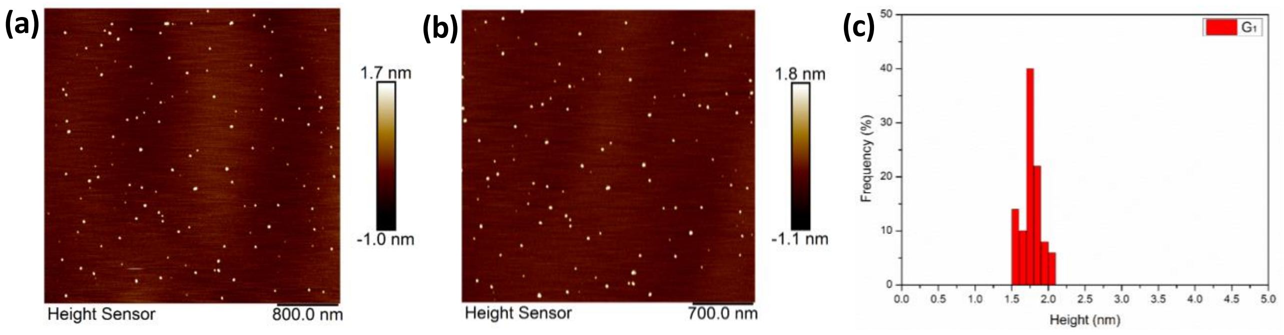

Supplementary Figure 58: (a-b) AFM images and (c) height distributions of the rotaxane-branched dendrimer **G_1**. The height range is 1.76 ±0.26 nm.

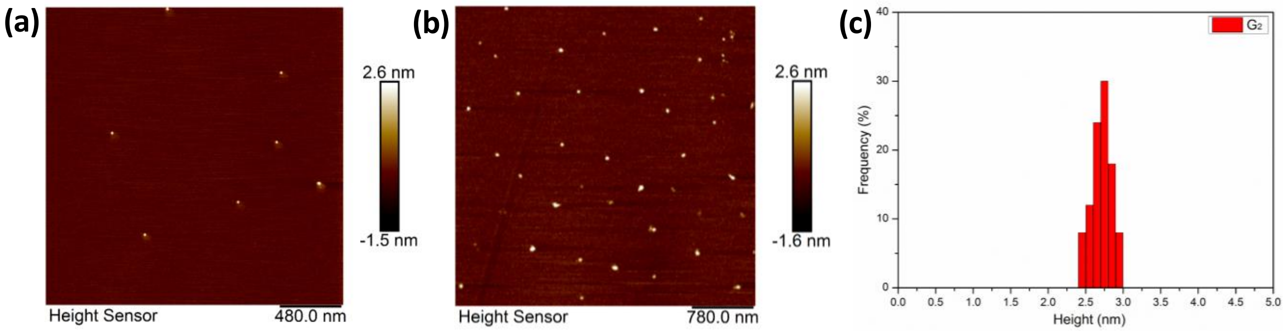

Supplementary Figure 59: (a-b) AFM images and (c) height distributions of the rotaxane-branched dendrimer **G_2**. The height range is 2.70 ±0.29 nm.

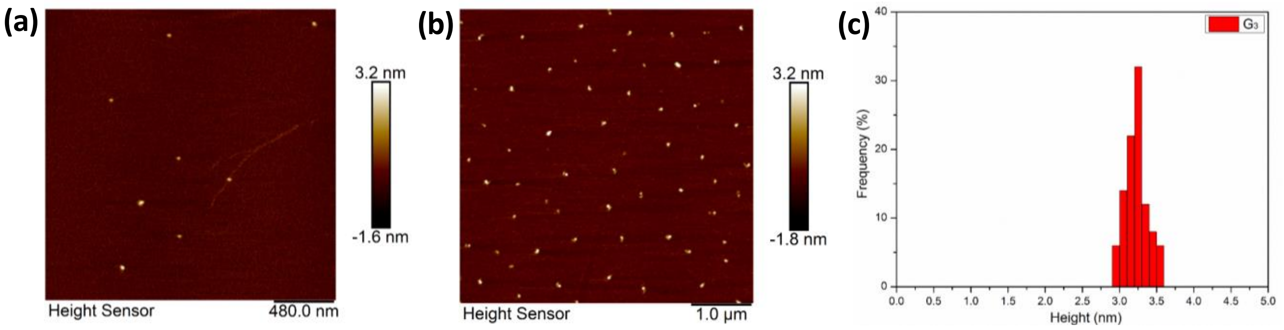

Supplementary Figure 60: (a-b) AFM images and (c) height distributions of the rotaxane-branched dendrimer **G_3**. The height range is 3.21 ±0.34 nm.

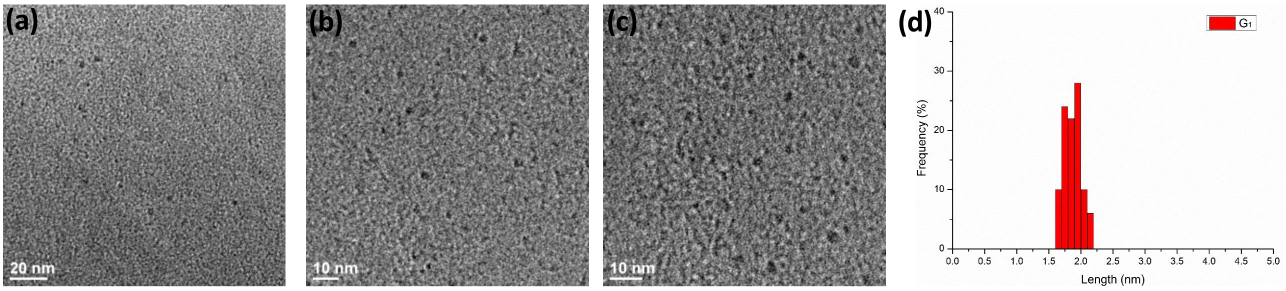

Supplementary Figure 61: TEM images and height distributions of the rotaxane-branched dendrimer **G$_1$**. The height range is 1.86 ±0.24 nm.

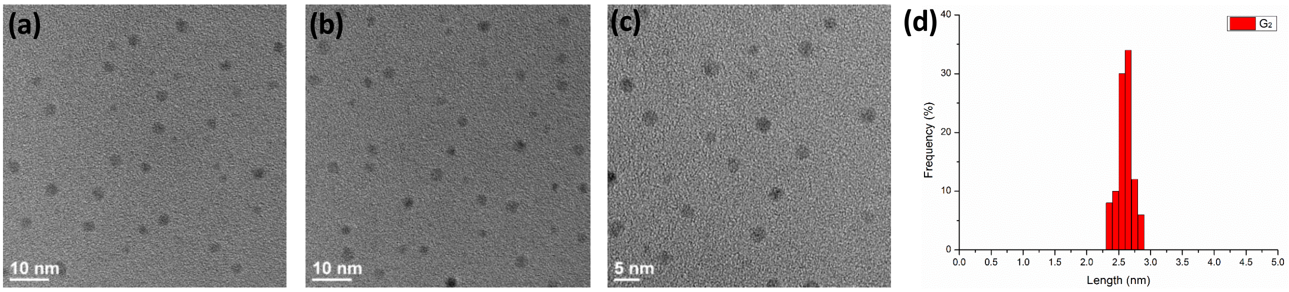

Supplementary Figure 62: TEM images and height distributions of the rotaxane-branched dendrimer **G$_2$**. The height range is 2.60 ±0.27 nm.

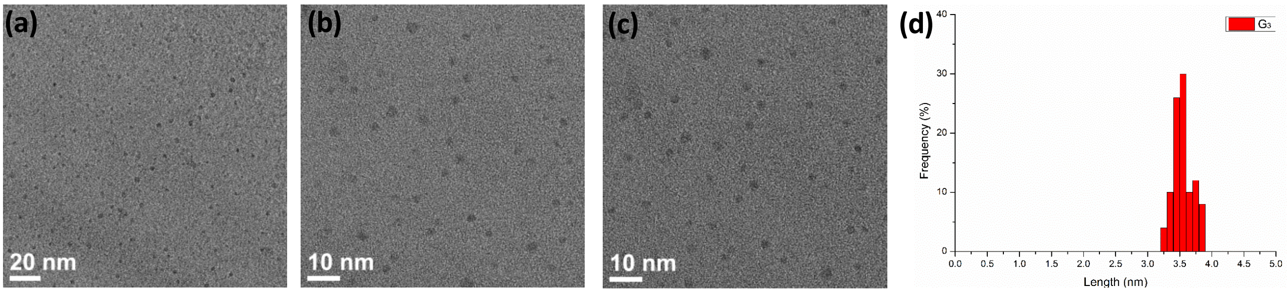

Supplementary Figure 63: TEM images and height distributions of the rotaxane-branched dendrimer **G$_3$**. The height range is 3.55 ±0.32 nm

Supplementary Table 1: Photophysical properties of [2]rotaxane **2** and rotaxane-branched dendrimers **G1-G3**.

It was found that the growth of the dendrimer generation led to the progression of the molar absorptivity (for 343 nm, ε increased from 10000 (**2**) to 715000 (**G3**); for 291nm, ε increased from 52000 (**2**) to 912000 (**G3**)) of these absorptions caused by the number increase of platinum-acetylide bonds in the rotaxane-branched dendrimers.

Compound	Medium	Absorption λ_{abs}/nm (ε/dm^3 mol^{-1} cm^{-1})
**2**	THF	291 (52000), 343 (10000)
**G_1**	THF	291 (276000), 343 (235000)
**G_2**	THF	291 (496000), 343 (400000)
**G_3**	THF	291 (912000), 343 (715000)

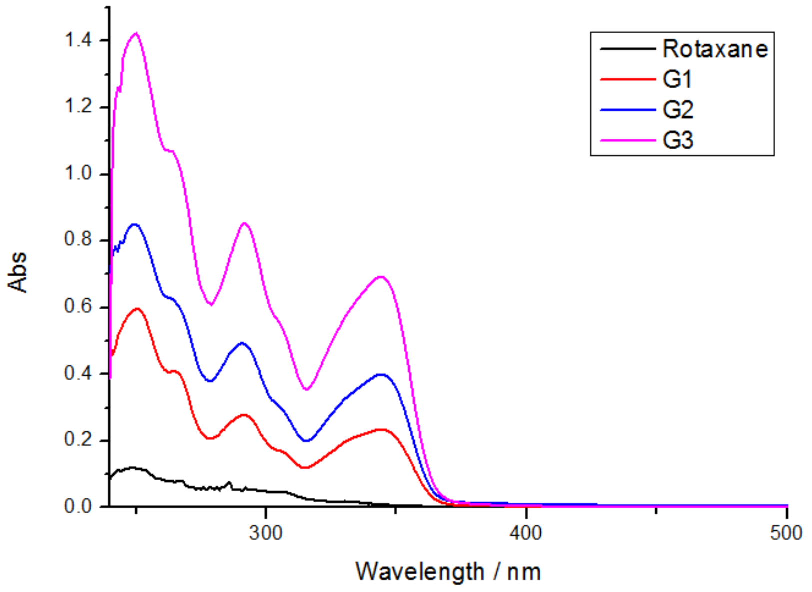

Supplementary Figure 64: UV-Vis spectra of [2]rotaxane **2** and rotaxane-branched dendrimers in THF (10^{-6} M). (Since the platinum-acetylide units in the resultant rotaxane-branched dendrimers were well separated, no significant shifts of the λmax values were observed, which indicated that the interaction among the inserted platinum-acetylide moieties was relatively weak.)

Section D. Synthesis and characterizaiton of the model dendrimers.

Supplementary Figure 65: The synthesis routes of (a) model [2]rotaxane building block **2-a** without urea moiety as the stimuli-responsive site and (b) model first-generation rotaxane dendrimer **G1-a** from corresponding building block **2-a**.

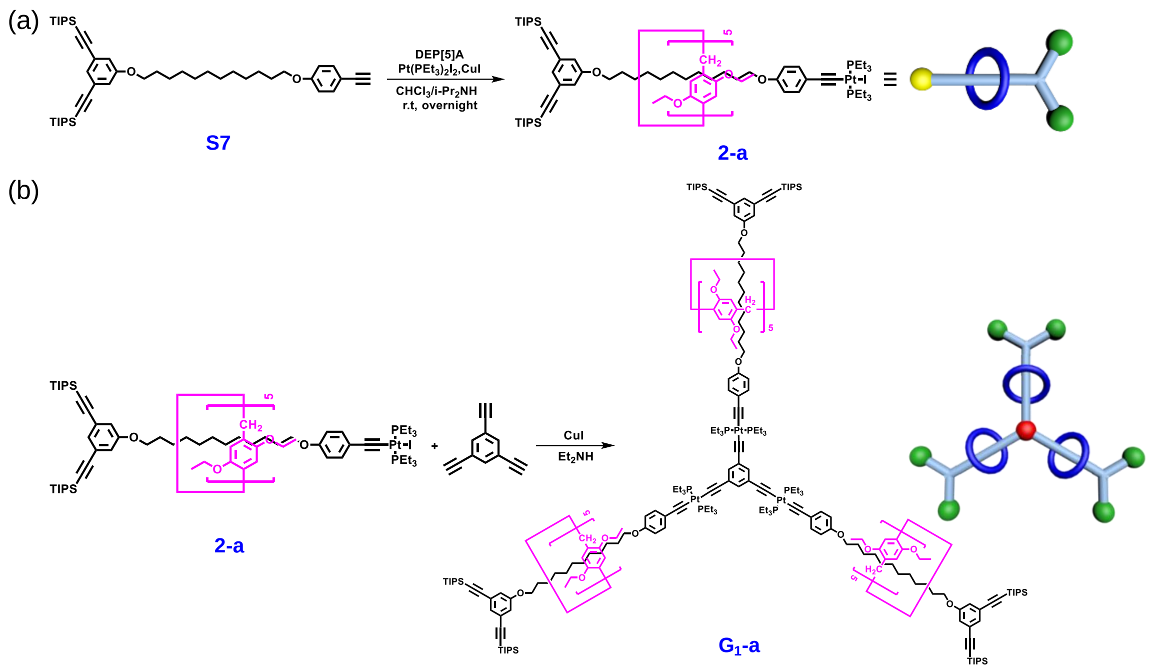

Supplementary Figure 66: The synthesis routes of (a) platinum-acetylide building block **2-b** without pillar[5]arene as the wheel and (b) model first-generation platinum-acetylide dendrimer **G1-b** from corresponding building block **2-b**.

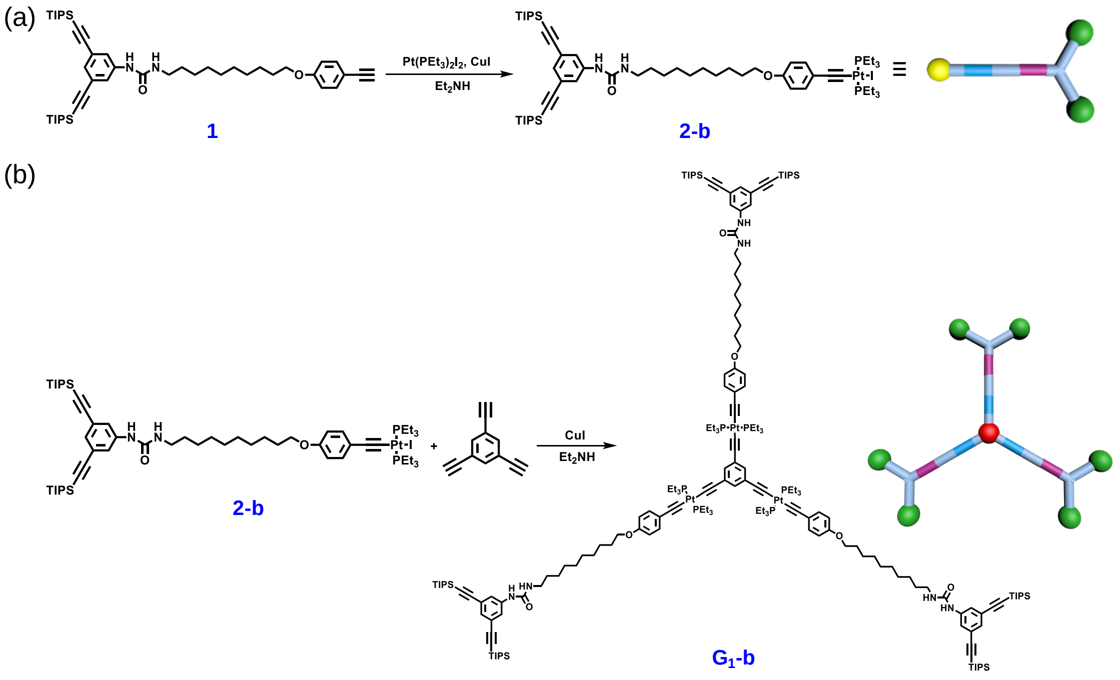

Synthesis of 2-a: A Schlenk flask was charged with 100 mg (0.135 mmol) of **S_7**, DEPillar[5]arene (723 mg, 0.812 mmol) and $Pt(PEt_3)_2I_2$ (370 mg, 0.541 mmol). The Schlenk flask was then evacuated and back-filled with N_2 three times. Next, 8 mL of the mixture solvent of anhydrous and degassed $CHCl_3$ and *i*-Pr_2NH (v/v, 2:1) was added via syringe. The resultant solution was stirred for one hour under -10 ℃. Then CuI (3 mg) was added to the mixture under an inert atmosphere and the mixture was allowed to warm to room temperature and stirred overnight. The solution was concentrated and the residue was purified by column chromatography (SiO_2; PE/DCM). A pale-yellow solid **2-a** (180 mg, 61%) was obtained. ^1H NMR (500 MHz, acetone-d_6): δ 7.25-7.27 (d, J = 10 Hz, 2H), 7.22-7.23 (t, J = 2.5 Hz, 2H), 7.03-7.04 (d, J = 5 Hz, 2H), 6.93-6.97 (d, J = 20 Hz, 2H), 6.82-6.84 (d, J = 10 Hz, 2H), 3.88-4.06 (m, 40H), 3.74 (s, 10H), 3.59-3.63 (m, 2H), 3.16-3.20 (m, 2H), 2.23-2.29 (m, 12H), 1.41-1.48 (m, 30H), 1.16-1.22 (m, 60H), 1.09-1.12 (m, 2H), 0.95-0.98 (m, 2H), 0.80-0.91 (m, 4H), 0.54-0.60 (m, 2H), 0.42-0.48 (m, 2H), 0.24-0.30 (m, 2H), 0.12-0.18 (m, 2H), -0.62, -0.64 (m, 2H), 1.10 (m, 2H). ^{31}P NMR (202 MHz, acetone-d_6): δ 10.06. ^{13}C NMR (126 MHz, acetone-d_6): δ 160.56, 158.66, 150.89, 150.72, 132.74, 129.37, 129.17, 128.33, 125.79, 119.12, 115.40, 115.31, 114.91, 107.49, 92.09, 70.05, 69.16, 64.50, 64.26, 32.18, 32.12, 31.65, 31.57, 26.20, 24.27, 19.41, 17.90, 17.76, 17.62, 16.22, 12.39, 8.99. MALDI-TOF-MS: Calculated for [**2-a** + H]$^+$: m/z = 2186.06; Found: m/z = 2187.0.

Synthesis of the first-generation model rotaxane dendrimer G_1-a: A mixture of 1, 3, 5-triethynylbenzene (2.1 mg, 0.014 mmol) and platinum-acetylide building block **2-a** (100 mg, 0.046 mmol) were added in a Schlenk flask, the Schlenk flask was then evacuated and back-filled with N_2 three times. Next, degassed diethylamine (8.0 mL) and a catalytic amount of CuI were added under an inert atmosphere. The reaction was stirred overnight at room temperature. The solvent was evaporated and the residue was purified by column chromatography (SiO_2; PE/DCM) to yield a pale-yellow solid **G_1-a** (66 mg, 75%). ^1H NMR (500 MHz, CD_2Cl_2): δ 7.05-7.29 (m, 12H), 6.89-6.95 (m, 36H), 6.79-6.80 (m, 6H), 3.84-4.01 (m, 60H), 3.73 (s, 30H), 3.49 (m, 6H), 3.16-3.20 (m, 6H), 2.20-2.23 (m, 36H), 1.38-1.45 (m, 90H), 1.23-1.29 (m, 54H), 1.17 (s, 126H), 0.73-0.93 (m, 24H), 0.23-0.36 (m, 24H), -0.58 (m, 6H), -0.96 (m, 6H). ^{31}P NMR (122 MHz, CD_2Cl_2): δ 11.47. 1^{3}C NMR (126 MHz, CD_2Cl_2): δ 159.49, 150.05, 149.96, 149.88, 132.31, 128.63, 128.57, 128.51, 127.88, 124.95, 124.91, 118.61, 118.30, 114.51, 114.34, 114.25, 106.64, 91.48, 69.19, 68.69, 63.83, 63.76, 63.66, 31.38, 30.92, 30.75, 30.00, 29.94, 29.57, 29.46, 29.39, 29.19, 25.27, 23.78, 18.85, 17.16, 17.02, 16.88, 15.69, 15.66, 15.57, 11.75, 11.71, 8.72, 8.50, 1.18. MALDI-TOF-MS: Calculated for M_r = 6329.7 Da; Found: m/z = 6334.8.

Synthesis of 2-b: A Schlenk flask was charged with 200 mg (0.266 mmol) of **1** and $Pt(PEt_3)_2I_2$ (364 mg, 0.532 mmol). The Schlenk flask was then evacuated and back-filled with N_2 three times. Next, anhydrous and degassed Et_2NH (10 mL) was added via syringe. Then CuI (5 mg) was added to the mixture under an inert atmosphere and the mixture was allowed to stirred overnight at room temperature. The solution was concentrated and the residue was purified by column chromatography (SiO_2; PE/DCM). A white solid **2-b** (302 mg, 87%) was obtained. ^{1}H NMR (300 MHz, THF-d_8): δ 7.77 (s, 1H), 7.57 (d, 2H), 7.05-7.14 (m, 3H), 6.71-6.74 (d, J = 9 Hz, 2H), 5.56-5.60 (t, J = 6 Hz, 1H), 3.88-3.92 (t, J = 6 Hz, 2H), 3.12-3.19 (m, 2H), 2.18-2.28 (m, 12H), 1.33-1.48 (m, 16H), 1.11-1.21 (m, 60H). ^{31}P NMR (122 MHz, THF-d_8): δ 9.30. ^{13}C NMR (126 MHz, THF-d_8): δ 157.97, 155.26, 142.07, 132.08, 128.13, 124.33, 121.97, 114.68, 107.66, 90.25, 68.31, 40.40, 31.12, 30.37, 30.19, 27.68, 26.83, 18.87, 17.44, 17.30, 17.16, 12.04, 8.39. MALDI-TOF-MS: Calculated for M_r = 1309.6 Da; Found: m/z = 1310.3.

Synthesis of the first-generation model plantinum-acetylide dendrimer G_1-b: A mixture of 1, 3, 5-triethynylbenzene (2.8 mg, 0.019 mmol) and platinum-acetylide building block **2-b** (80 mg, 0.061 mmol) were added in a Schlenk flask, the Schlenk flask was then evacuated and back-filled with N_2 three times. Next, degassed diethylamine (8.0 mL) and a catalytic amount of CuI were added under an inert atmosphere. The reaction was stirred overnight at room temperature. The solvent was evaporated and the residue was purified by column chromatography (SiO_2; PE/DCM) to yield a pale-yellow solid **G_1-b** (55 mg, 80%). ^{1}H NMR (500 MHz, THF-d_8): δ 7.71 (s, 3H), 7.56 (d, 6H), 7.05-7.10 (m, 9H), 6.86 (s, 3H), 6.69-6.71 (d, J = 10 Hz, 6H), 5.52-5.54 (t, J = 5 Hz, 3H), 3.88-3.91 (t, 6H), 3.14-3.18 (m, 6H), 2.16-2.19 (m, 36H), 1.34-1.50 (m, 48H), 1.20-1.26 (m, 54H), 1.14 (s, 126H). ^{31}P NMR (162 MHz, THF-d_8): δ 12.23. ^{13}C NMR (126 MHz, THF-d_8): δ 157.55, 155.29, 142.07, 132.16, 130.47, 129.21, 128.12, 124.32, 122.66, 121.96, 114.50, 108.20, 107.66, 90.23, 68.28, 40.40, 31.12, 30.39, 30.37, 30.21, 30.20, 30.13, 29.76, 27.68, 26.84, 18.88, 17.26, 17.12, 16.98, 12.04, 8.55. MALDI-TOF-MS: Calculated for M_r = 3698.2 Da; Found: m/z = 3700.5.

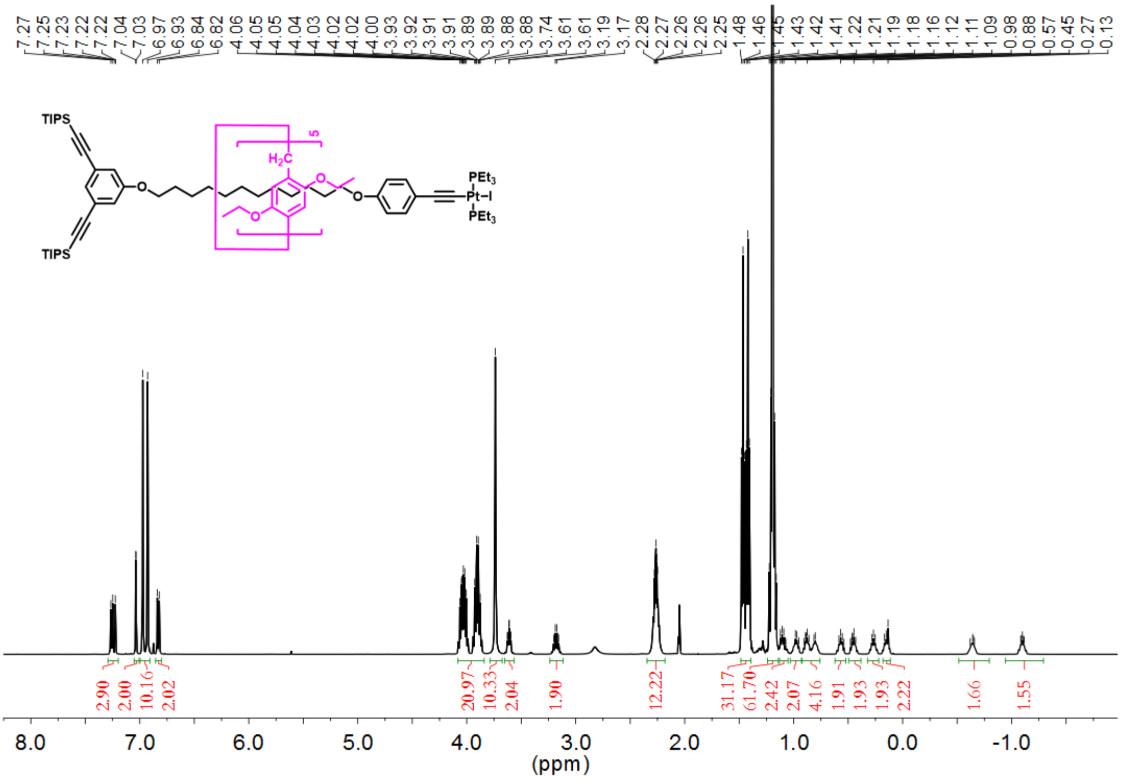

Supplementary Figure 67: ^{1}H NMR spectrum (acetone-d_6, 298 K, 500 MHz) of model complex **2-a**.

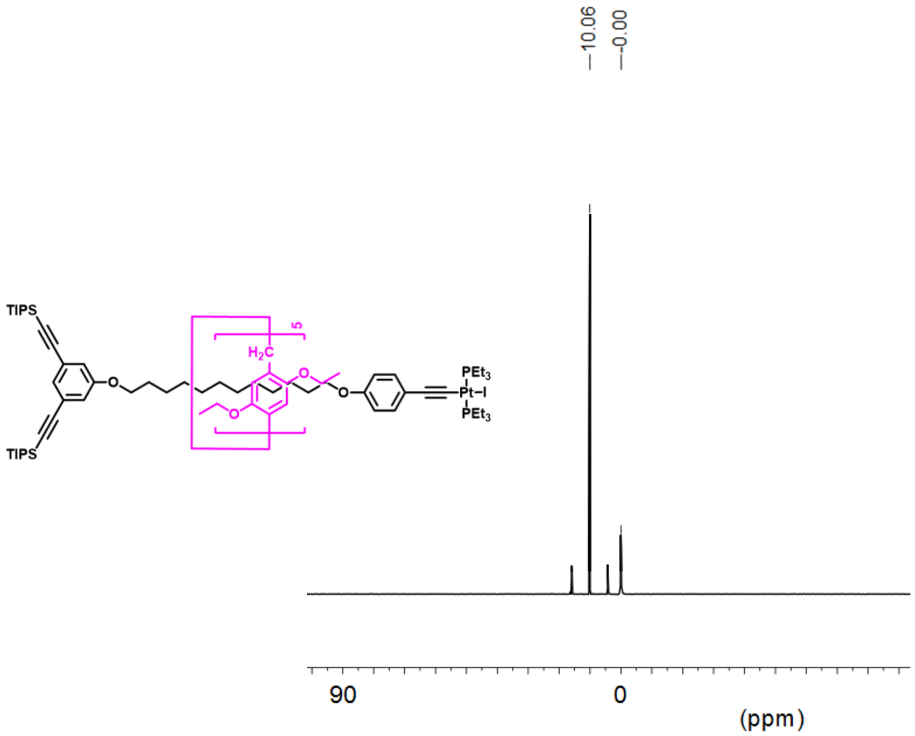

Supplementary Figure 68: ^{31}P NMR spectrum (acetone-d_6, 298 K, 202 MHz) of model complex **2-a**.

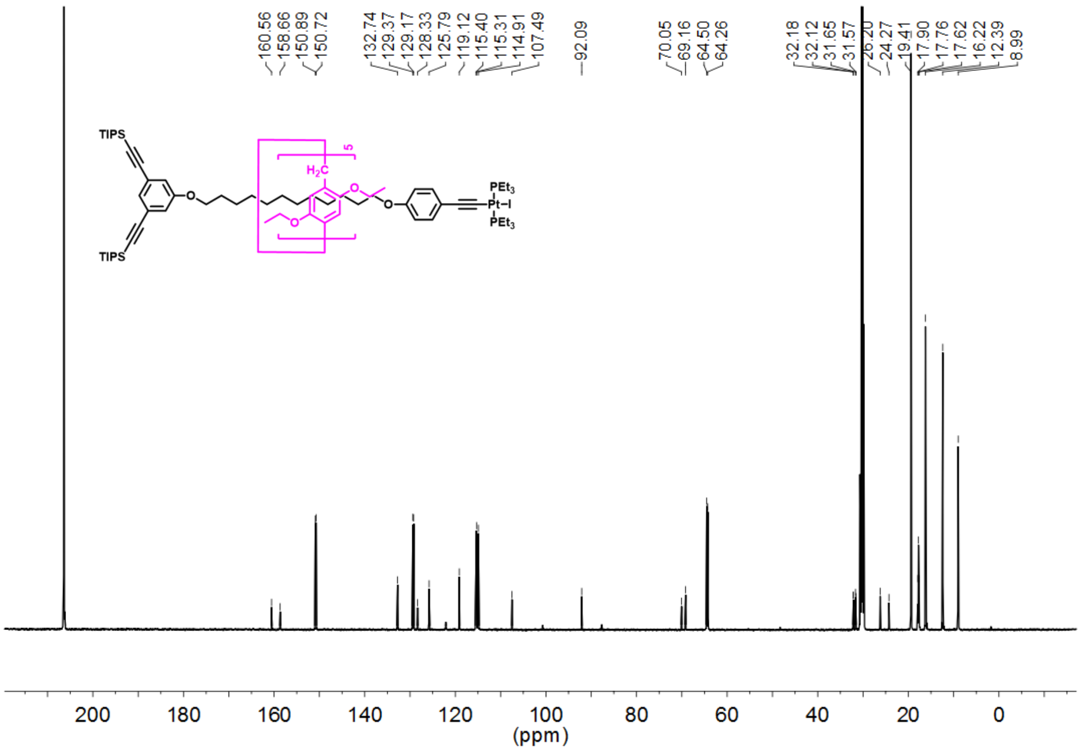

Supplementary Figure 69: ^{13}C NMR spectrum (acetone-d_6, 298 K, 126 MHz) of model complex **2-a**.

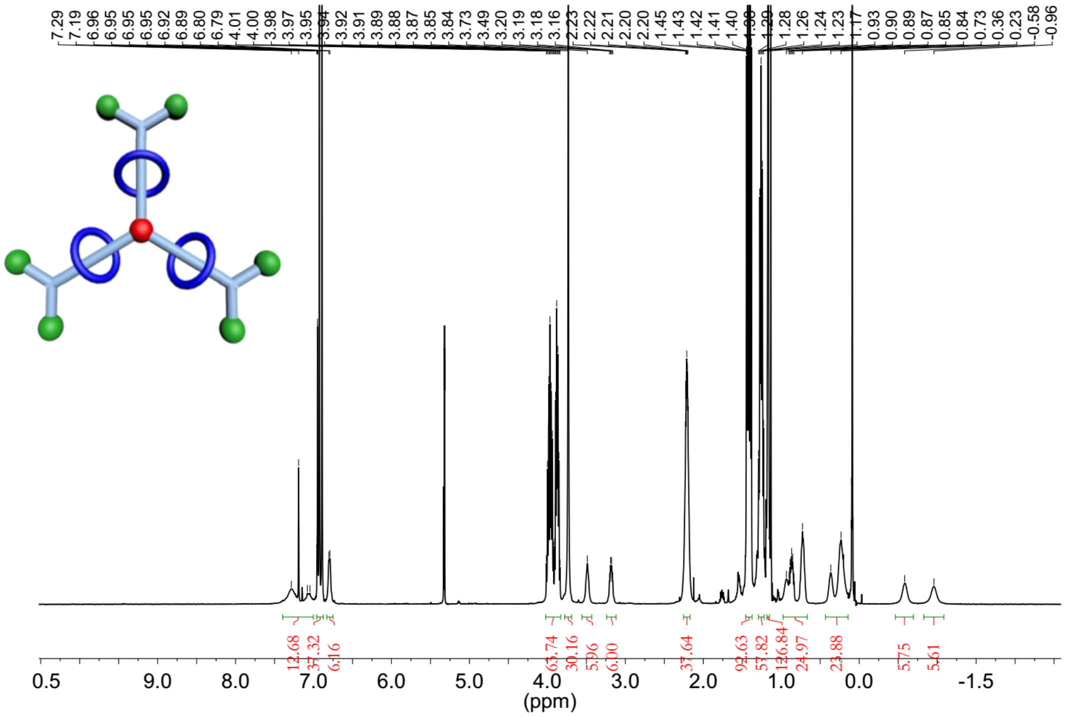

Supplementary Figure 70: ^{1}H NMR spectrum (CD_2Cl_2, 298 K, 500 MHz) of model rotaxane dendrimer **G1-a**.

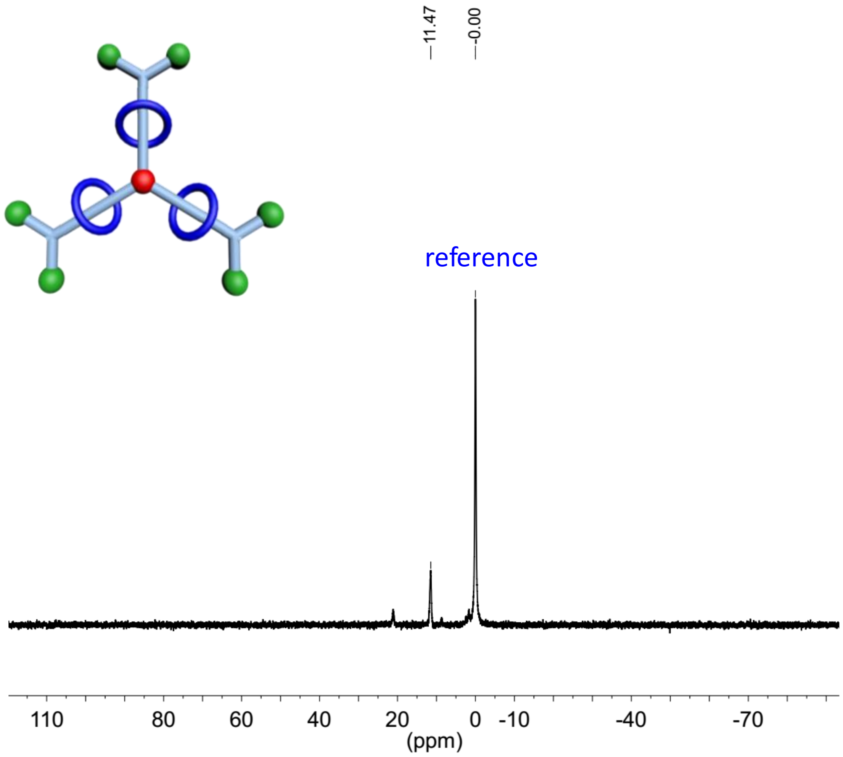

Supplementary Figure 71: ^{31}P NMR spectrum (CD_2Cl_2, 298 K, 122 MHz) of model rotaxane dendrimer **G_1-a**.

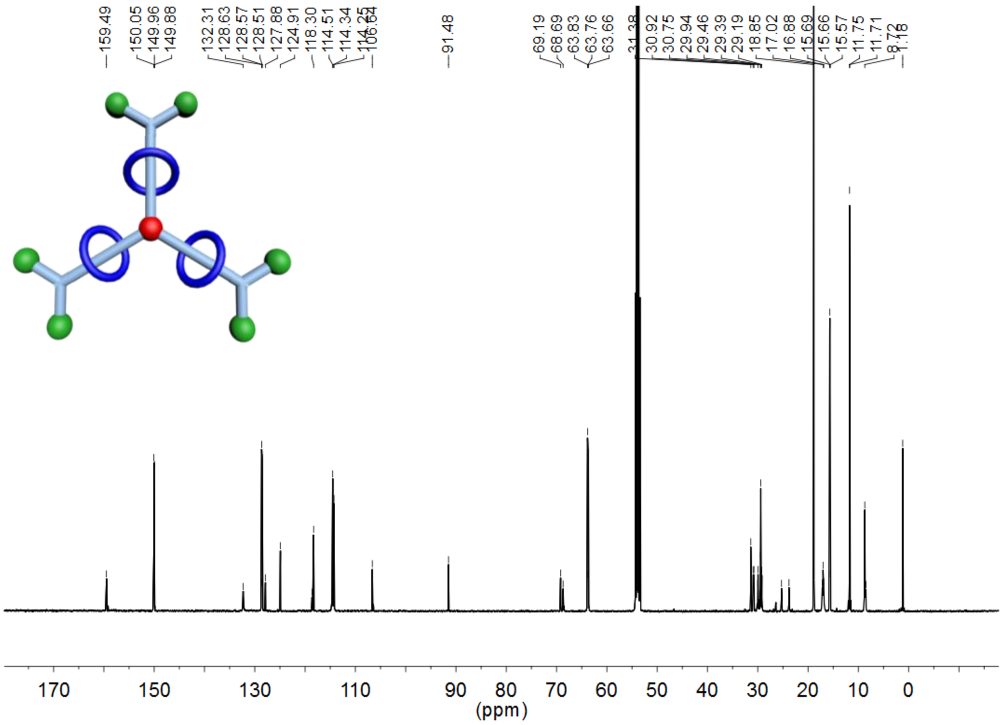

Supplementary Figure 72: ^{13}C NMR spectrum (CD_2Cl_2, 298 K, 126MHz) of model rotaxane dendrimer **G_1-a**.

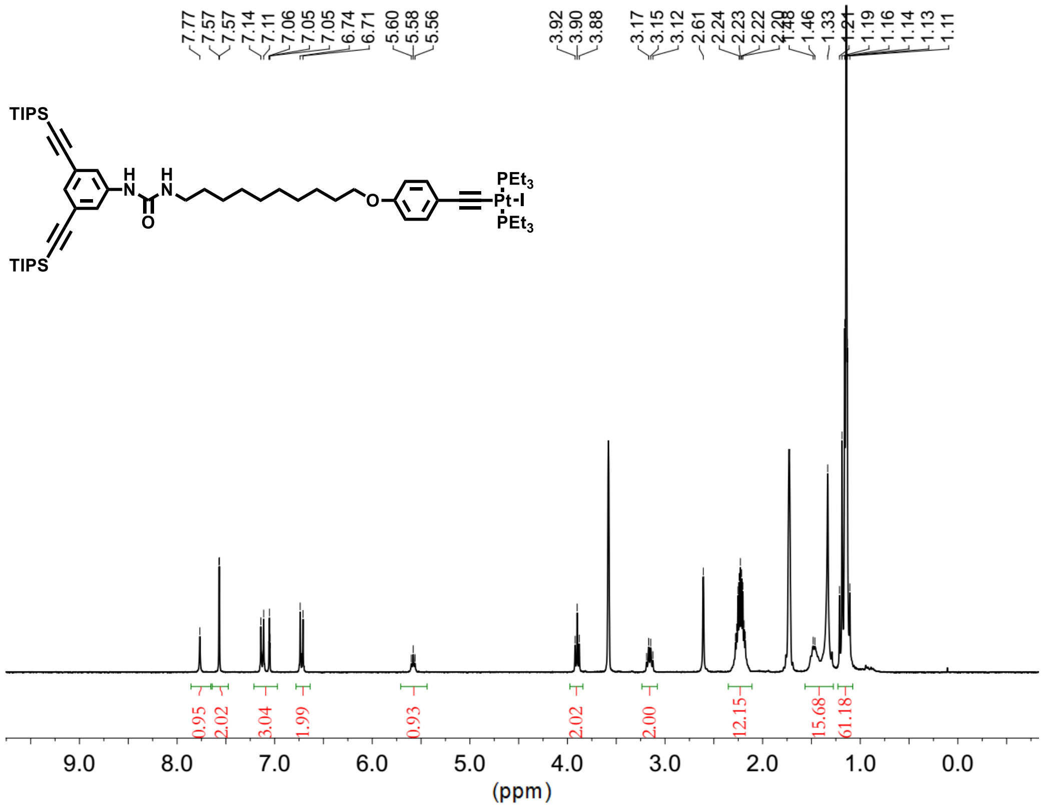

Supplementary Figure 73: ^{1}H NMR spectrum (THF-d_8, 298 K, 300 MHz) of model complex **2-b**.

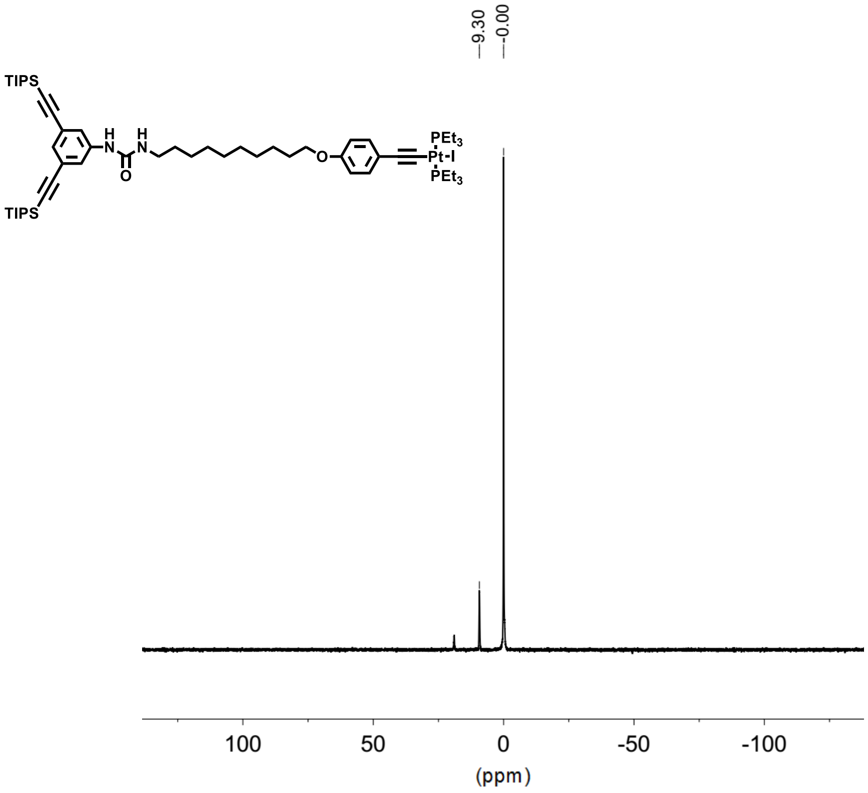

Supplementary Figure 74: ^{31}P NMR spectrum (THF-d_8, 298 K, 122 MHz) of model complex **2-b**.

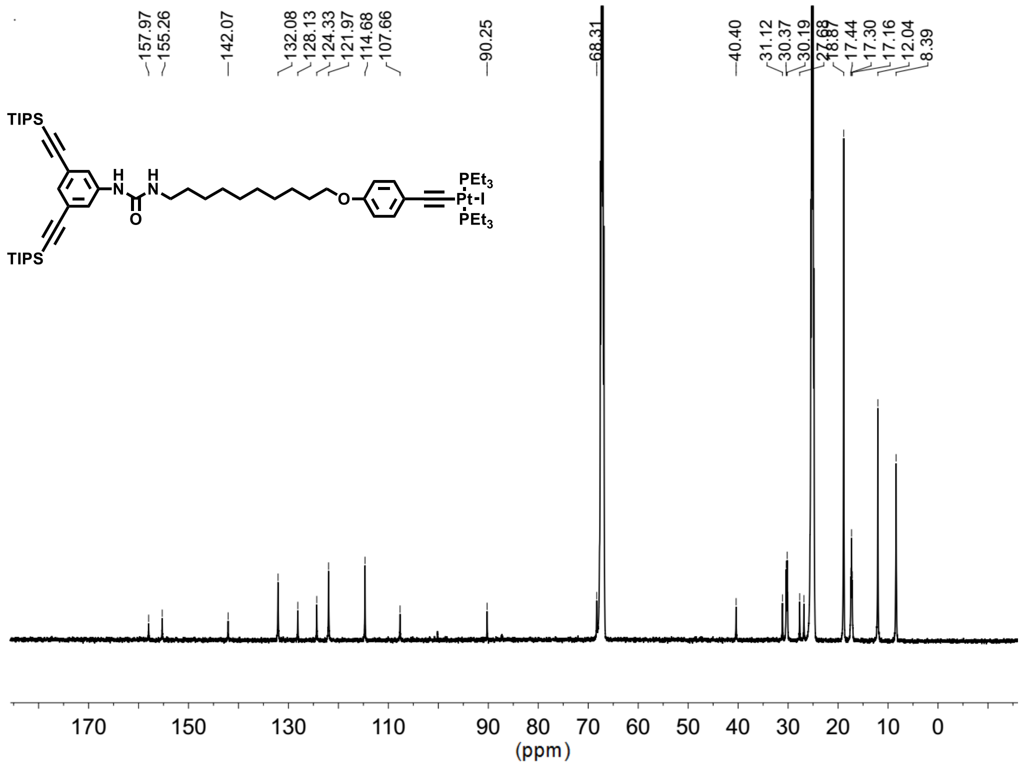

Supplementary Figure 75: ^{13}C NMR spectrum (THF-d_8, 298 K, 126 MHz) of model complex **2-b**.

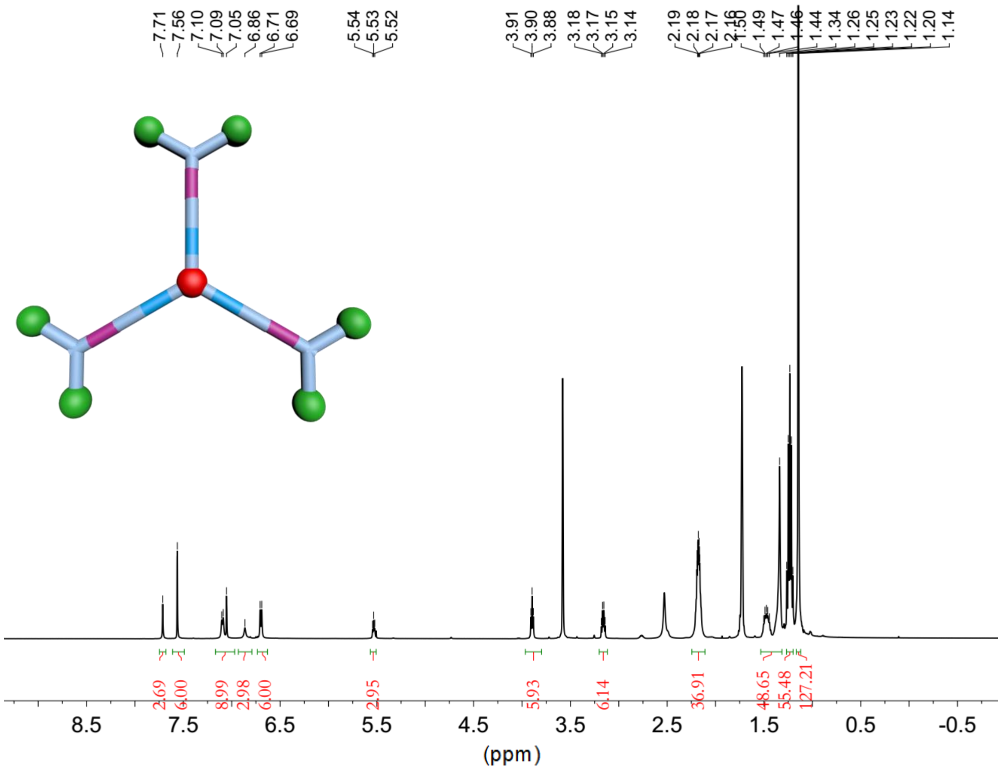

Supplementary Figure 76: ^{1}H NMR spectrum (THF-d_8, 298 K, 500 MHz) of platinum-acetylide dendrimer **G$_1$-b**.

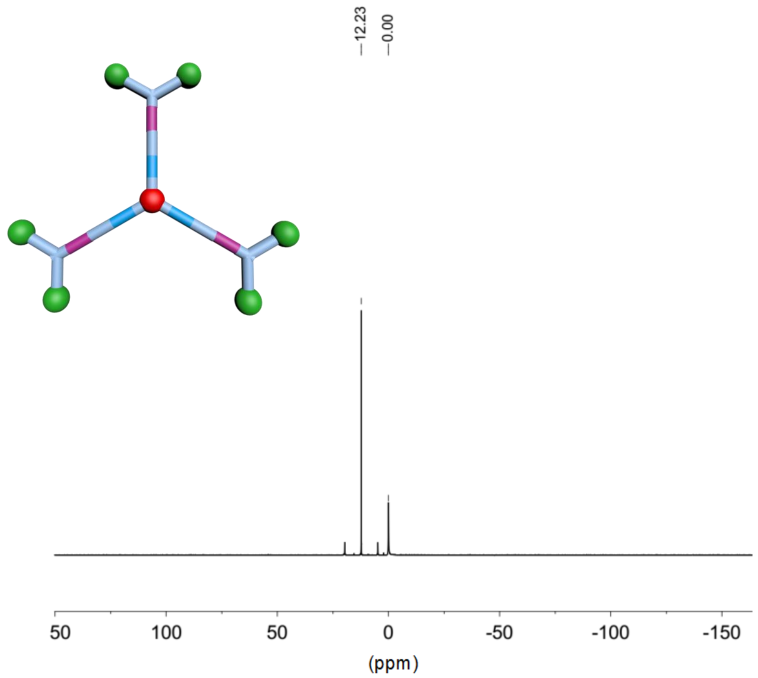

Supplementary Figure 77: ^{31}P NMR spectrum (THF-d_8, 298 K, 162 MHz) of platinum-acetylide dendrimer **G$_1$-b**.

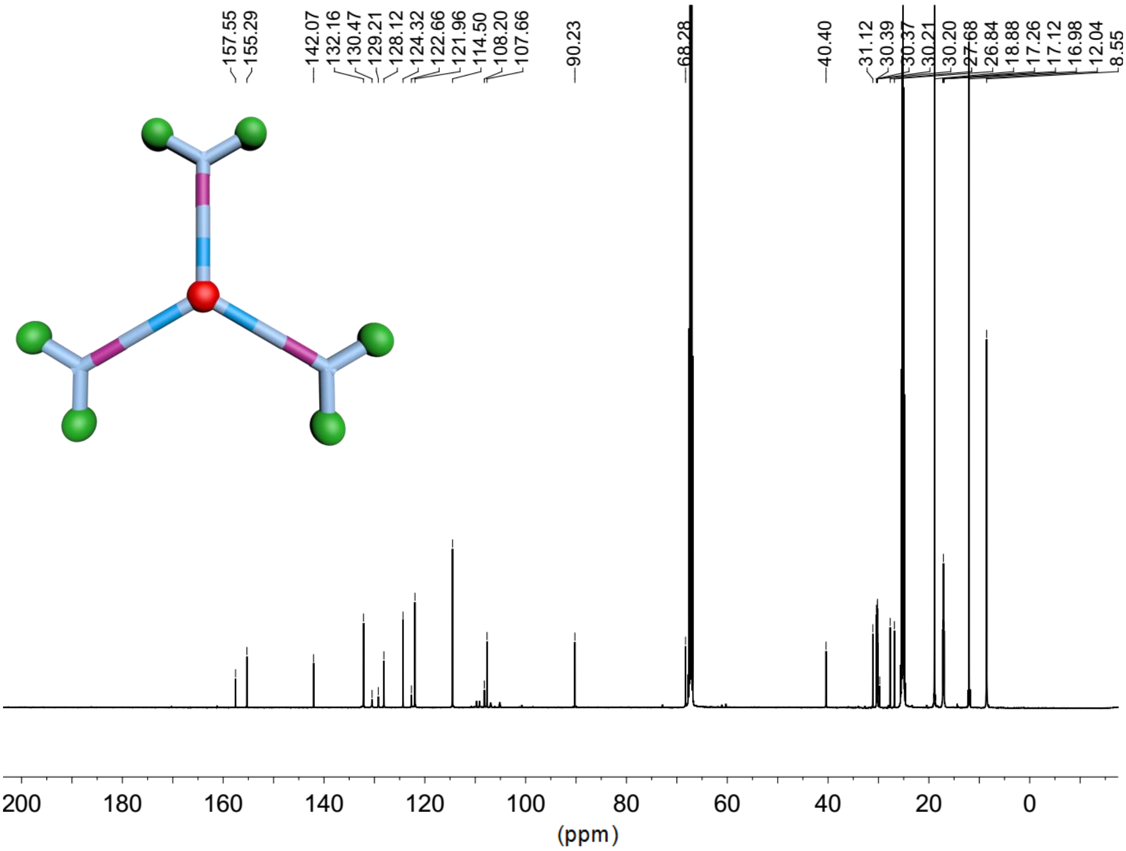

Supplementary Figure 78: ^{13}C NMR spectrum (THF-d_8, 298 K, 126MHz) of platinum-acetylide dendrimer **G$_1$-b**.

Section E. Solvent and anion-induced switching of [2]rotaxane building block 2

Supplementary Figure 79: Solvent- and anion-induced switching behavior of [2]rotaxane building block **2**.

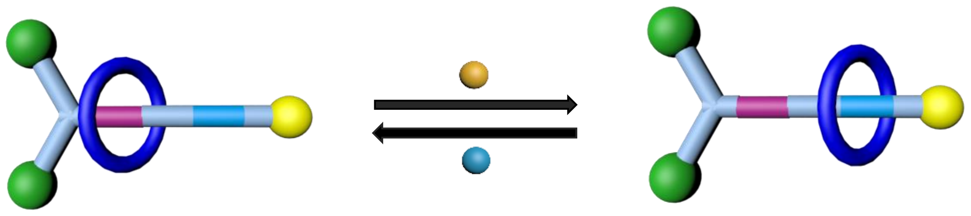

Supplementary Table 2: Solvent-induced chemical shift changes of partial protons of [2]rotaxane **2**.

Solvent-induced chemical shift changes of partial protons of **2**							
		H_3	H_4	H_{13}	H_{14}	H_{15}	H_{16}
1	THF-d_8, 400 μL	7.50	4.66	0.21	-0.62	-1.07	0.10
2	THF-d_8/DMSO-d_6, 400/2 μL	7.82	5.08	0.07	-0.89	-1.40	-0.11
3	THF-d_8/DMSO-d_6, 400/4 μL	8.00	5.32	-0.01	-1.03	-1.57	-0.22
4	THF-d_8/DMSO-d_6, 400/6 μL	8.10	5.46	-0.06	-1.11	-1.67	-0.29
5	THF-d_8/DMSO-d_6, 400/8 μL	8.17	5.55	-0.09	-1.16	-1.74	-0.33
6	THF-d_8/DMSO-d_6, 400/10 μL	8.22	5.62	-0.11	-1.20	-1.78	-0.36
7	THF-d_8/DMSO-d_6, 500/10 μL	8.15	5.52	-0.08	-1.14	-1.71	-0.32

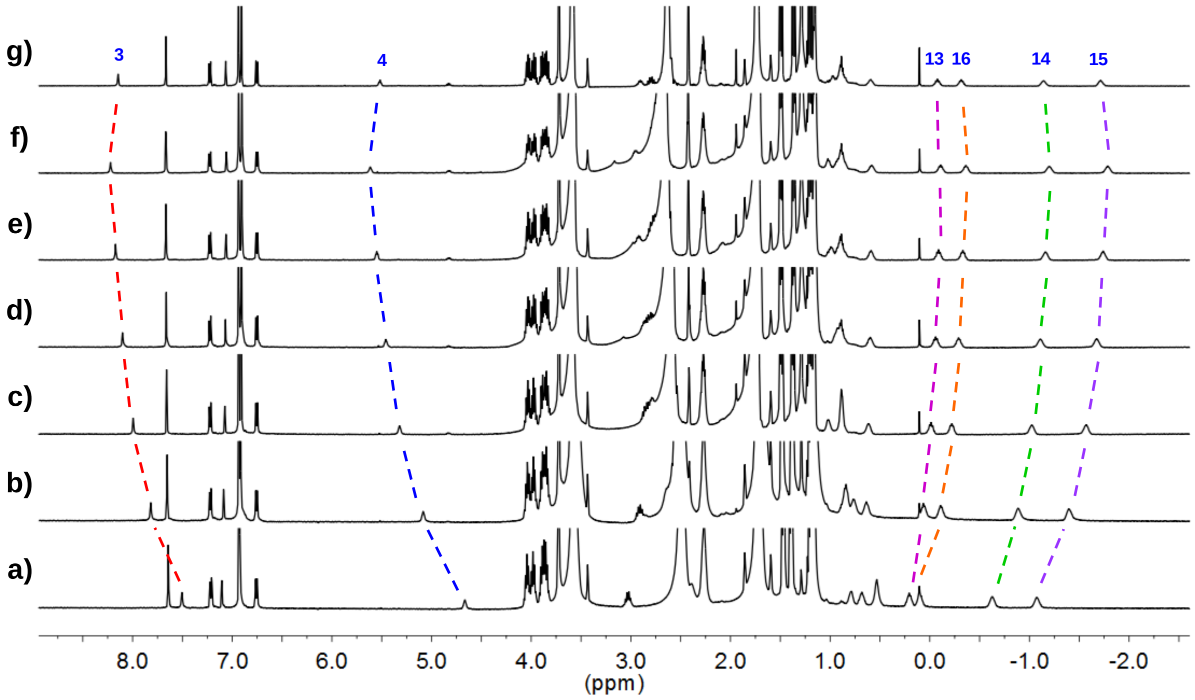

Supplementary Figure 80: ^{1}H NMR spectra (THF-d_8 (400 μL), 298 K, 500 MHz) of solvent-induced switching behavior of [2]rotaxane **2**. a) **2**; the addition of DMSO-d_6 in a): b) 2 μL; c) 4 μL; d) 6 μL; e) 8 μL; f) 10 μL; g) the addition of THF-d_8 (100 μL) in f).

Supplementary Table 3: Anion-induced chemical shift changes of partial protons of [2]rotaxane **2**.

Anion-induced chemical shift changes of partial protons of **2**							
		H_3	H_4	H_{13}	H_{14}	H_{15}	H_{16}
a	**2**	7.48	4.63	0.22	-0.60	-1.05	0.11
b	**2**+TBAA (1 eq.)	*	*	-0.04	-1.10	-1.65	-0.27
c	**2**+TBAA (2 eq.)	11.30	8.84	-0.19	-1.37	-2.00	-0.49
d	**2**+TBAA (3 eq.)	11.63	9.20	-0.23	-1.44	-2.08	-0.54
e	**2**+TBAA (4 eq.)	11.71	9.29	-0.24	-1.46	-2.11	-0.56
f	**2**+TBAA (5 eq.)	11.71	9.29	-0.24	-1.46	-2.11	-0.56
g	**2**+TBAA+$NaPF_6$ (5 eq.)	10.69	8.19	-0.15	-1.30	-1.91	-0.44
h	**2**+TBAA+$NaPF_6$ (7 eq.)	7.51	4.69	0.20	-0.64	-1.09	0..09

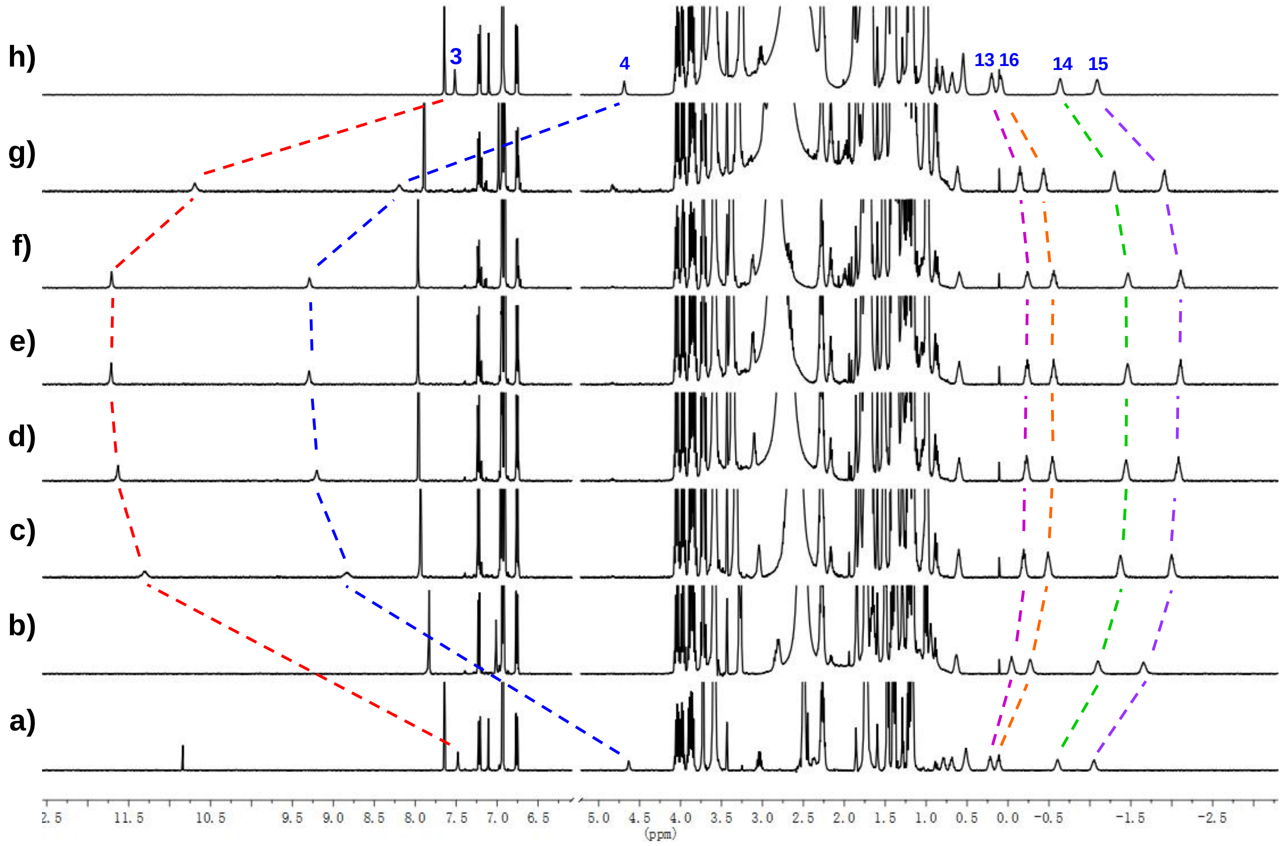

Supplementary Figure 81: ^{1}H NMR spectra (THF-d_8, 298 K, 500 MHz) of anion-induced switching motion of [2]rotaxane building block **2**. a) **2**; the mixture of **2** and TBAA: b) TBAA (1 equiv); (c) TBAA (2 equiv); d) TBAA (3 equiv); e) TBAA (4 equiv); f) TBAA (5 equiv); and the mixture obtained after adding $NaPF_6$ to the solution in f): g) $NaPF_6$ (5 equiv); h) $NaPF_6$ (7 equiv).

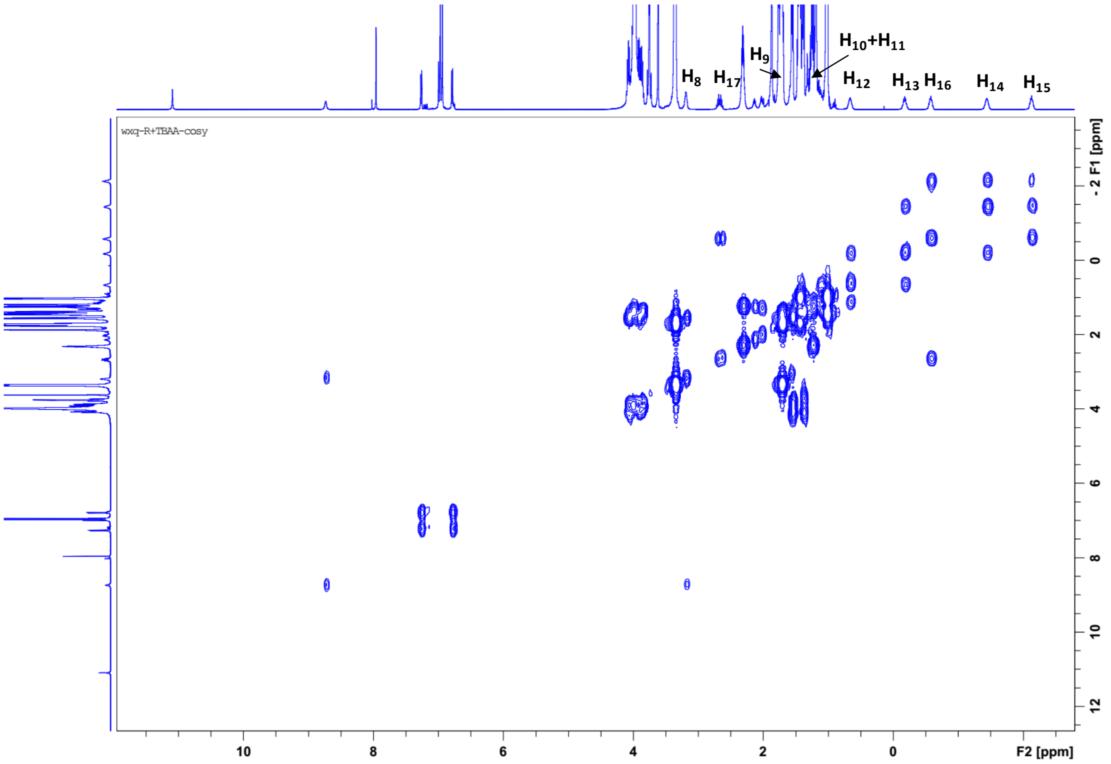

Supplementary Figure 82: 2D ^{1}H-^{1}H COSY spectrum (THF-d_8, 298 K, 500 MHz) of [2]rotaxane **2** with the addition of 5 eq. of TBAA.

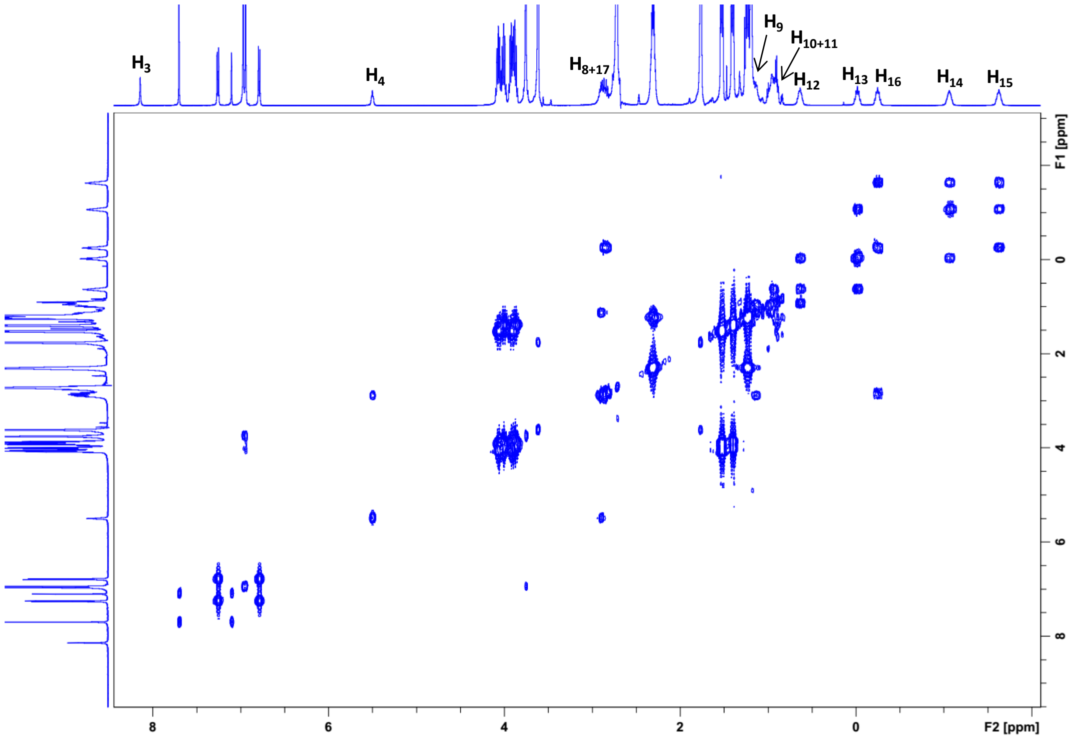

Supplementary Figure 83: 2D ^{1}H-^{1}H COSY spectrum (THF-d_8 (400 μL), 298 K, 500 MHz) of [2]rotaxane **2** with the addition of DMSO-d_6 (10 μL).

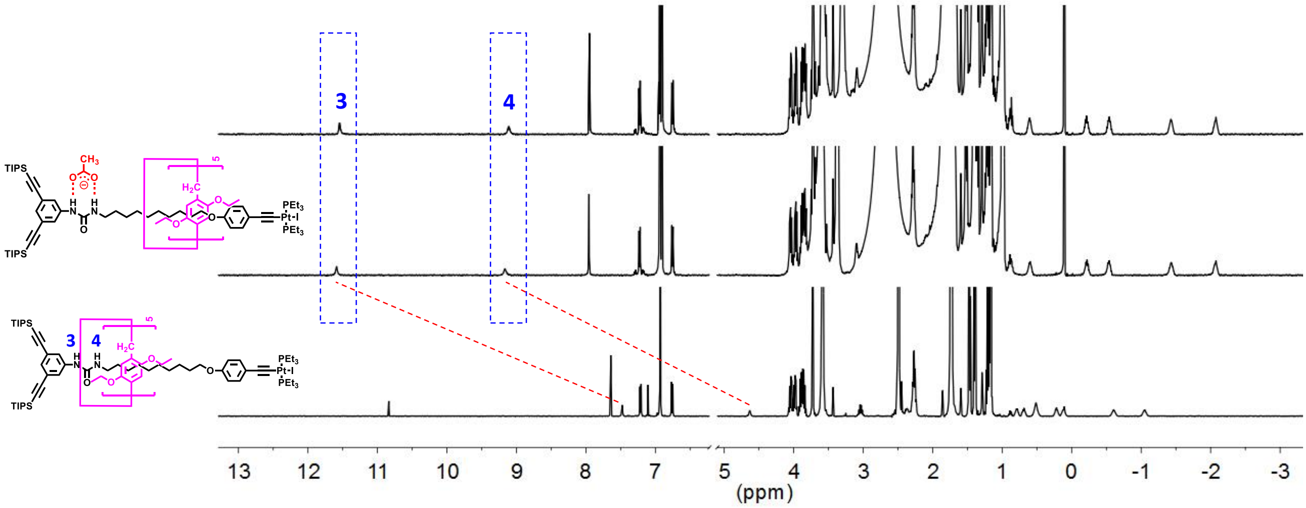

Supplementary Figure 84: ^{1}H NMR spectra (THF-d_8, 298 K, 500 MHz) of [2]rotaxane **2** *(bottom)*; **2** with the addition of TBAA (5 eq.) *(middle)*; the mixture obtained after adding TBAPF6 to the solution in middle *(top)*. (A control experiment was carried out to rule out the possible influence of PF_6^- anion to the anion-induced switching in this study. Upon the addition of 5.0 eq. $TBAPF_6$ into the THF-d_8 solution of [2]rotaxane **2**, no obvious change in ^{1}H NMR spectrum was observed, thus indicating the weak binding ability of PF_6^- towards the urea moiety compared with the acetate anions.)

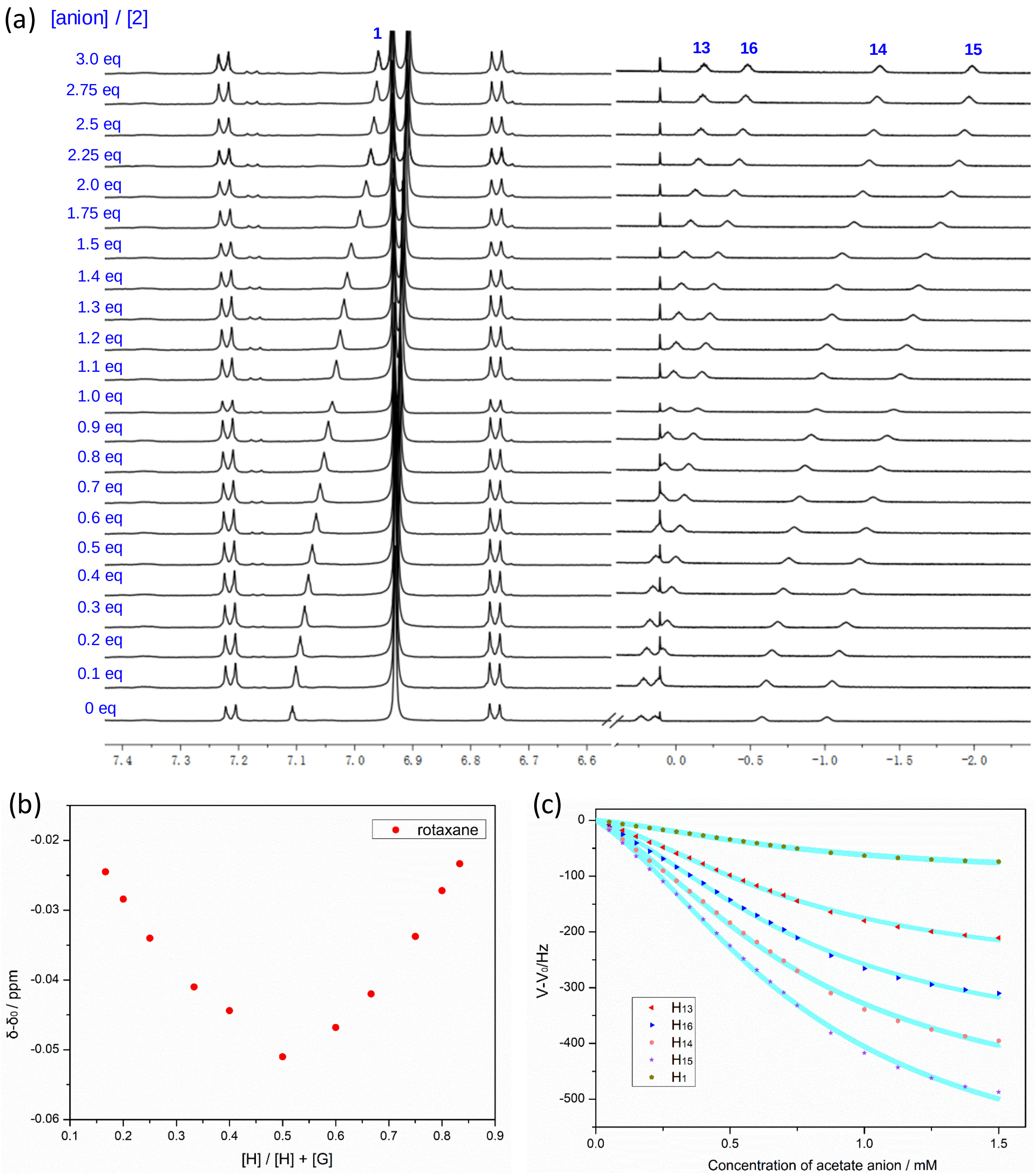

Supplementary Figure 85: (a) The expanded region of the ^{1}H NMR spectra (THF-d_8, 298 K, 500 MHz) of [2]rotaxane **2** (Host) with the addition of acetate anion (Guest); (b) Job plot for [2]rotaxane **2**-acetate anion complex in THF-d_8 ([**2**] + [anion] = 1 mM); (c) The ^{1}H NMR titration isotherm of [2]rotaxane **2** with the addition of acetate anion (TBAA) recorded at 500 MHz in THF-d_8 at 298 K.(⬟, ◀, ▶, ●, ★indicate the change in chemical shift of the proton signals corresponding to H_1, H_{13}, H_{16}, H_{14}, H_{15}, respectively, on rotaxane **2** in (a)). By using the Hyperquad 2003 program, the binding constant was calculated to be log K = 3.57 ±0.2.

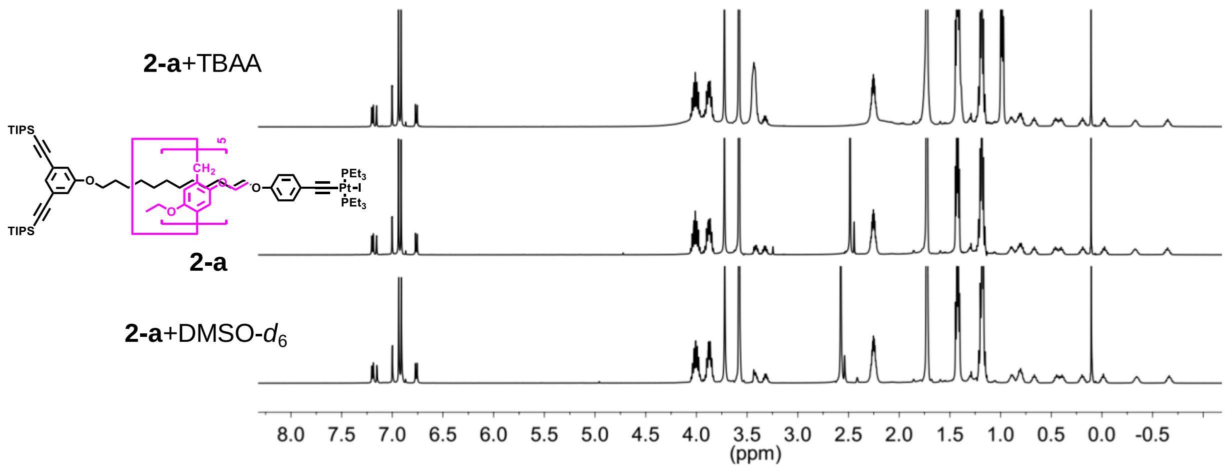

Supplementary Figure 86: ^{1}H NMR spectra (THF-d_8, 298 K, 500 MHz) of model complex **2-a** with the addition of DMSO-d_6 (10 μL) *(bottom)*; **2-a** *(middle)*; **2-a** with the addition of TBAA (5 eq.) *(top)*.

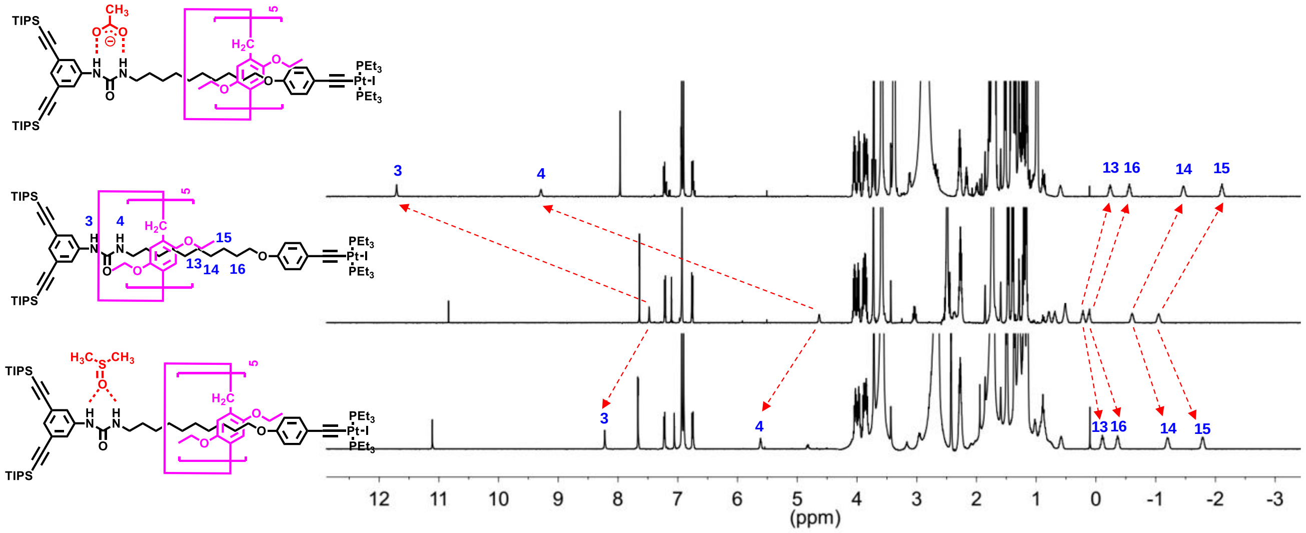

Supplementary Figure 87: ^{1}H NMR spectra (THF-d_8, 298 K, 500 MHz) of [2]rotaxane **2** with the addition of DMSO-d_6 (10 μL) *(botom)*; [2]rotaxane **2** *(middle)*; [2]rotaxane **2** with the addition of TBAA (5.0 eq.) *(top)*.

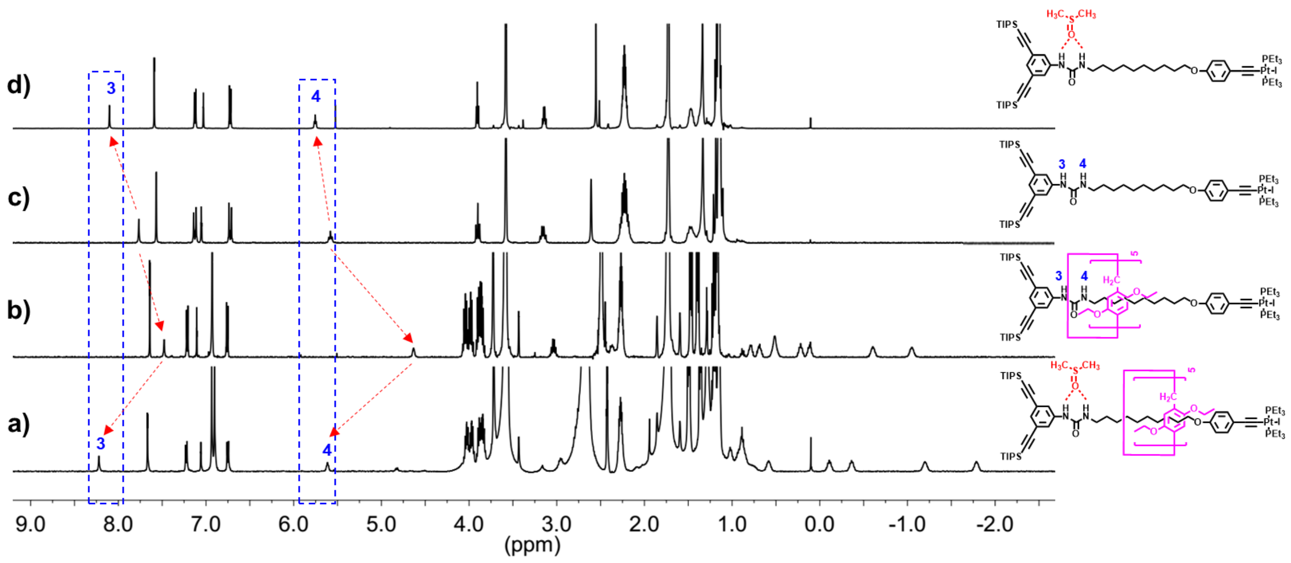

Supplementary Figure 88: ^{1}H NMR spectra (THF-d_8, 298 K, 500 MHz) of a) [2]rotaxane **2** with the addition of DMSO-d_6 (10 μL); b) [2]rotaxane **2**; c) model complex **2-b**; d) model complex **2-b** with the addition of DMSO-d_6 (10 μL);

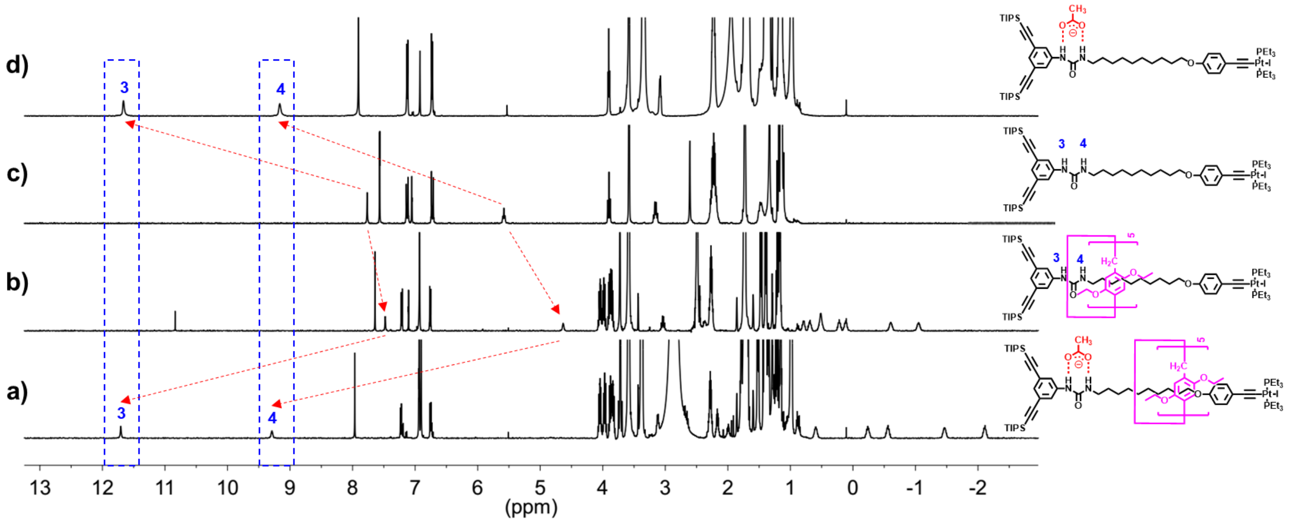

Supplementary Figure 89: ^{1}H NMR spectra (THF-d_8, 298 K, 500 MHz) of a) [2]rotaxane **2** with the addition of TBAA (5.0 eq.); b) [2]rotaxane **2**; c) model complex **2-b**; d) model complex **2-b** with the addition of TBAA (5.0 eq.).

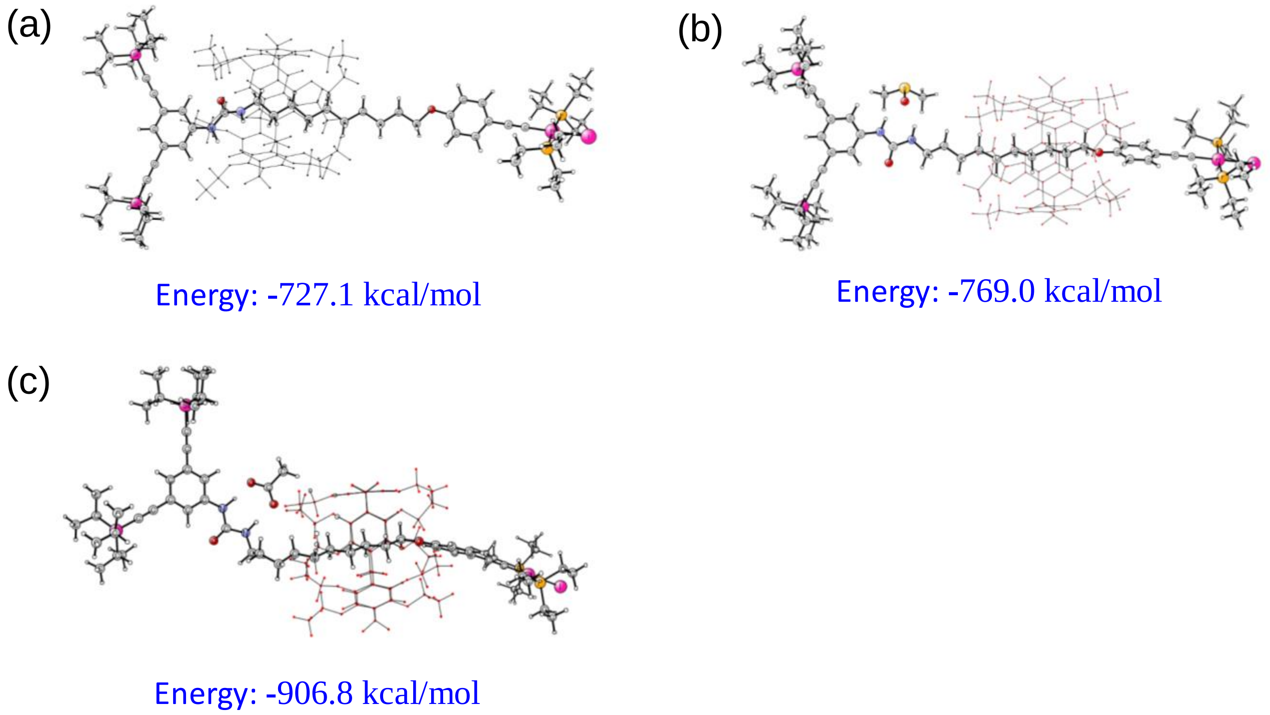

Supplementary Figure 90: Theoretically calculated structures of (a) [2]rotaxane **2**; (b) solvent-induced and (c) anion-induced switching motion of DEP5 ring in [2]rotaxane **2**.

Section F. Solvent- and anion-induced switching of the rotaxane-branched dendrimers.

Supplementary Figure 91: Solvent- and anion-induced switching behavior of rotaxane-branched dendrimer $\mathbf{G_1}$.

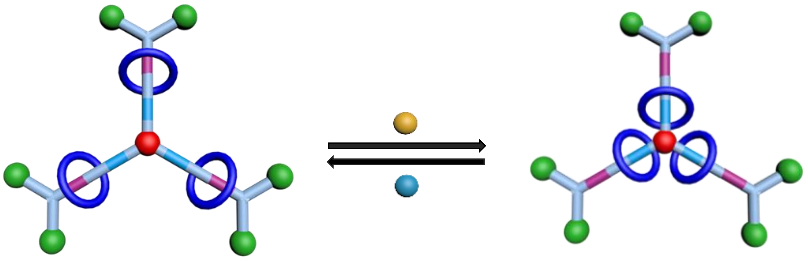

Supplementary Table 4: Solvent-induced chemical shift changes of partial protons of rotaxane-branched dendrimer $\mathbf{G_1}$.

Solvent-induced chemical shift changes of partial protons of $\mathbf{G_1}$							
		H_3	H_4	H_{13}	H_{14}	H_{15}	H_{16}
1	THF-d_8, 400 μL	7.51	4.67	0.20	-0.63	-1.06	0.11
2	THF-d_8/DMSO-d_6, 400/2 μL	7.74	4.97	0.09	-0.82	-1.30	-0.04
3	THF-d_8/DMSO-d_6, 400/4 μL	7.95	5.25	0.00	-1.00	-1.52	-0.18
4	THF-d_8/DMSO-d_6, 400/6 μL	8.05	5.39	-0.05	-1.08	-1.62	-0.24
5	THF-d_8/DMSO-d_6, 400/8 μL	8.12	5.48	-0.08	-1.13	-1.68	-0.29
6	THF-d_8/DMSO-d_6, 400/10 μL	8.18	5.55	-0.10	-1.17	-1.74	-0.32
7	THF-d_8/DMSO-d_6, 500/10 μL	8.08	5.43	-0.06	-1.09	-1.64	-0.25

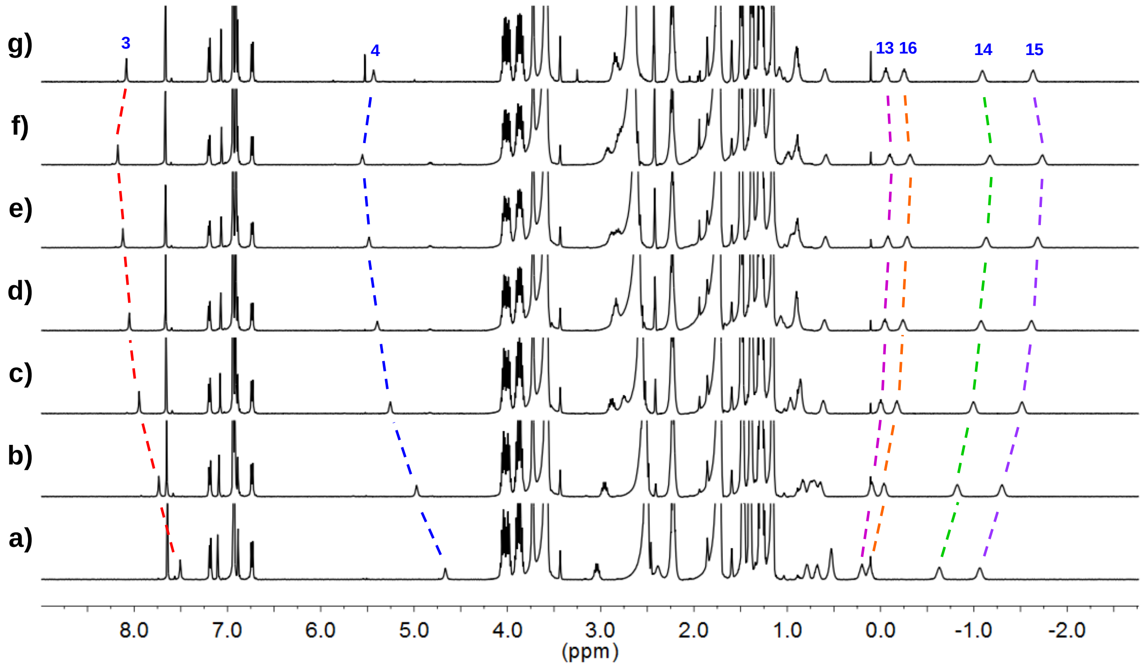

Supplementary Figure 92: ^{1}H NMR spectra (THF-d_8 (400 μL), 298 K, 500 MHz) of solvent-induced switching behavior of rotaxane-branched dendrimer $\mathbf{G_1}$. a) $\mathbf{G_1}$; the addition of DMSO-d_6 in a): b) 2 μL; c) 4 μL; d) 6 μL; e) 8 μL; f) 10 μL; g) the addition of THF-d_8 (100 μL) in f).

Supplementary Table 5: Anion-induced chemical shift changes of partial protons of rotaxane-branched dendrimer **G_1**.

Anion-induced chemical shift changes of partial protons of **G_1**							
		H_3	H_4	H_{13}	H_{14}	H_{15}	H_{16}
a	**G_1**	7.48	4.63	0.21	-0.61	-1.05	0.13
b	**G_1**+TBAA (1 eq.)	10.26	7.89	-0.11	-1.20	-1.78	-0.34
c	**G_1**+TBAA (2 eq.)	11.35	8.90	-0.22	-1.43	-2.05	-0.51
d	**G_1**+TBAA (3 eq.)	11.58	9.16	-0.24	-1.47	-2.11	-0.55
e	**G_1**+TBAA (4 eq.)	11.64	9.22	-0.25	-1.49	-2.12	-0.56
f	**G_1**+TBAA (5 eq.)	11.65	9.23	-0.25	-1.49	-2.13	-0.56
g	**G_1**+TBAA+$NaPF_6$ (5 eq.)	10.67	8.20	-0.18	-1.37	-1.99	-0.48
h	**G_1**+TBAA+$NaPF_6$ (7 eq.)	7.54	4.72	*	-0.68	-1.10	*

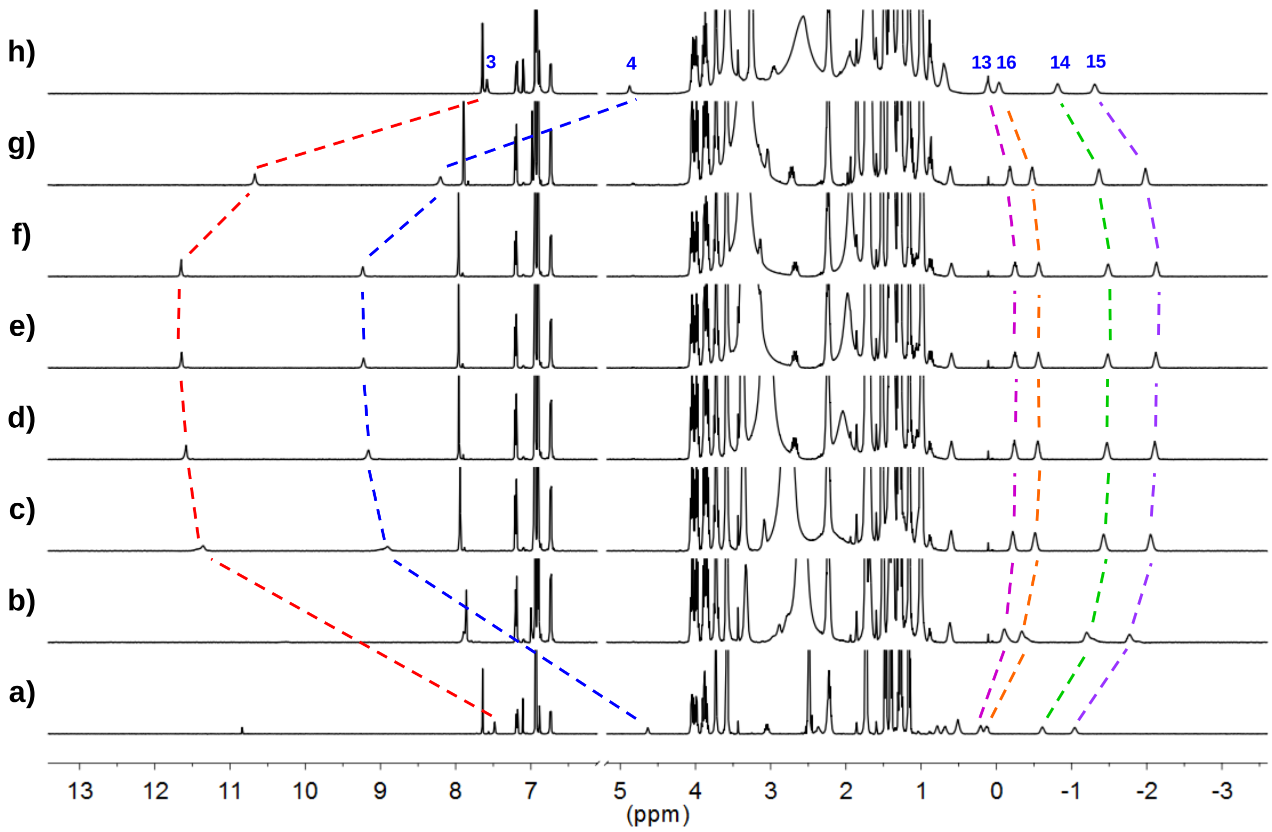

Supplementary Figure 93: ^{1}H NMR spectra (THF-d_8, 298 K, 500 MHz) of anion-induced switching behavior of rotaxane-branched dendrimer **G_1**. a) **G_1**; the mixture of **G_1** and TBAA, for each rotaxane unit: b) TBAA (1 equiv); c) TBAA (2 equiv); d) TBAA (3 equiv); e) TBAA (4 equiv); f) TBAA (5 equiv); and the mixture obtained after adding $NaPF_6$ to the solution in f), for each rotaxane unit: g) $NaPF_6$ (5 equiv); h) $NaPF_6$ (7 equiv).

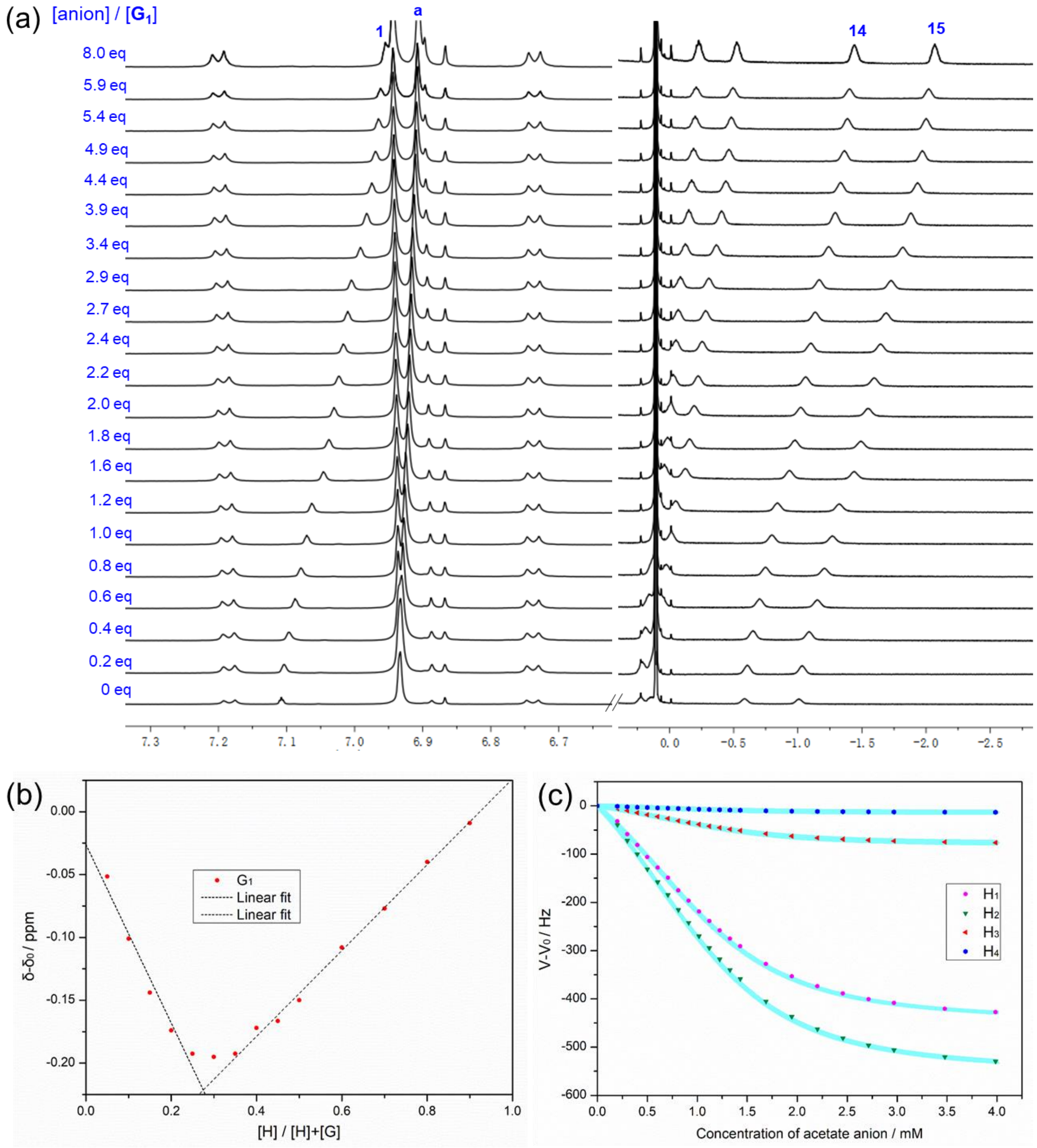

Supplementary Figure 94: (a) The expanded region of the ^{1}H NMR spectra (THF-d_8, 298 K, 500 MHz) of rotaxane-branched dendrimer **G1** (Host) with the addition of acetate anion (Guest); (b) Job plot for rotaxane-branched dendrimer **G1**-acetate anion complex in THF-d_8 ([**G1**] + [anion] = 4 mM); (c) The ^{1}H NMR titration isotherm of rotaxane-branched dendrimer **G1** with the addition of acetate anion (TBAA) recorded at 500 MHz in THF-d_8 at 298 K. By using the Hyperquad 2003 program, the binding constants for acetate anion were calculated to be log K_1 = 4.19 ±0.2, log K_2 = 3.43 ±0.2, log K_3 = 3.21 ±0.2

The calculation details were listed below:

$[H] + [G] \rightarrow [HG]$ $\log K_1 = 4.19$

$[H] + 2[G] \rightarrow [HG_2]$ $\log K_2 = 7.62$

$[H] + 3[G] \rightarrow [HG_3]$ $\log K_3 = 10.83$

According to the equilibrium constants K, the three binding constants K' were calculated, the details are shown below:

$[H] + [G] \rightarrow [HG]$ (K_1')

$[HG] + [G] \rightarrow [HG_2]$ (K_2')

$[HG_2] + [G] \rightarrow [HG_3]$ (K_3')

$K_1' = K_1 = [HG] / ([H] \{G])$

Thus, $\log K_1' = \log K_1 = 4.19$

$K_2' = [HG_2] / ([HG] \{G]) = (K_2 \{H] \{G]^2) / (K_1 \{H] \{G] \{G]) = K_2 / K_1$

Thus, $\log K_2' = \log K_2 - \log K_1 = 7.62 - 4.19 = 3.43$

$K_3' = [HG_3] / ([HG_2] \{G]) = (K_3 \{H] \{G]^3) / (K_2 \{H] \{G]^3) = K_3 / K_2$

Thus, $\log K_3' = \log K_3 - \log K_2 = 10.83 – 7.62 = 3.21$

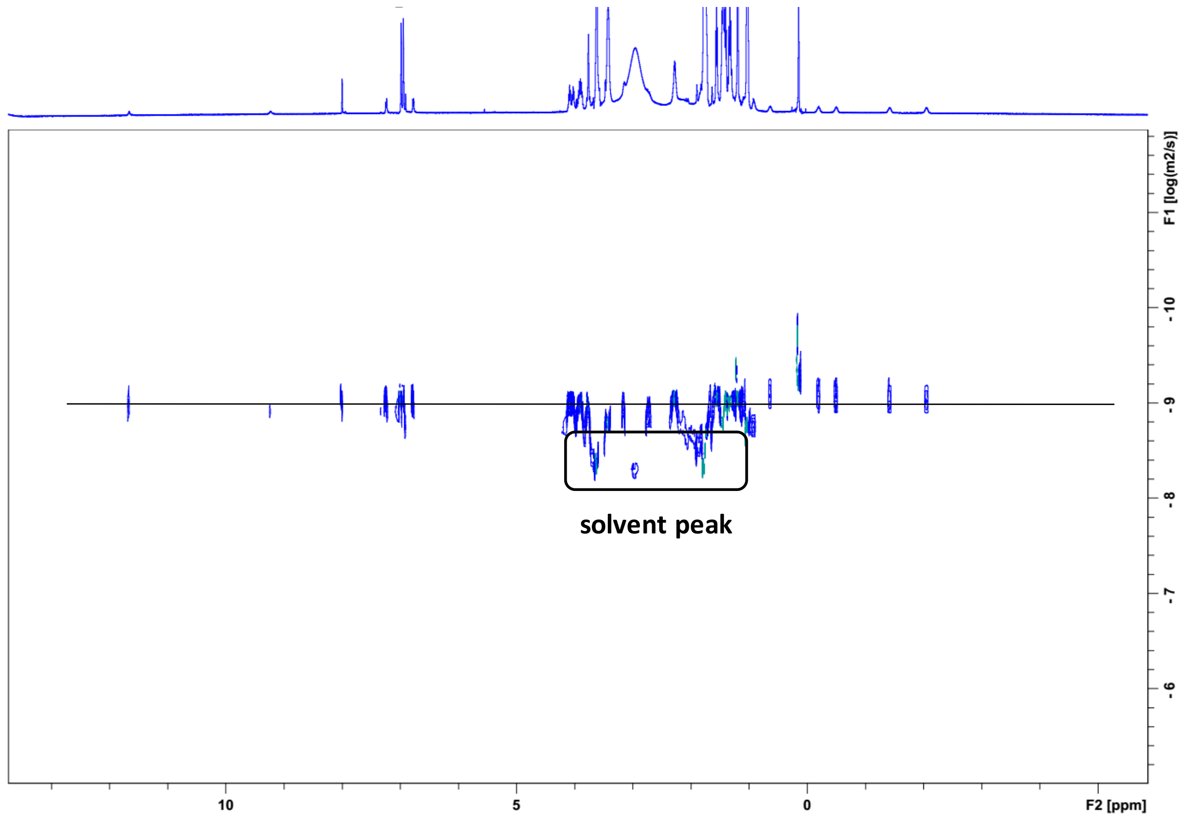

Supplementary Figure 95: 2-D DOSY spectrum (THF-d_8, 298 K, 500 MHz) of rotaxane-branched dendrimer $\mathbf{G_1}$ with the addition of TBAA (5 equiv. for each urea unit).

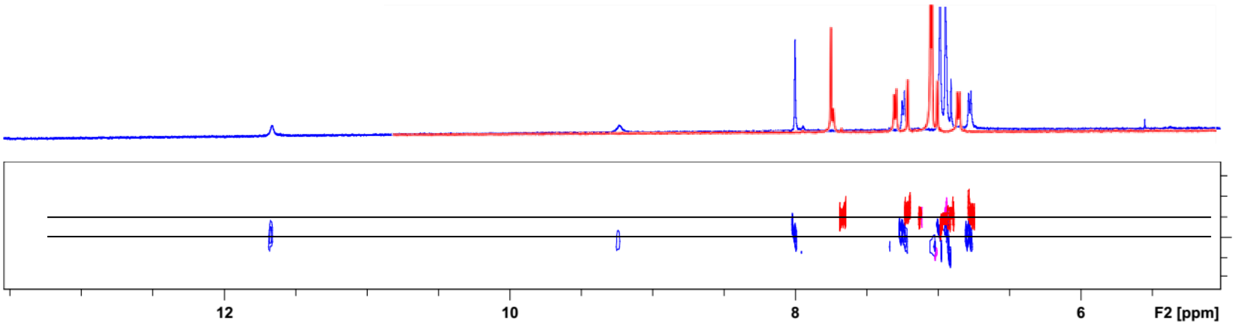

Supplementary Figure 96: Stacked DOSY spectra (THF-d_8, 298 K, 500 MHz) of rotaxane-branched dendrimer $\mathbf{G_1}$ (red) and $\mathbf{G_1}$ with the addition of TBAA (5 eq. for each urea unit) (blue).

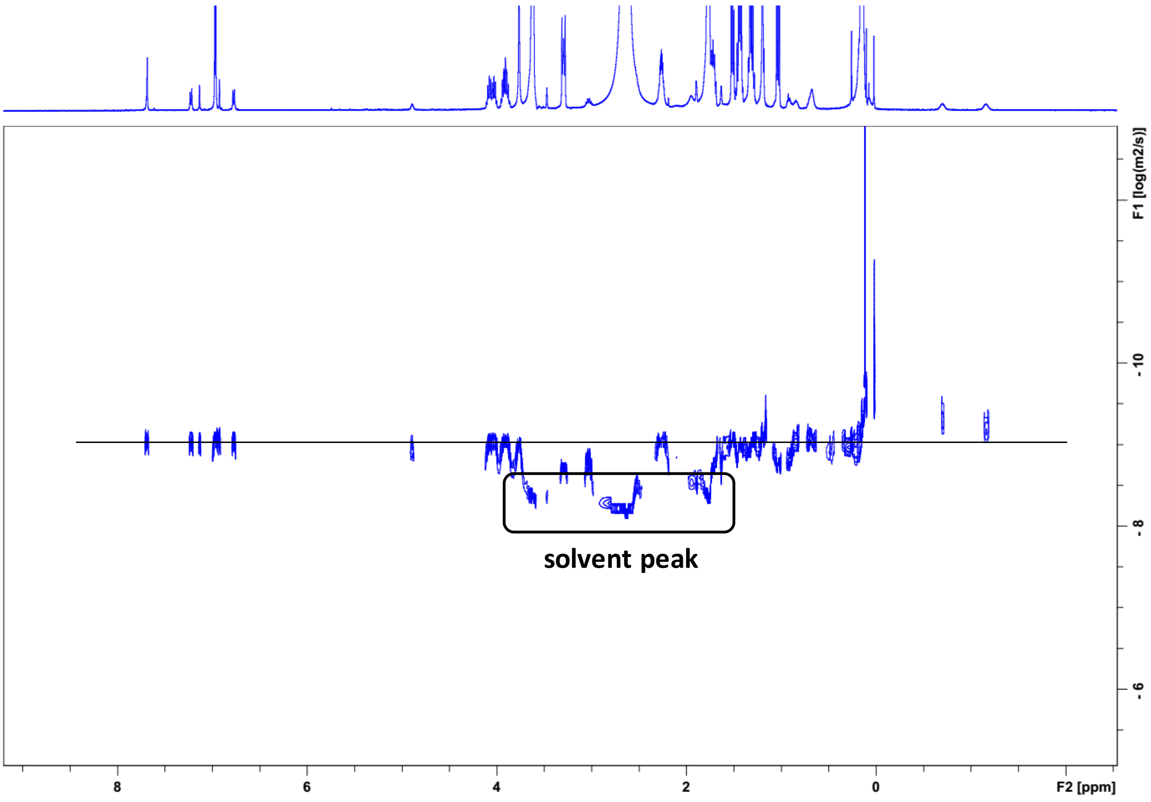

Supplementary Figure 97: 2-D DOSY spectrum (THF-d_8, 298 K, 500 MHz) of the mixture after adding $NaPF_6$ into the solution of rotaxane-branched dendrimer **G1** and TBAA.

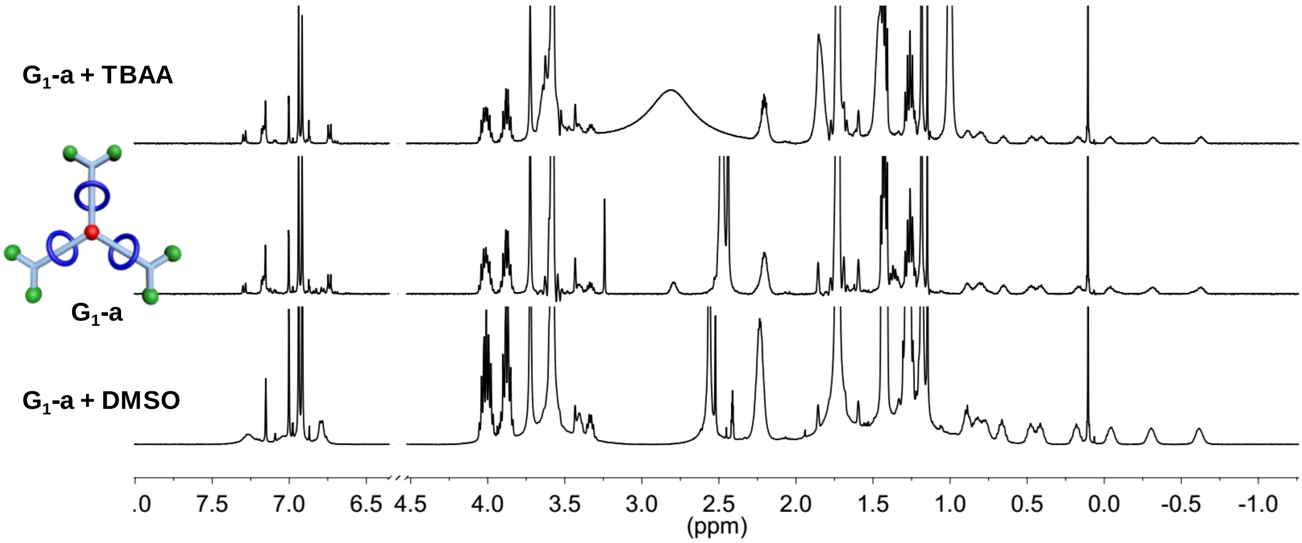

Supplementary Figure 98: ^{1}H NMR spectra (THF-d_8, 298 K, 500 MHz) of **G1-a** with the addition of DMSO-d_6 (10 μL) *(bottom)*; model dendrimer **G1-a** *(middle)*; **G1-a** with the addition of 15.0 eq. of TBAA *(top)*.

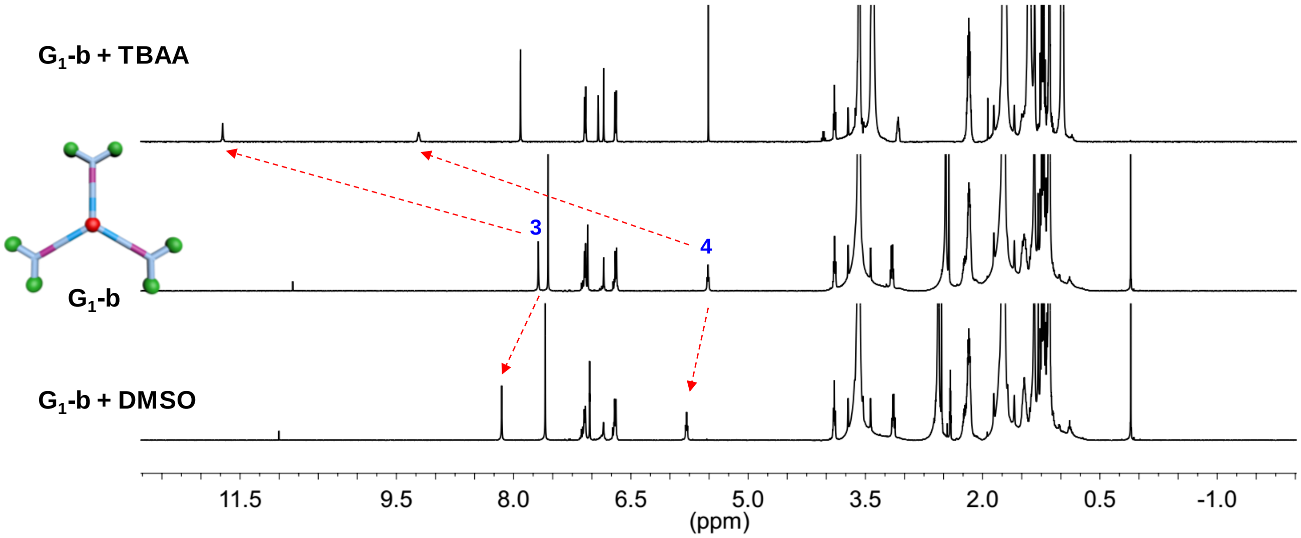

Supplementary Figure 99: ^{1}H NMR spectra (THF-d_8, 298 K, 500 MHz) of **G$_1$-b** with the addition of DMSO-d_6 (10 µL) *(bottom)*; model dendrimer **G$_1$-b** *(middle)*; **G$_1$-b** with the addition of 15.0 eq. of TBAA *(top)*.

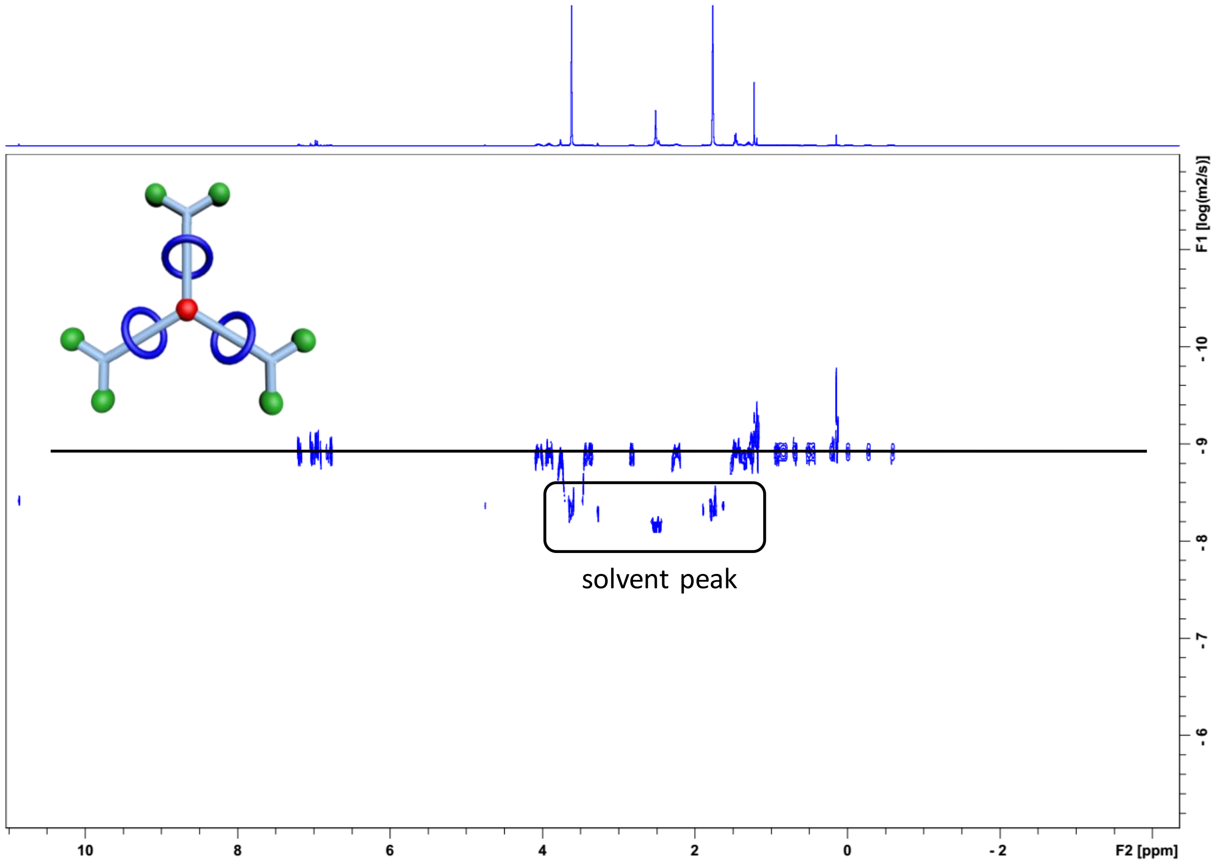

Supplementary Figure 100: 2-D DOSY spectrum (THF-d_8, 298 K, 500 MHz) of model rotaxane dendrimer **G$_1$-a**.

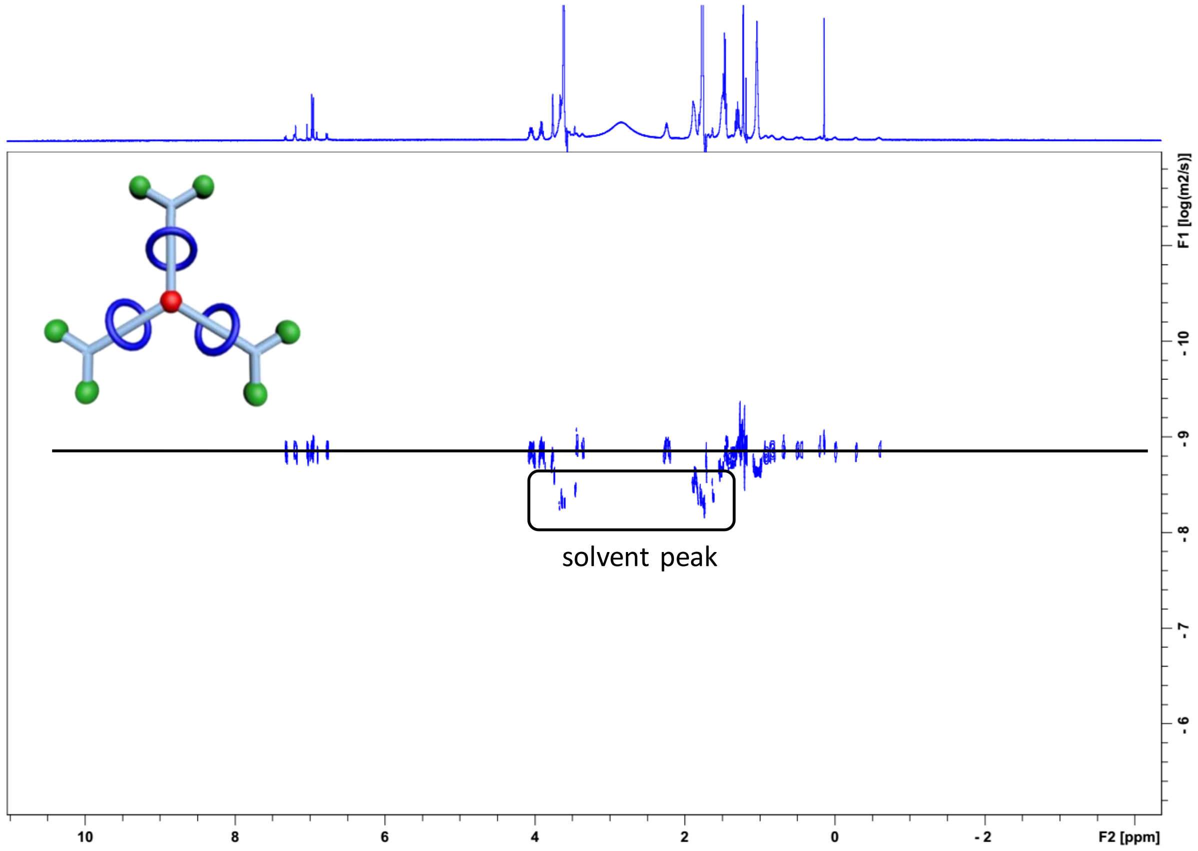

Supplementary Figure 101: 2-D DOSY spectrum (THF-d_8, 298 K, 500 MHz) of model rotaxane dendrimer **G$_1$-a** with the addition of 15.0 eq. of TBAA.

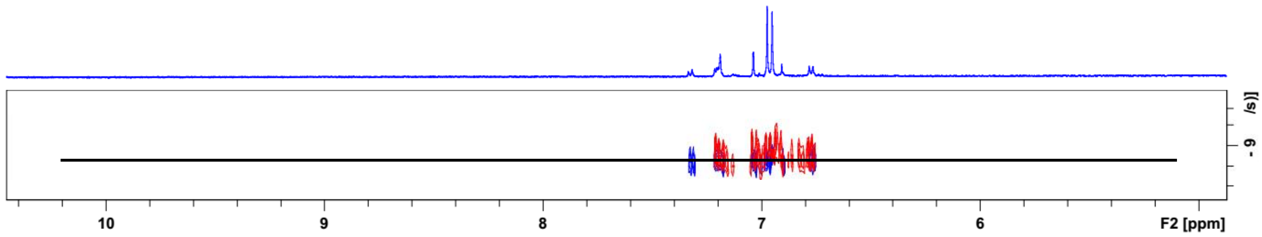

Supplementary Figure 102: Stacked DOSY spectra (THF-d_8, 298 K, 500 MHz) of model rotaxane dendrimer **G$_1$-a** (red) and **G$_1$-a** with the addition of 15.0 eq. of TBAA (blue).

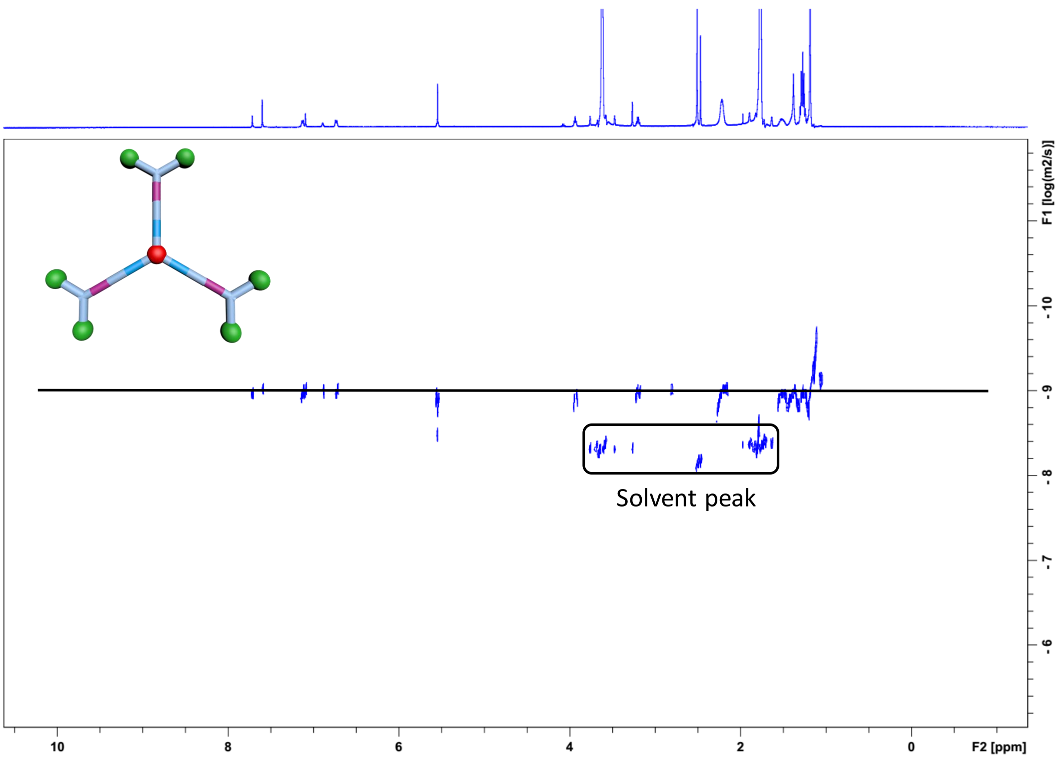

Supplementary Figure 103: 2-D DOSY spectrum (THF-d_8, 298 K, 500 MHz) of platinum-acetylide dendrimer **G$_1$-b**.

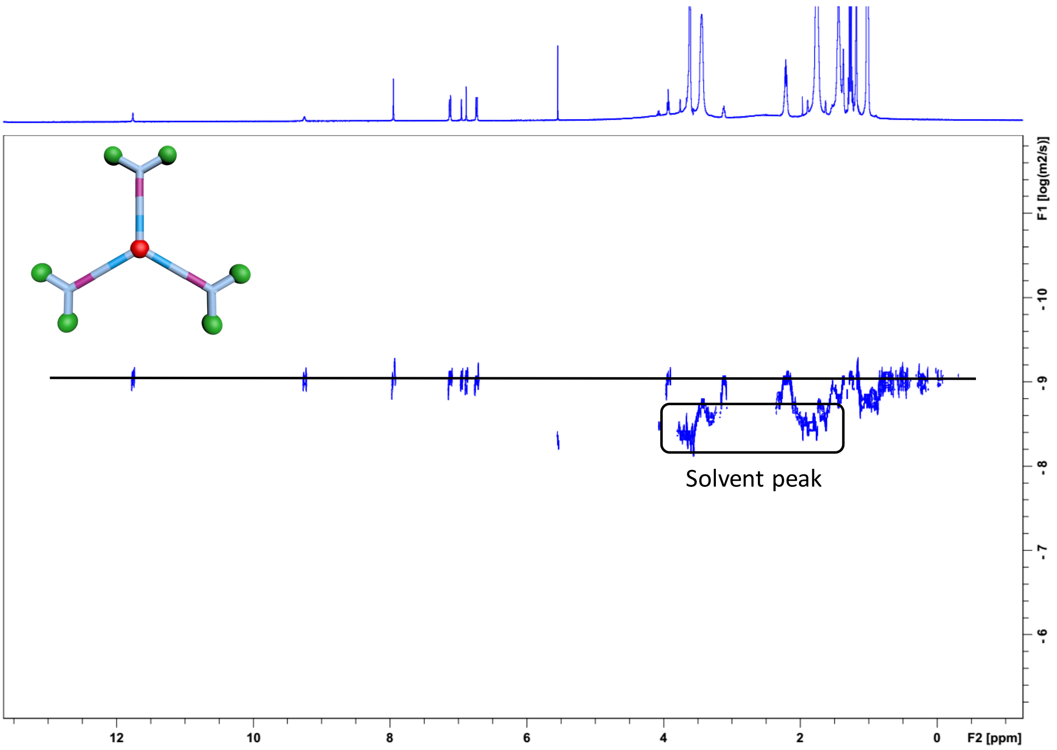

Supplementary Figure 104: 2-D DOSY spectrum (THF-d_8, 298 K, 500 MHz) of model platinum-acetylide dendrimer **G$_1$-b** with the addition of 15.0 eq. of TBAA.

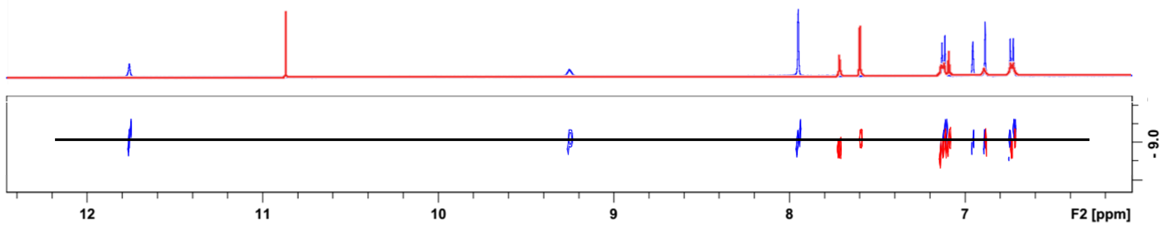

Supplementary Figure 105: Stacked DOSY spectra (THF-d_8, 298 K, 500 MHz) of model platinum-acetylide dendrimer **G$_1$-b** (red) and **G$_1$-b** with the addition of 15.0 eq. of TBAA (blue).

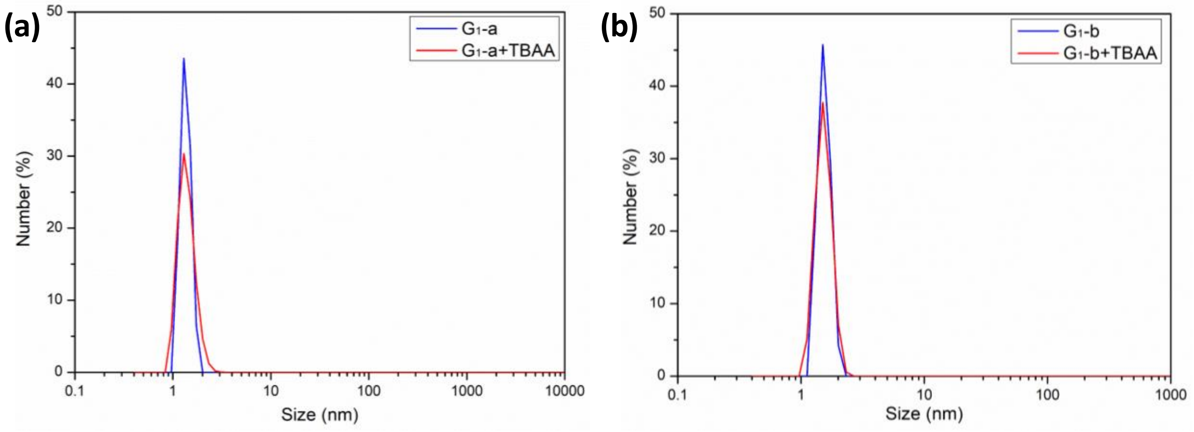

Supplementary Figure 106: DLS spectra of (a) model rotaxane dendrimer **G$_1$-a** and **G$_1$-a** with the addition of TBAA (15.0 eq.); (b) model dendrimer **G$_1$-b** and **G$_1$-b** with the addition of TBAA (15.0 eq.).

Supplementary Figure 107: Solvent- and anion-induced switching behavior of rotaxane-branched dendrimer $\mathbf{G_2}$.

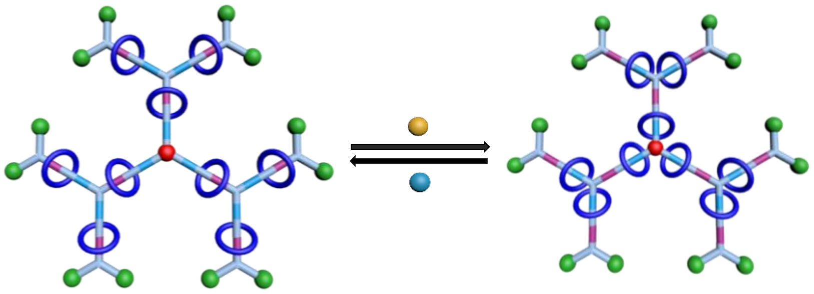

Supplementary Table 6: Solvent-induced chemical shift changes of partial protons of $\mathbf{G_2}$.

Solvent-induced chemical shift changes of partial protons of $\mathbf{G_2}$							
		H_3	H_4	H_{13}	H_{14}	H_{15}	H_{16}
1	THF-d_8, 400 μL	7.51	4.68	0.20	-0.63	-1.07	0.04
2	THF-d_8/DMSO-d_6, 400/2 μL	7.80	5.06	0.07	-0.88	-1.37	-0.09
3	THF-d_8/DMSO-d_6, 400/4 μL	7.96	5.28	-0.01	-1.01	-1.54	-0.19
4	THF-d_8/DMSO-d_6, 400/6 μL	8.06	5.40	-0.05	-1.08	-1.63	-0.25
5	THF-d_8/DMSO-d_6, 400/8 μL	8.14	5.51	-0.08	-1.14	-1.70	-0.30
6	THF-d_8/DMSO-d_6, 400/10 μL	8.19	5.58	-0.11	-1.19	-1.76	-0.34
7	THF-d_8/DMSO-d_6, 500/10 μL	8.11	5.47	-0.07	-1.12	-1.68	-0.28

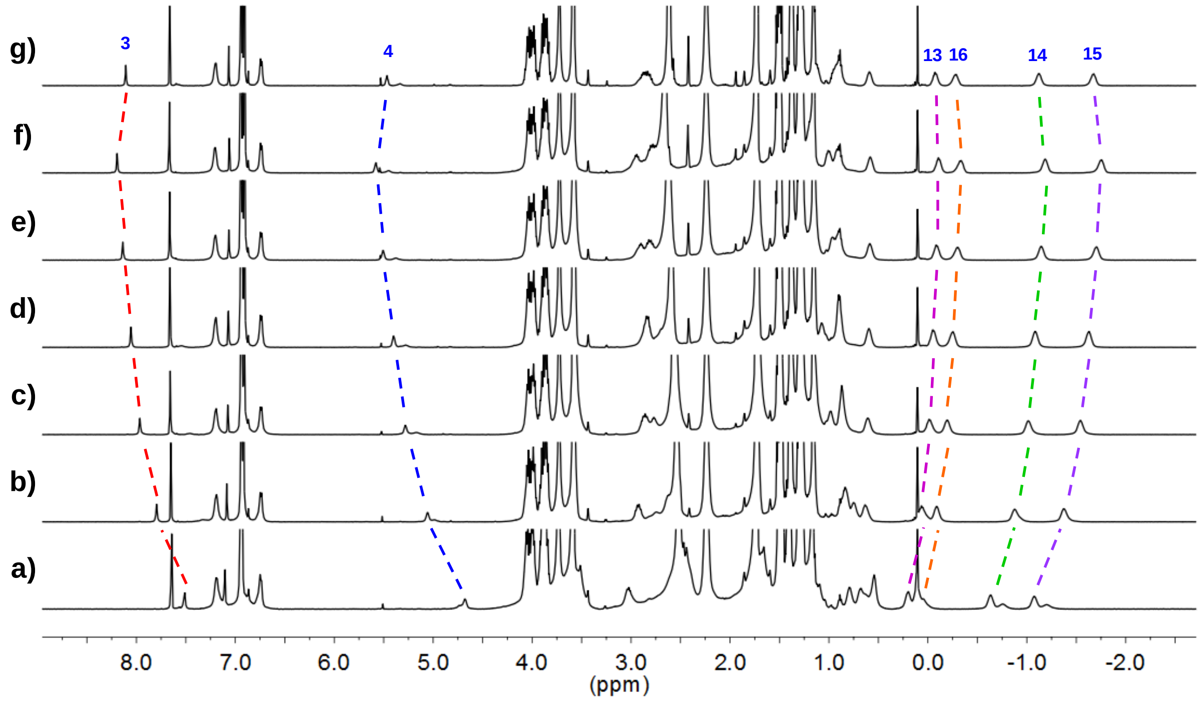

Supplementary Figure 108: ^{1}H NMR spectra (THF-d_8 (400 μL), 298 K, 500 MHz) of solvent-induced switching behavior of rotaxane-branched dendrimer $\mathbf{G_2}$. a) $\mathbf{G_2}$; the addition of DMSO-d_6 in a): b) 2 μL; c) 4 μL; d) 6 μL; e) 8 μL; f) 10 μL; g) the addition of THF-d_8 (100 μL) in f).

Supplementary Table 7: Anion-induced chemical shift changes of partial protons of **G_2**.

Anion-induced chemical shift changes of partial protons of G_2									
		H_3		H_4		H_{14-1}	H_{14-2}	H_{15-1}	H_{15-2}
a	**G_2**	7.53		4.69		-0.65	-0.76	-1.09	-1.22
b	**G_2**+TBAA (1 eq.)	10.47	8.39	7.92	6.12	-1.29	-1.03	-1.89	-1.54
c	**G_2**+TBAA (2 eq.)	11.24	9.55	8.79	*	-1.45	-1.28	-2.08	-1.85
d	**G_2**+TBAA (3 eq.)	11.41	9.99	8.99	*	-1.48	-1.37	-2.12	-1.97
e	**G_2**+TBAA (4 eq.)	11.46	10.12	9.04	8.06	-1.49	-1.39	-2.14	-2.01
f	**G_2**+TBAA (5 eq.)	11.49	10.23	9.08	8.17	-1.49	-1.41	-2.14	-2.03
g	**G_2**+TBAA+$NaPF_6$ (5 eq.)	7.90	7.67	5.15	4.88	-0.79	-0.79	-1.26	-1.26
h	**G_2**+TBAA+$NaPF_6$ (7 eq.)	7.64		4.86		-0.72	-0.80	-1.18	-1.25
i	**G_2**+TBAA+$NaPF_6$ (8 eq.)	7.63		4.84		-0.72	-0.80	-1.18	-1.26
j	**G_2**+TBAA+$NaPF_6$ (10 eq.)	7.62		4.84		-0.72	-0.80	-1.18	-1.26

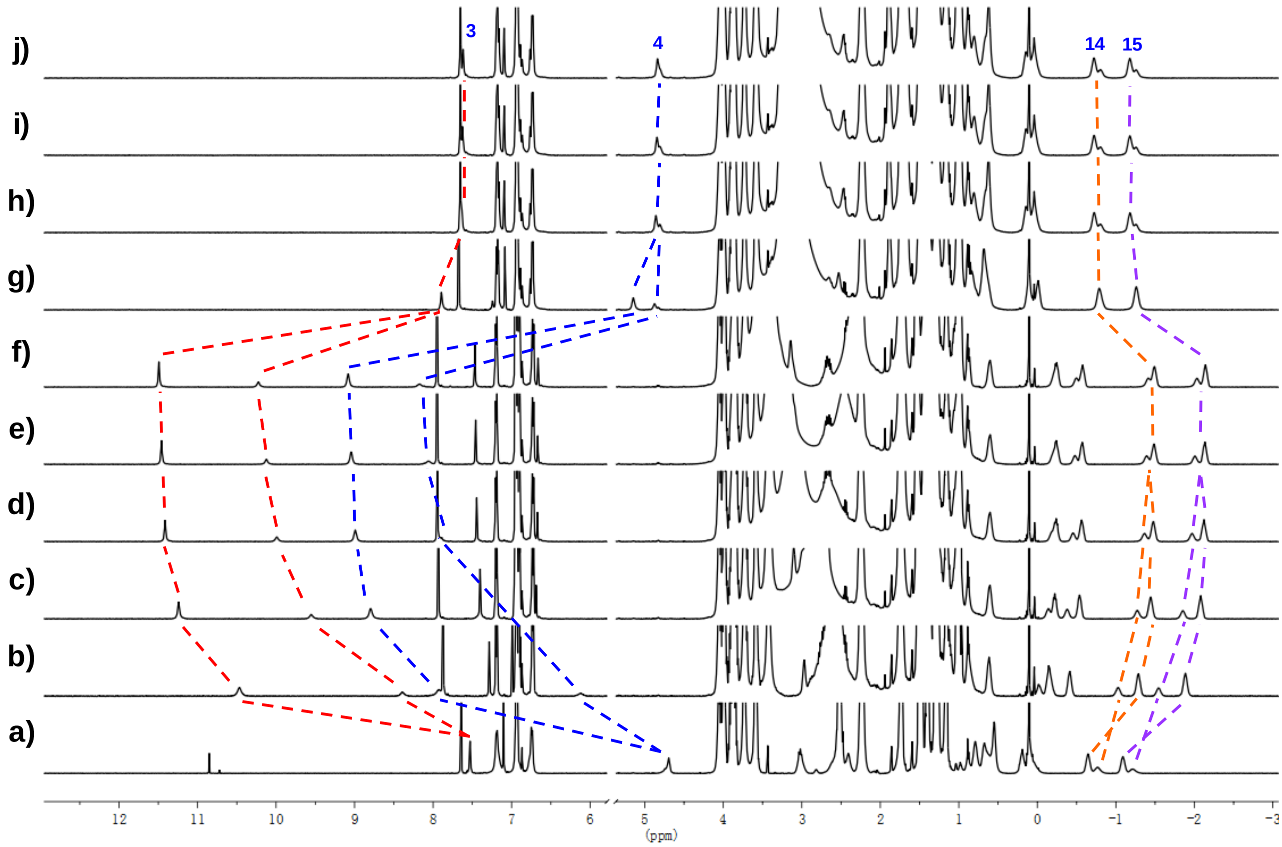

Supplementary Figure 109: ^{1}H NMR spectra (THF-d_8, 298 K, 500 MHz) of anion-induced switching behavior of rotaxane-branched dendrimer **G_2**. a) **G_2**; the mixture of **G_2** and TBAA, for each rotaxane unit: b) TBAA (1 equiv); c) TBAA (2 equiv); d) TBAA (3 equiv); e) TBAA (4 equiv); f) TBAA (5 equiv); and the mixture obtained after adding $NaPF_6$ to the solution in f), for each rotaxane unit: g) $NaPF_6$ (5 equiv); h) $NaPF_6$ (7 equiv); i) $NaPF_6$ (8 equiv); j) $NaPF_6$ (10 equiv).

Supplementary Figure 110: Solvent- and anion-induced switching behavior of rotaxane-branched dendrimer **G3**.

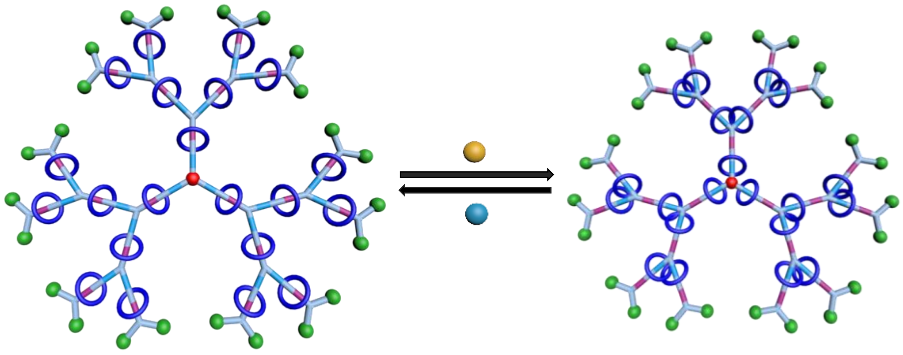

Supplementary Table 8: Solvent-induced chemical shift changes of partial protons of rotaxane-branched dendrimer **G3**.

Solvent-induced chemical shift changes of partial protons of **G**$_3$							
		H_3	H_4	H_{13}	H_{14}	H_{15}	H_{16}
1	THF-*d*$_8$, 400 μL	7.51	4.67	0.20	-0.63	-1.07	0.10
2	THF-*d*$_8$/DMSO-*d*$_6$, 400/2 μL	7.74	4.98	0.11	-0.82	-1.31	-0.05
3	THF-*d*$_8$/DMSO-*d*$_6$, 400/4 μL	7.92	5.22	-0.02	-0.97	-1.49	-0.17
4	THF-*d*$_8$/DMSO-*d*$_6$, 400/6 μL	8.03	5.37	-0.03	-1.06	-1.61	-0.25
5	THF-*d*$_8$/DMSO-*d*$_6$, 400/8 μL	8.12	5.48	-0.07	-1.13	-1.69	-0.30
6	THF-*d*$_8$/DMSO-*d*$_6$, 400/10 μL	8.18	5.56	-0.09	-1.17	-1.74	-0.33
7	THF-*d*$_8$/DMSO-*d*$_6$, 500/10 μL	8.10	5.46	-0.06	-1.11	-1.67	-0.29

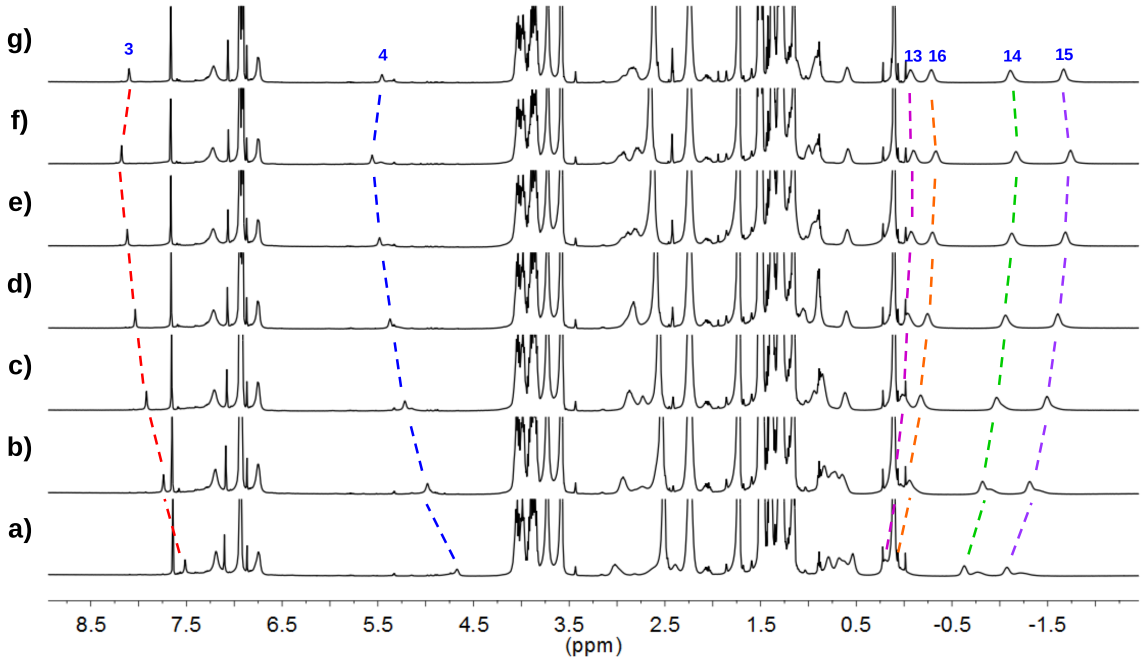

Supplementary Figure 111: ^{1}H NMR spectra (THF-*d*$_8$ (400 μL), 298 K, 500 MHz) of solvent-induced switching behavior of rotaxane-branched dendrimer **G3**. a) **G3**; the addition of DMSO-*d*$_6$ in a): b) 2 μL; c) 4 μL; d) 6 μL; e) 8 μL; f) 10 μL; g) the addition of THF-*d*$_8$ (100 μL) in f).

Supplementary Table 9: Anion-induced chemical shift changes of partial protons of **G3**.

Anion-induced chemical shift changes of partial protons of $\mathbf{G_3}$									
		H_3		H_4		$H_{14\text{-}1}$	$H_{14\text{-}2}$	$H_{15\text{-}1}$	$H_{15\text{-}2}$
a	$\mathbf{G_3}$	7.51		4.67		-0.63	-0.77	-1.07	-1.22
b	$\mathbf{G_3}$+TBAA (1 eq.)	10.52	8.54	8.00	6.31	-1.32	-1.09	-1.93	-1.63
c	$\mathbf{G_3}$+TBAA (2 eq.)	11.06	9.38	8.62	*	-1.43	-1.27	-2.08	-1.86
d	$\mathbf{G_3}$+TBAA (3 eq.)	11.27	9.86	8.85	7.77	-1.47	-1.37	-2.13	-1.99
e	$\mathbf{G_3}$+TBAA (4 eq.)	11.34	10.06	8.94	8.02	-1.49	-1.41	-2.15	-2.04
f	$\mathbf{G_3}$+TBAA (5 eq.)	11.39	10.17	8.97	8.11	-1.49	-1.43	-2.15	-2.07
g	$\mathbf{G_3}$+TBAA+$NaPF_6$ (5 eq.)	9.27	7.98	*	*	-1.12	-0.97	-1.69	-1.47
h	$\mathbf{G_3}$+TBAA+$NaPF_6$ (7 eq.)	7.71	7.66	4.95	4.85	-0.77		-1.24	
i	$\mathbf{G_3}$+TBAA+$NaPF_6$ (8 eq.)	7.65		4.88		-0.75		-1.22	
j	$\mathbf{G_3}$+TBAA+$NaPF_6$ (10 eq.)	7.65		4.87		-0.75		-1.22	

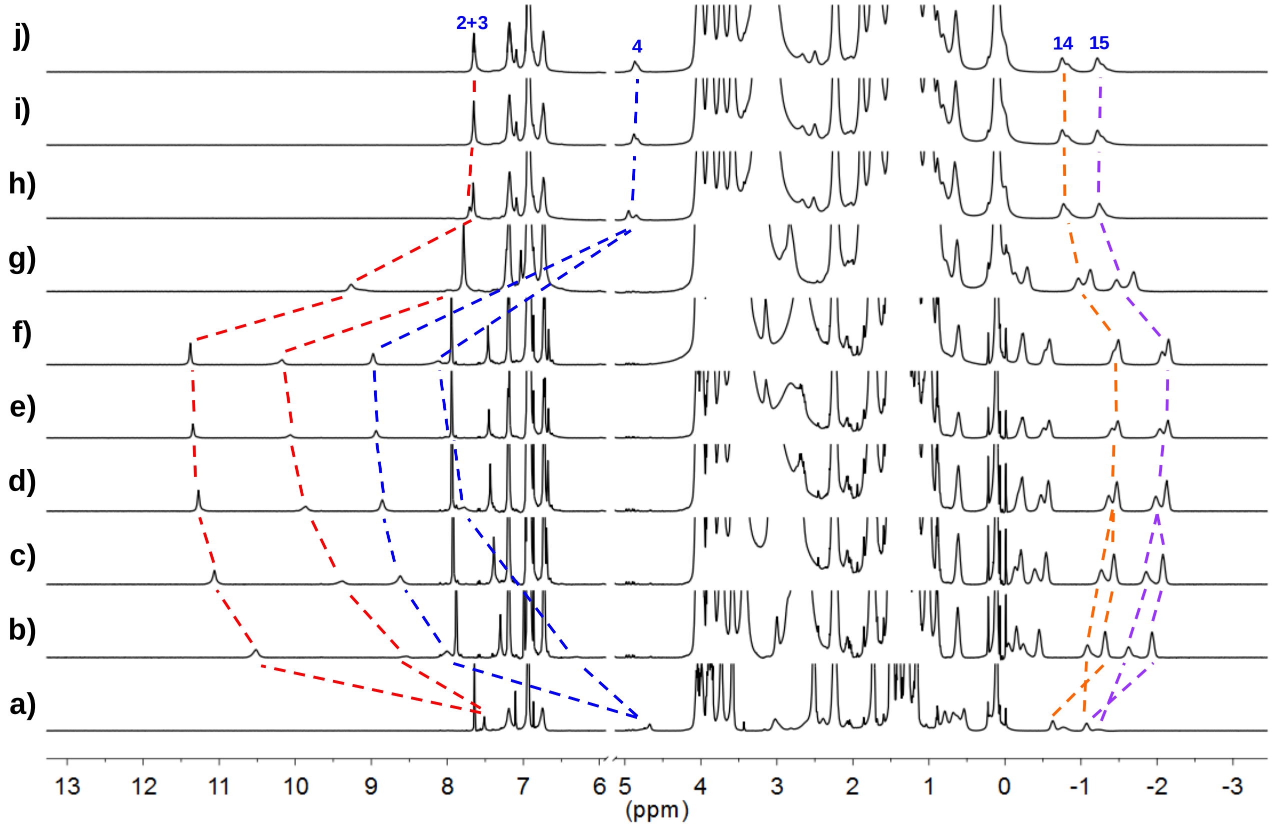

Supplementary Figure 112: ^{1}H NMR spectra (THF-d_8, 298 K, 500 MHz) of anion-induced switching motion of rotaxane-branched dendrimer **G3**. a) **G3**; the mixture of **G3** and TBAA, for each rotaxane unit: b) TBAA (1 equiv); c) TBAA (2 equiv); d) TBAA (3 equiv); e) TBAA (4 equiv); f) TBAA (5 equiv); and the mixture obtained after adding $NaPF_6$ to the solution in f), for each rotaxane unit: g) $NaPF_6$ (5 equiv); h) $NaPF_6$ (7 equiv); i) $NaPF_6$ (8 equiv); j) $NaPF_6$ (10 equiv).

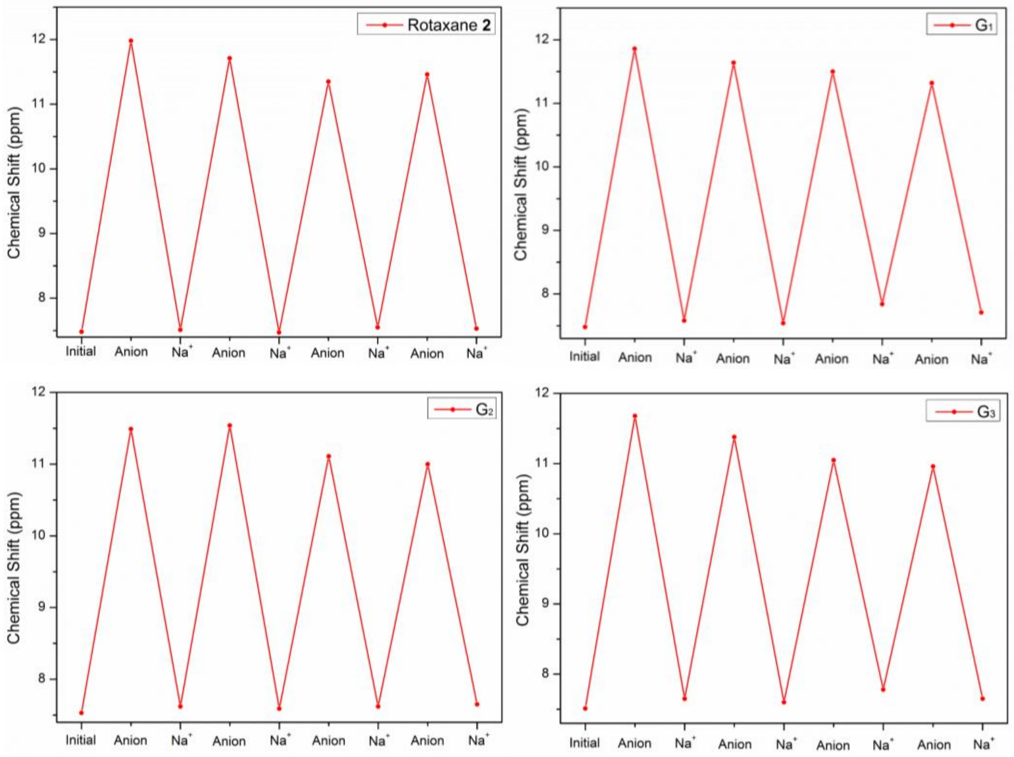

Supplementary Figure 113: Cycling experiments of anion-induced switching motions of [2]rotaxane building block **2** and rotaxane-branched dendrimers $\mathbf{G_1}$-$\mathbf{G_3}$.

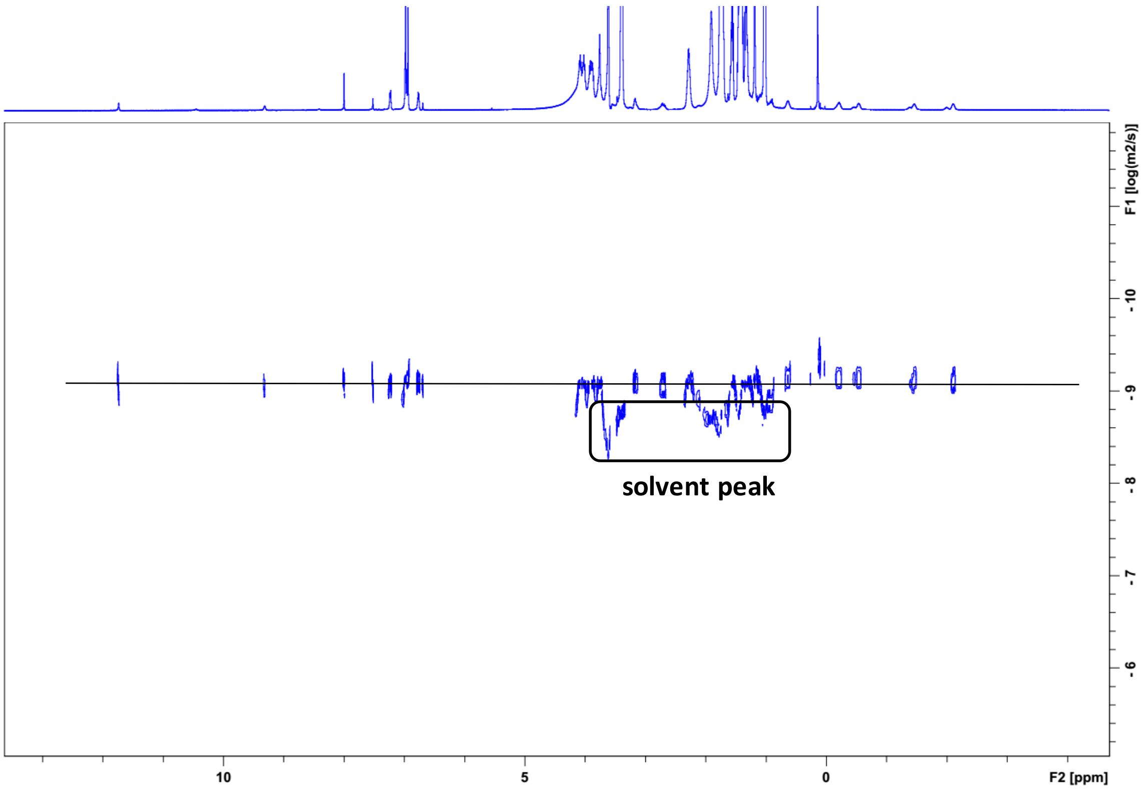

Supplementary Figure 114: 2-D DOSY spectrum (THF-d_8, 298 K, 500 MHz) of rotaxane-branched dendrimer $\mathbf{G_2}$ with the addition of TBAA (5 equiv. for each urea unit).

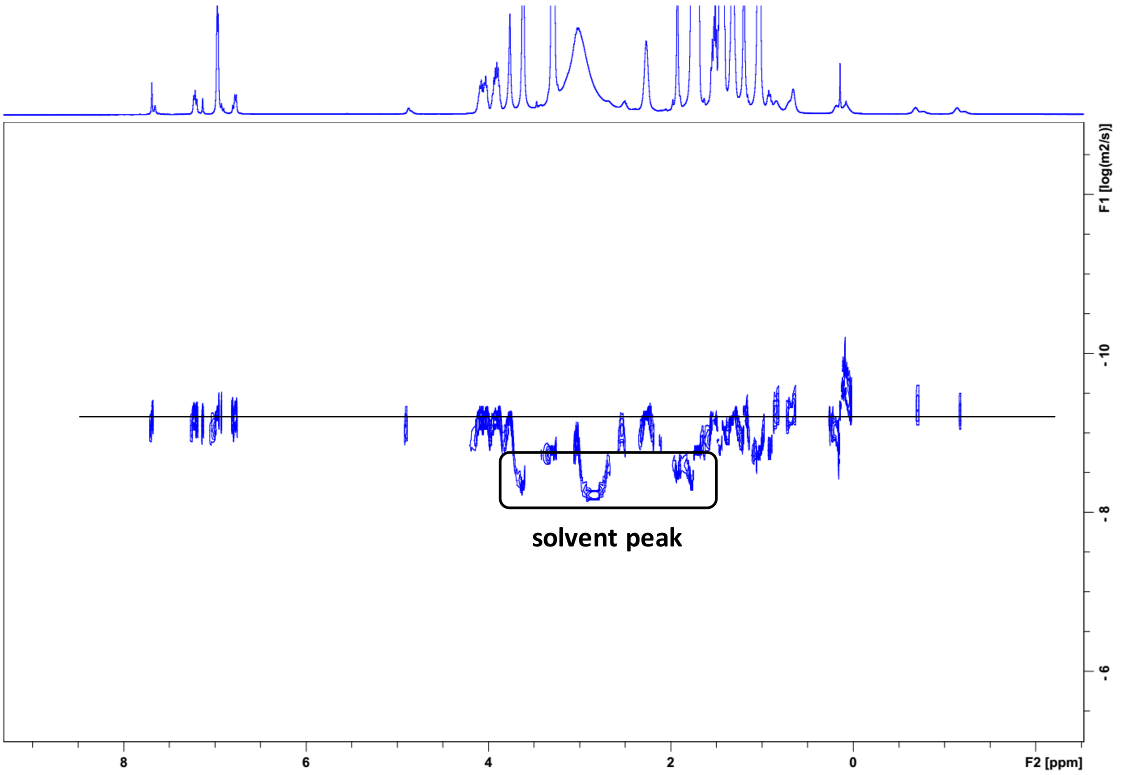

Supplementary Figure 115: 2-D DOSY spectrum (THF-d_8, 298 K, 500 MHz) of the mixture after adding $NaPF_6$ into the solution of rotaxane-branched dendrimer **G2** and TBAA.

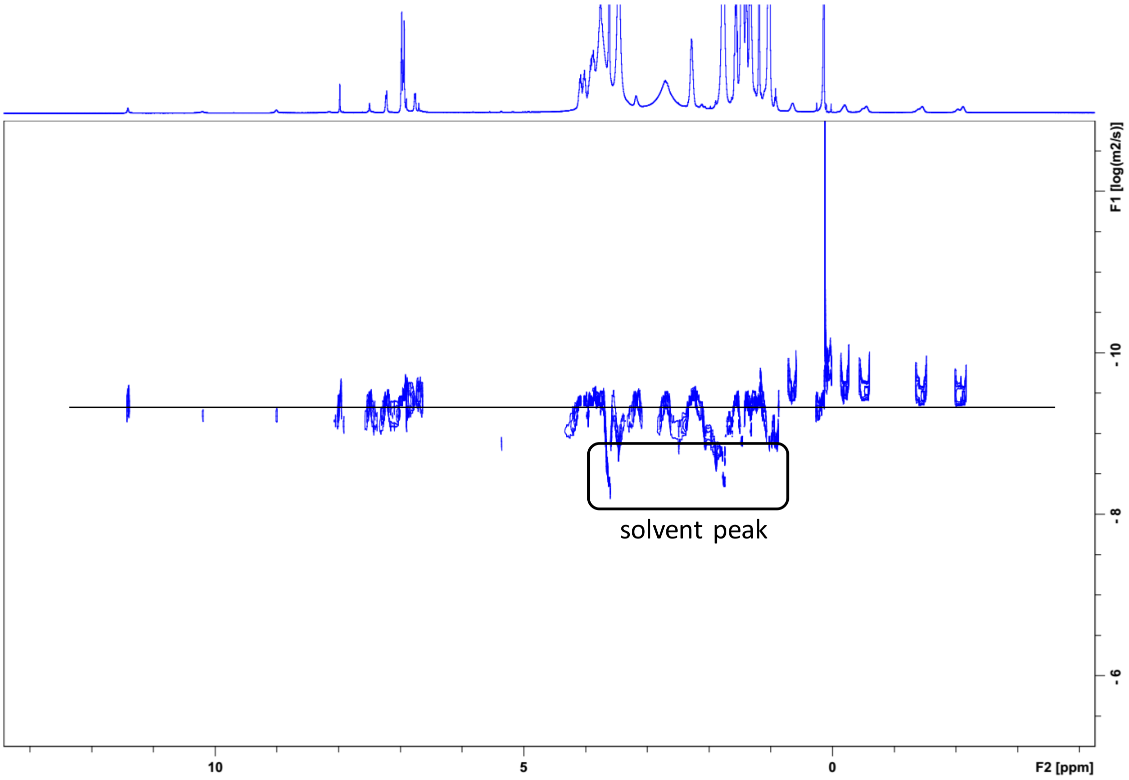

Supplementary Figure 116: 2-D DOSY spectrum (THF-d_8, 298 K, 500 MHz) of rotaxane-branched dendrimer **G3** with the addition of TBAA (5 equiv. for each urea unit).

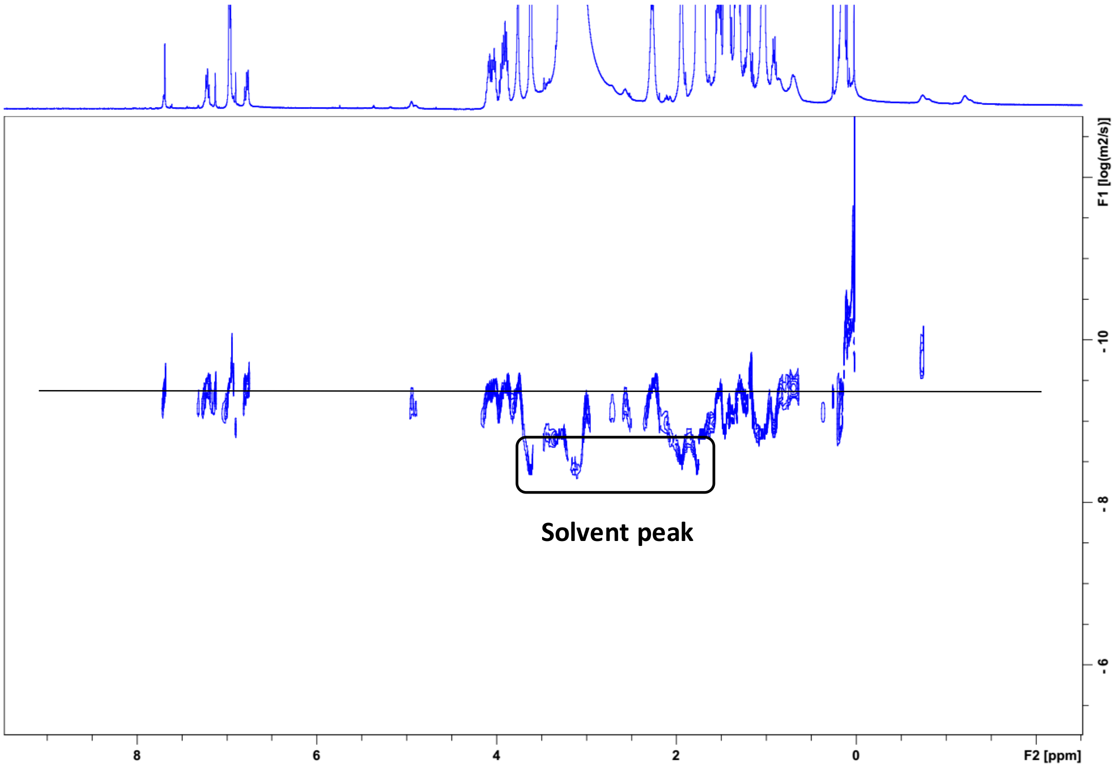

Supplementary Figure 117: DOSY spectrum (THF-d_8, 298 K, 500 MHz) of the mixture after adding $NaPF_6$ into the solution of rotaxane-branched dendrimer $\mathbf{G_3}$ and TBAA.

Supplementary Table 10: DLS data of anion-induced size switching of rotaxane-branched dendrimers **G_1-G_3**.

	**G_1**	G_1+TBAA	G_1+TBAA+Na^+
Size (d.nm)	2.03	1.63	2.01
	**G_2**	G_2+TBAA	G_2+TBAA+Na^+
Size (d.nm)	3.14	2.28	2.81
	**G_3**	G_3+TBAA	G_3+TBAA+Na^+
Size (d.nm)	4.51	2.75	3.79

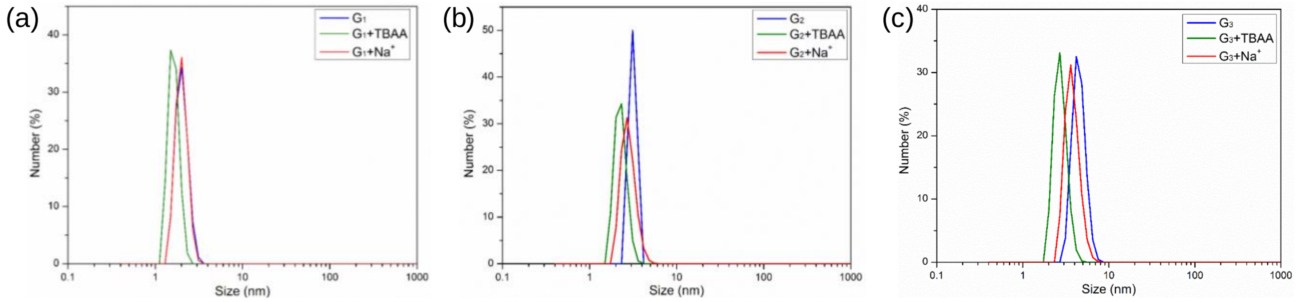

Supplementary Figure 118: DLS spectra of (a) anion-induced size switching of rotaxane-branched dendrimer **G_1**; (b) anion-induced size switching of rotaxane-branched dendrimer **G_2**; (c) anion-induced size switching of rotaxane-branched dendrimer **G_3**.

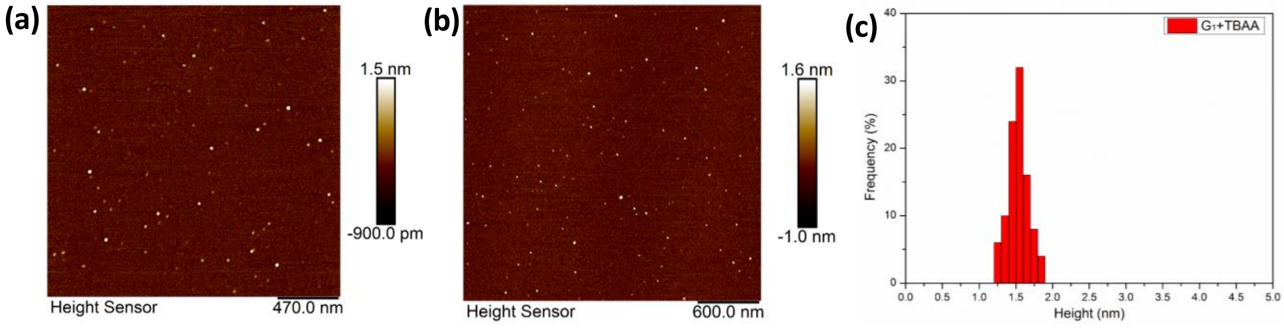

Supplementary Figure 119: (a-b) AFM images and (c) height distributions of rotaxane-branched dendrimer $\mathbf{G_1}$ with the addition of TBAA (15 eq.), the height range is 1.52 ±0.29 nm.

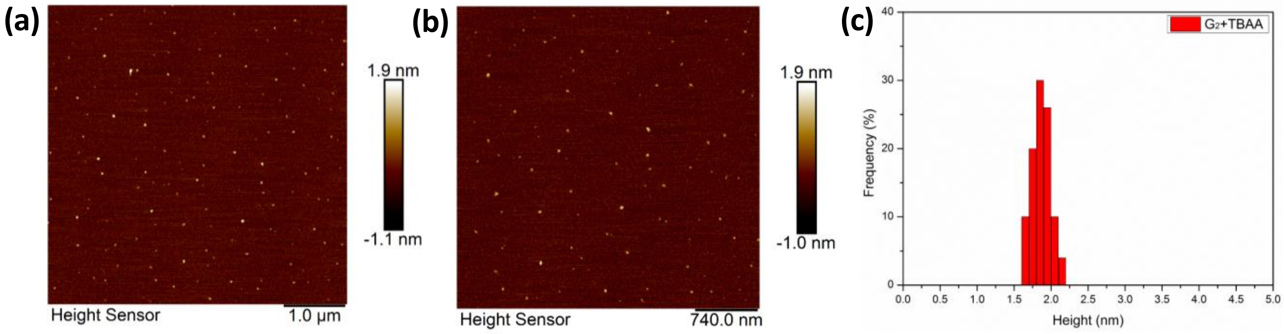

Supplementary Figure 120: (a-b) AFM images and (c) height distributions of rotaxane-branched dendrimer $\mathbf{G_2}$ with the addition of TBAA (45 eq.), the height range is 1.85 ±0.25 nm.

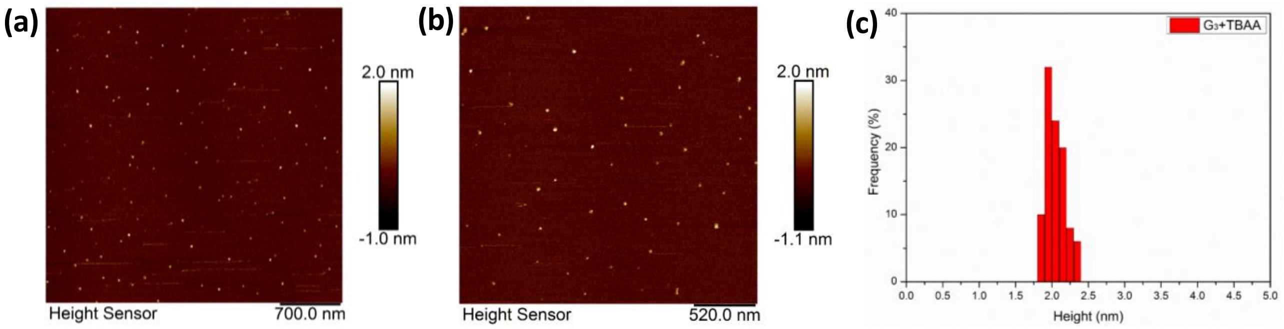

Supplementary Figure 121: (a-b) AFM images and (c) height distributions of rotaxane-branched dendrimer $\mathbf{G_3}$ with the addition of TBAA (105 eq.), the height range is 2.05 ±0.30 nm.

Section G. Supplementary References

1. Lewis, D. G. et al. *Proc. SPIE*. **7755**, 775505/1-775505/10 (2010).

2. Kong, X. et al. *Org. Lett*. **13**, 764-767 (2011).

3. Wang, K. et al. *Org. Biomol. Chem*. **10**, 9405-9409 (2012).
